# Autophagy Receptor-Inspired Antibody-Fusion Proteins for Targeted Intracellular Degradation

**DOI:** 10.1021/jacs.3c05199

**Published:** 2023-09-25

**Authors:** Ziwen Jiang, Yu-Hsuan Kuo, Michelle R. Arkin

**Affiliations:** Department of Pharmaceutical Chemistry and Small Molecule Discovery Center, University of California, San Francisco, California 94158, United States

## Abstract

Autophagy is responsible for the degradation of large intracellular contents, such as unwanted protein aggregates and organelles. Impaired autophagy can therefore lead to the accumulation of pathological aggregates, correlating with aging and neurodegenerative diseases. However, a broadly applicable methodology is not available for the targeted degradation of protein aggregates or organelles in mammalian cells. Herein, we developed a series of autophagy receptor-inspired targeting chimeras (AceTACs) that can induce the targeted degradation of aggregation-prone proteins and protein aggregates (e.g., huntingtin, TDP-43, and FUS mutants), as well as organelles (e.g., mitochondria, peroxisomes, and endoplasmic reticulum). These antibody-fusion-based AceTAC degraders were designed to mimic the function of autophagy receptors, simultaneously binding with the cellular targets and the LC3 proteins on the autophagosomal membrane, eventually transporting the target to the autophagy-lysosomal process for degradation. The AceTAC degradation system provides design principles for antibody-based degradation through autophagy, largely expanding the scope of intracellular targeted degradation technologies.

## Graphical Abstract

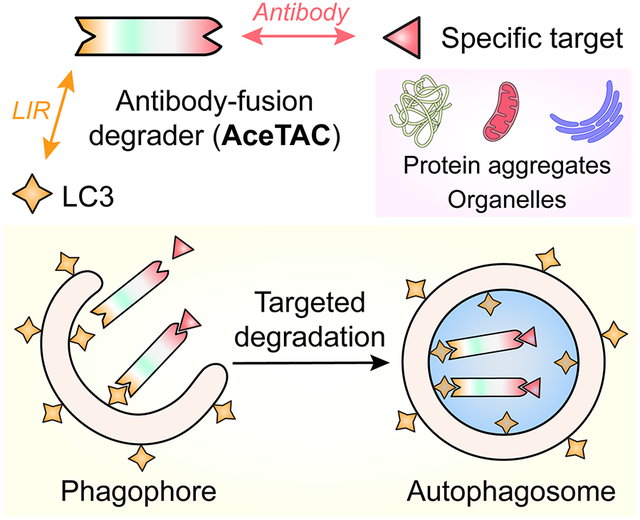

## INTRODUCTION

Cellular homeostasis requires the degradation of abnormal protein aggregates and damaged organelles.^1,2^ The accumulation of impaired protein aggregates and organelles has been linked with aging and neurodegenerative diseases.^3,4^ For example, tau aggregation is a hallmark of Alzheimer’s disease^5^ and the aberrant aggregation of TAR DNA-binding protein 43 kDa (TDP-43) has been identified as a defining pathological feature for amyotrophic lateral sclerosis (ALS).^6^ The accumulation of defective mitochondria can cause severe cell and tissue damage as observed in patients with Parkinson’s disease.^7^ It is thus important to maintain an efficient clearance of unwanted large-sized intracellular contents.

In living cells, dysfunctional protein aggregates, organelles, and pathogenic microorganisms are degraded through autophagy.^8^ Autophagy initiates the degradation of intracellular contents through the formation of the double-membrane autophagosomes, followed by fusion with lysosomes and proteolytic degradation of the engulfed substrates.^9^ Unlike the ubiquitin–proteasome system (UPS) where proteins are the major substrates for degradation, the autophagy-lysosomal pathway (ALP) is the dedicated machinery for the degradation of large-sized intracellular contents.^10^ Hence, defective autophagy has been associated with diverse human diseases,^11^ correlating with the failure to degrade cytotoxic aggregates and disruptions to cellular homeostasis. The acceleration or restoration of ALP therefore potentially provides strategies for therapeutic development.

There are two major limitations within the available strategies to regulate the degradation of impaired protein aggregates and organelles. First, small-molecule drugs that modulate autophagy are not specific toward dysfunctional targets.^12^ These autophagy modulators (e.g., Torin-1) affect the overall level of autophagy and cause off-target concerns. Second, a majority of techniques for the targeted degradation of intracellular contents are introduced through the UPS. The targets of such methods are mainly proteins (e.g., PROTACs^13^), as the UPS cannot efficiently process complex and large-sized contents. Therefore, a technological gap remains in the targeted degradation of large-sized substrates. In recent years, several targeted degradation techniques through the ALP have emerged,^14,15^ yet a universal strategy to the targeted degradation of protein aggregates and organelles is not available in mammalian cells.

**Scheme 1.**
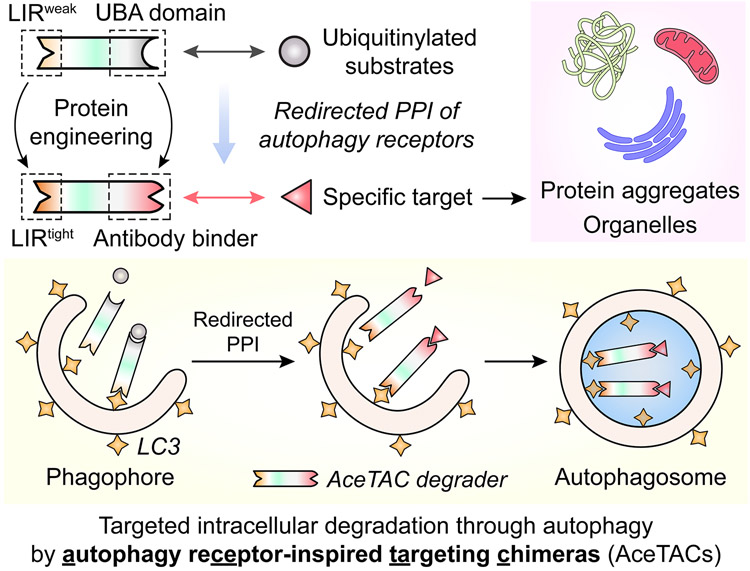
Workflow of the AceTAC Degraders for Targeted Degradation of Protein Aggregates and Organelles^a^ ^a^LIR, LC3-interacting region; PPI, protein–protein interaction.

During autophagy, the selection of autophagosomal substrates is aided by autophagy receptors, a family of proteins that simultaneously interact with both the substrates and autophagosomal membranes.^16^ For example, sequestosome-1 (SQSTM1/p62) is a major mammalian autophagy receptor.^17^ The ubiquitin-associated (UBA) domain of p62 interacts with ubiquitinylated (Ub-) substrates to form aggregates during selective autophagy. Next, these aggregates are driven into the forming autophagosome through the interaction between the LC3-interacting region (LIR) of p62 and the LC3/GABARAP proteins on the autophagosomal membranes. Based on the protein–protein interactions of p62 during autophagy, we herein report the development of autophagy receptor-inspired targeting chimeras (AceTACs) (Scheme 1). The design of AceTAC was developed through the protein engineering of three generations of antibody (Ab)-fusion degraders, resulting in an efficient method to transport the targeted protein into the autophagy process for eventual degradation. Furthermore, the AceTAC degradation technology was customized for targeted degradation of specific organelles, including mitochondria, peroxisomes, and the endoplasmic reticulum (ER), demonstrating its broad potential as a targeted degradation platform.

## RESULTS AND DISCUSSION

### Engineered Antibody-Fusion Degraders Lead to Efficient Degradation of Huntingtin Protein with Polyglutamine (polyQ) Expansion

**Figure 1.**
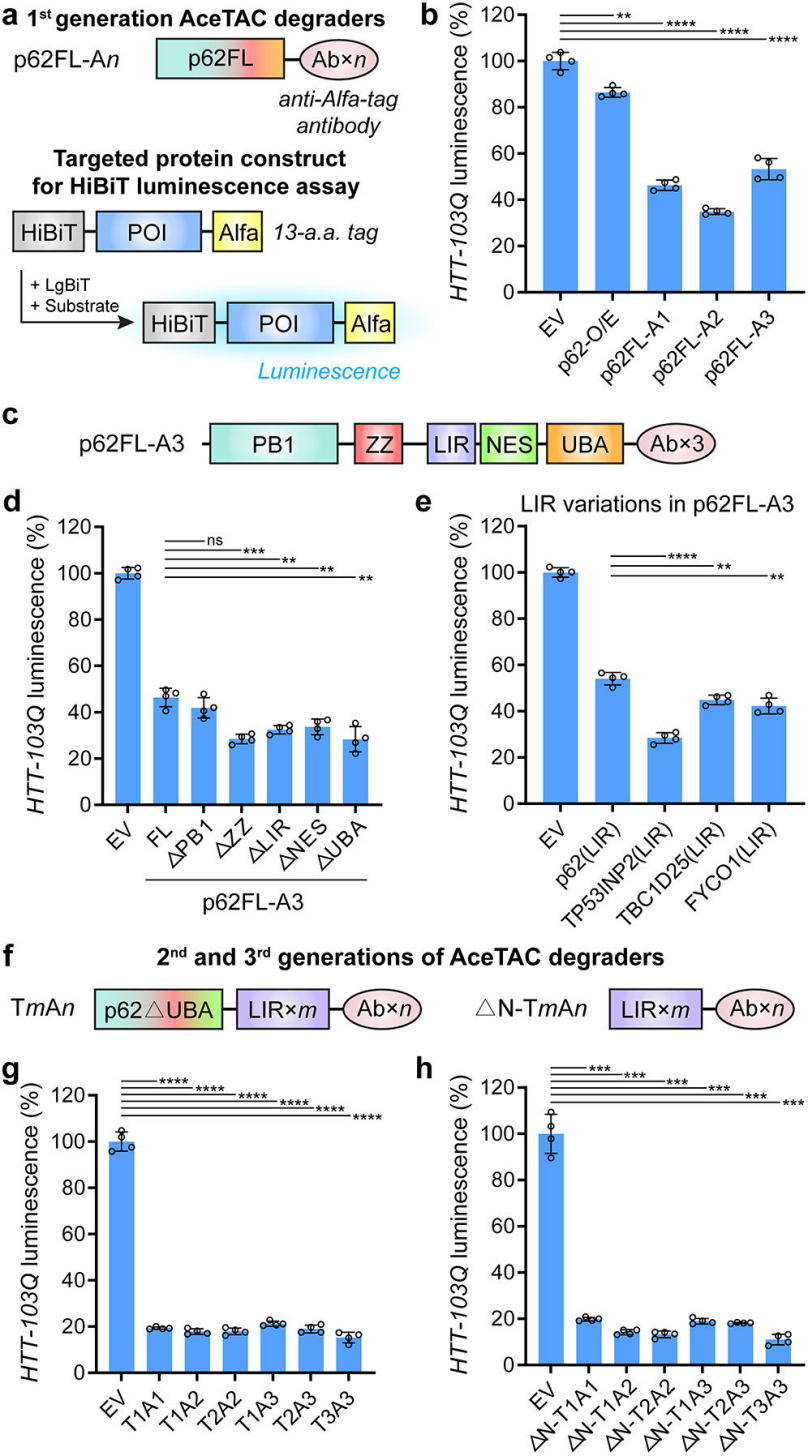
Development of AceTAC degraders for targeted HTT-103Q degradation through protein engineering. (a) Schematic illustrations of the first generation of AceTAC degraders (p62FL-A*n*, where *n* represents the number of antibody units) and the workflow of the HiBiT luminescence assay for the quantification of targeted protein degradation. (b) Targeted degradation of HTT-103Q by the first-generation AceTAC degraders. (c) Schematic illustration of the first generation AceTAC degrader (p62FL-A3) highlighting the functional domains/motifs of full-length p62. (d, e) Targeted degradation of HTT-103Q by the deletion mutants of p62FL-A3 degraders (d) and the p62FL-A3 constructs with LIR motifs derived from different LC3-binding proteins (e). (f) Schematic illustrations of the second (T*m*A*n*) and third generation (ΔN-T*m*A*n*) of AceTAC degraders. For both T*m*A*n* and ΔN-T*m*A*n*, *m* represents the number of TP53INP2-LIR motifs and *n* represents the number of antibody units. (g, h) Targeted degradation of HTT-103Q by the T*m*A*n* degraders (g) and the ΔN-T*m*A*n* degraders (h). Error bars represent standard deviations of *N* = 4. Statistical analyses are performed using two-tailed Student’s *t* test: **, *p* < 0.01; ***, *p* < 0.001; ****, *p* < 0.0001; ns, no significance.

The first generation of our Ab-fusion degraders was designed by directly linking target-specific antibodies to the C-terminus of full-length p62 (p62FL). We hypothesized that keeping the wild-type structure of p62 would minimally disturb the function of p62 during selective autophagy while accumulating the target of interest into the autophagic process through the interaction between the antibody and the target. We employed huntingtin protein with polyQ expansion as the degradation target with an N-terminal HiBiT peptide tag and a C-terminal ALFA-epitope tag (HTT-103Q). The N-terminal HiBiT tag allowed the quantification of HTT-103Q level upon the addition of LgBiT protein and luminescence substrate (Figure 1a).^18^ The C-terminal ALFA-tag was presented as the recognition site for the high affinity nanobodies (*K*_D_ ~ 26 pM ^19^) within the AceTAC degraders. Both the degrader-encoding plasmid and the target-encoding plasmid were co-transfected into human osteosarcoma (U2OS) cells to assess the efficiency of targeted degradation.

In the degrader design, we also incorporated antibody tandem repeats to evaluate the effect of the multivalency between the degrader and the target (Figure 1a). In the HiBiT luminescence assay, the cells in control (EV) group were co-transfected with the protein target-encoding plasmid and an empty vector. The luminescence value from the EV group was normalized to 100%. All three p62FL-based Ab-fusion degraders (p62FL-A*n*, where *n* represents the number of antibody units) demonstrated more than 50% degradation of HTT-103Q compared with the EV-control group (Figure 1b). Among these three degraders, p62FL-A2 induced the highest degradation efficiency (~65%) for HTT-103Q. The overexpression of p62 (p62-O/E) resulted in less than 15% degradation of HTT-103Q, indicating that the specific binding to HTT-103Q via the antibody in the AceTAC degraders was required for efficient degradation. The successful targeted degradation of HTT-103Q by these p62FL-Ab-fusion degraders indicated feasibility of AceTAC for targeted protein degradation, albeit with incomplete efficiency.

### The LIR Motif of TP53INp2 in the AceTAC Degrader Improves the Targeted Degradation Efficacy

We sought to determine the critical domains and motifs of the AceTAC degrader that affected its targeted degradation capability. Protein engineering of the AceTAC degrader evaluated (a) deletion mutants of p62 and (b) replacement of the LIR motif. For the deletion mutants, truncations of five domains/motifs were separately generated based on p62FL-A3. Previous reports have shown the participation of these five p62 domains and motifs during selective autophagy (Figure 1c). Apart from the described function of the LIR motif and UBA domain, (1) the Phox and Bem1 (PB1) domain is responsible for mediating the self-oligomerization of p62 and facilitating the coaggregation with autophagosomal substrates;^20^ (2) the ZZ-type zinc finger (ZZ) domain recognizes specific autophagosomal substrates via the N-degron pathway;^21^ (3) the nuclear export signal (NES) motif modulates the nucleocytoplasmic shuttling of p62.^22^ Surprisingly, four of the truncations (p62FL-A3^ΔZZ^, p62FL-A3^ΔLIR^, p62FL-A3^ΔNES^, and p62FL-A3^ΔUBA^) increased the degradation of HTT-103Q by at least 13% (Figure 1d). Deletion of the PB1 domain had no effect, though it has been shown to be important for endogenous autophagy.^23^ Such a result indicated that the PB1 domain for AceTAC may not have been as important as it was for wild-type p62 during the autophagy process.

We next evaluated the effect of LIR-motif variations in the p62FL-A3 degrader on the targeted protein degradation. Previous studies have tested the binding between LC3/ GABARAP proteins and the LIR motifs from various binding partners.^24^ Based on the report, we picked three LIR motifs (TP53INP2, TBC1D25, and FYCO1) that tightly and broadly bound with six LC3/GABARAP proteins. After the p62-LIR motif in the p62FL-A3 degrader was replaced with the TP53INP2-LIR motif, the targeted degradation efficiency for HTT-103Q was significantly improved by ~26%, resulting in ~72% degradation of HTT-103Q when compared to the EV group (Figure 1e). The improved degradation efficiency might have been due to the LIR motif of TP53INP2 binding the most efficiently with all six LC3/GABARAP proteins when compared to 33 other tested LIR motifs.^24^ Meanwhile, the engineered degrader that contained either TBC1D25-LIR or FYCO1-LIR achieved ~10% more degradation of HTT-103Q than that with p62-LIR, i.e., p62FL-A3. Although the absence of the p62-LIR motif (i.e., the p62FL-A3^ΔLIR^ group) still surprisingly resulted in the degradation of HTT-103Q (Figure 1d), the addition of efficient LIR motifs to the degrader could significantly increase its degradation efficiency. These comparisons demonstrated that the LIR motif on the degrader could modulate its targeted degradation efficacy.

### Multivalent Constructs of the AceTACs Further Enhance the Targeted Degradation Efficacy

Based on degradation results by the deletion mutants and the LIR variations, we constructed two more generations of the AceTAC degraders (Figure 1f). The design principle of these new degraders centered on repeats of the TP53INP2-LIR motif and the antibody. In detail, the second generation of AceTACs was built on p62^ΔUBA^ with its LIR motif replaced by TP53INP2-LIR motif(s), followed by antibody unit(s) on its C-terminus (T*m*A*n*, where *m* represents the number of TP53INP2-LIR motifs, *n* represents the number of antibody repeats). The third generation of AceTACs was a simplified version of the second generation; it only contained repeats of the LIR motif and the antibody (ΔN-T*m*A*n*) (Figure S1).

To evaluate the differences between these two generations of AceTACs, we stepwise generated the deletion mutants of T3A3, including T3A3^ΔPB1^, T3A3^ΔPB1ΔZZ^, and ΔN-T3A3. Consistent with the deletion mutants of p62FL-A3 (Figure 1d), the deletion of the PB1 domain in T3A3 did not significantly affect the degradation performance (Figure S2). Improved HTT-103Q degradation efficacy was achieved by ΔN-T3A3 compared to T3A3. For the overall performance of these two generations of AceTAC degraders, at least 80% of HTT-103Q degradation was achieved (Figure 1g,h). However, no obvious trend of targeted degradation was observed in the correlation with the number of LIR motifs or antibody units. Among these degraders, ΔN-T3A3 consistently achieved ~90% targeted degradation of HTT-103Q. The high efficiency of ΔN-T*m*A*n* degraders for targeted HTT-103Q degradation suggested that the p62 domains and motifs may not be necessary for the AceTAC degradation system. Only the LIR motif and the target-specific antibody were retained in the third generation AceTAC degraders. Additionally, the ALFA-tagged HTT-103Q mRNA levels remained similar in the presence of three generations of AceTAC degraders (Figure S3a). The targeted degradation of HTT-103Q could also be modulated in a doxycycline-inducible expression system for these three generations of AceTAC degraders (Figure S4). These results confirmed that degradation was induced at the protein level.

### AceTAC Is Applicable for the Targeted Degradation of Various Aggregation-Prone Proteins

**Figure 2.**
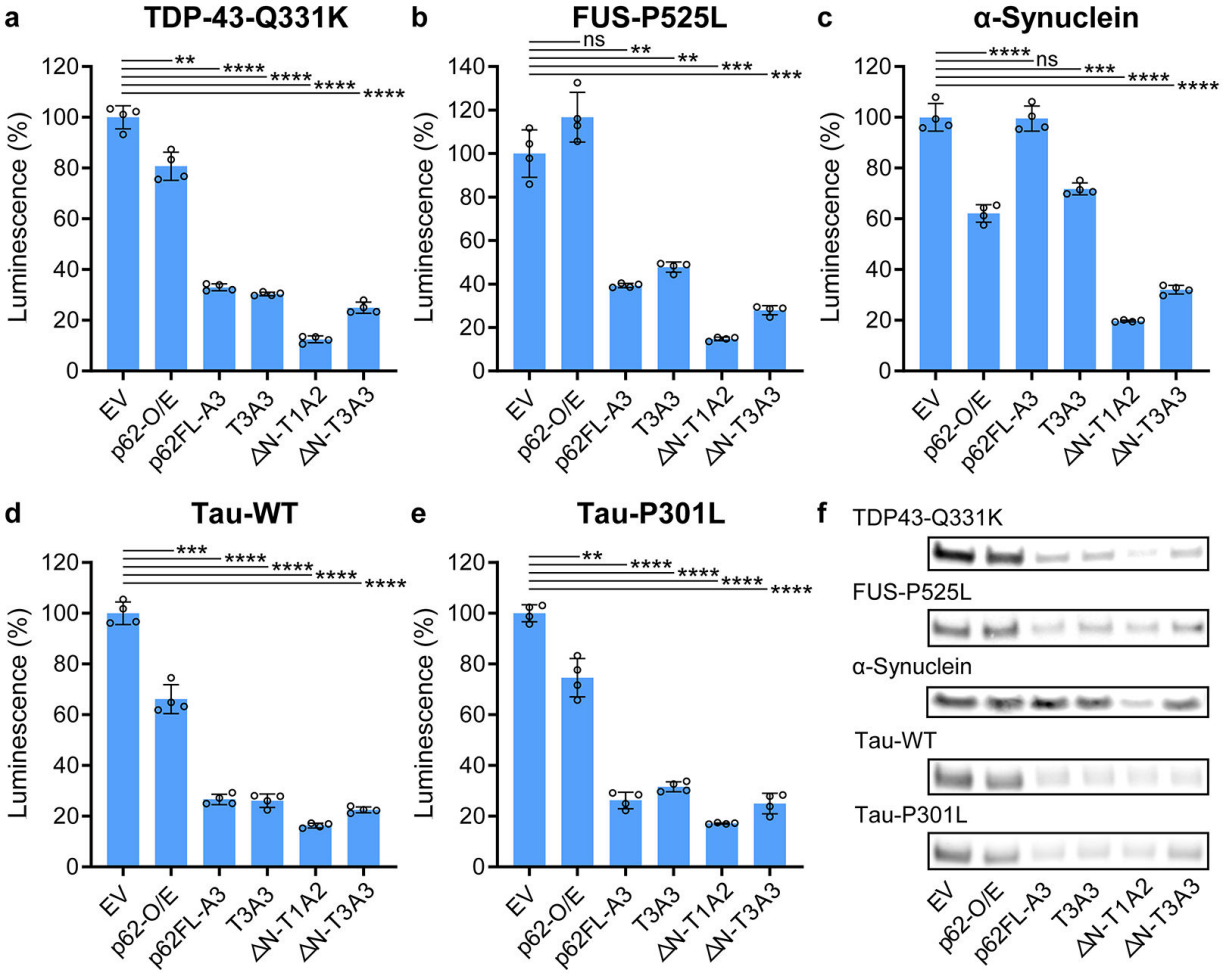
Targeted degradation of various proteins and protein aggregates by representative AceTAC degraders. (a–e) HiBiT luminescence assay to quantify the degradation of the TDP-43-Q331K mutant (a), FUS-P525L mutant (b), wild-type *α*-synuclein (c), tau (d), and tau-P301L mutant (e) by representative AceTAC degraders. Error bars represent standard deviations of *N* = 4. Statistical analyses are performed using two-tailed Student’s *t* test: **, *p* < 0.01; ***, *p* < 0.001; ****, *p* < 0.0001; ns, no significance. (f) Western blots of protein targets in the presence of representative AceTAC degraders. Western blot experiments were independent of the HiBiT luminescence assays.

After engineering the AceTAC degraders with HTT-103Q as the model target, we tested selected AceTAC degraders against representative aggregation-prone proteins. These disease-relevant targets included wild-type *α*-synuclein and tau as well as mutants of TDP-43, fused-in-sarcoma (FUS), and tau. We replaced HTT-103Q with the protein of interest in the target-encoding plasmid, keeping the N-terminal HiBiT tag and the C-terminal ALFA-tag. For these five different protein targets in U2OS cells, ΔN-T1A2 induced at least 80% degradation when compared to the EV group (Figure 2a-e). Consistent with HTT-103Q, the third generation of AceTAC degraders resulted in the highest targeted degradation performance (Figure S5). Meanwhile, in the absence of AceTAC degraders, overexpression of p62 again led to modest degradation (<35%) of the targeted protein through enhancement of autophagy. We also confirmed the degradation results of these protein targets through Western blots, which agreed with the HiBiT luminescence assays (Figure 2f and Figure S6). The successful targeted degradation of several aggregation-prone proteins demonstrated the broad applicability of the AceTAC degraders.

### Selective Degradation of Mitochondria Is Tunable through the AceTAC Degradation System

**Figure 3.**
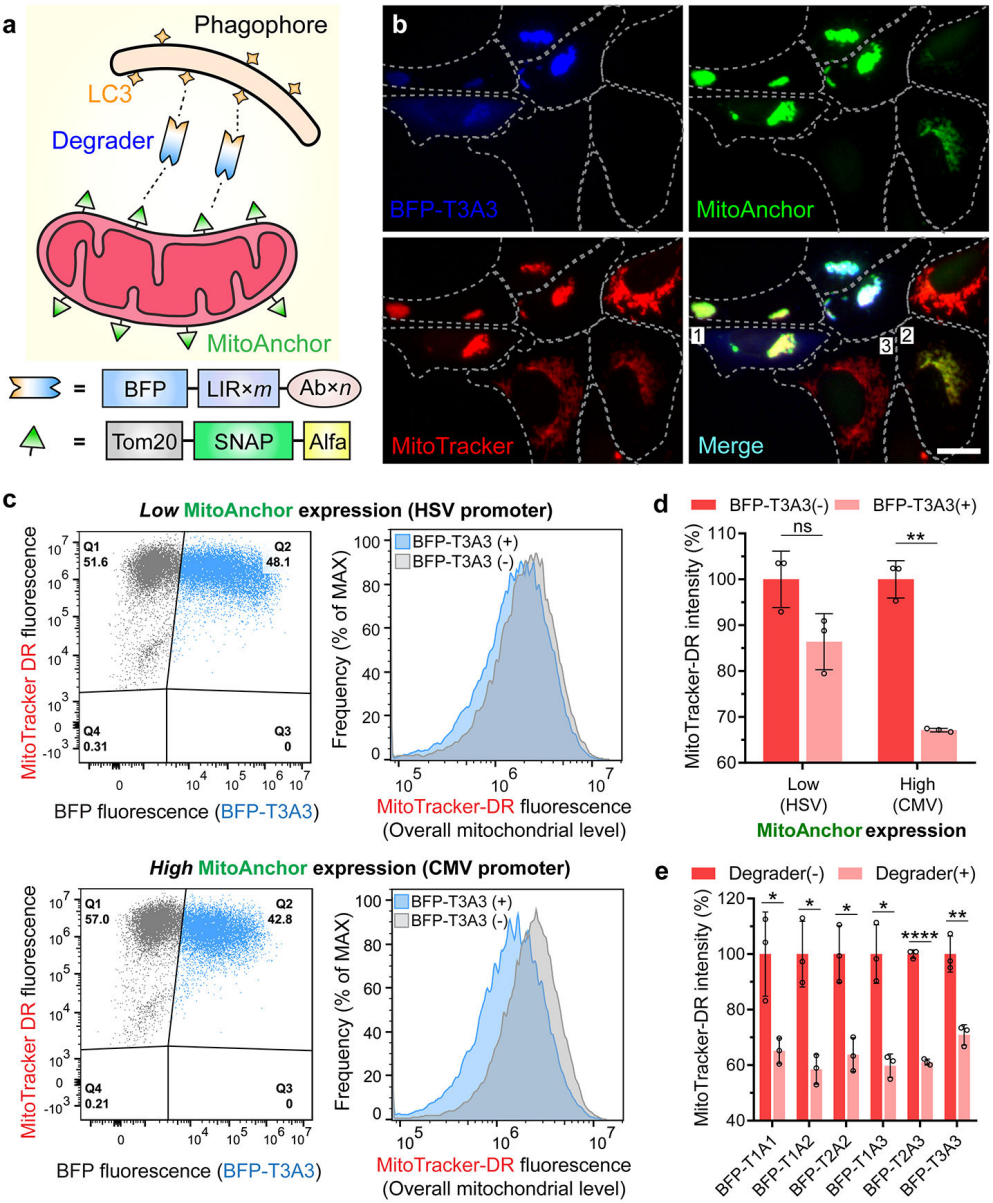
AceTAC degradation system induced tunable targeted degradation of mitochondria. (a) Schematic illustration of the AceTAC system for targeted mitochondrial degradation. (b) Representative images for the cellular localization of BFP-T3A3 degrader, MitoAnchor, and MitoTracker Deep Red (MitoTracker-DR) in live U2OS cells. Three representative cells were labeled: cell 1, co-presence of BFP-T3A3 degrader and the MitoAnchor led to the aggregation of mitochondria; cell 2, the MitoAnchor did not induce the mitochondrial aggregation in the absence of BFP-T3A3 degrader; cell 3, a control cell showed regular tubular mitochondrial network when neither BFP-T3A3 nor MitoAnchor were transfected. Scale bar: 20 *μ*m. (c) Flow cytometry dot plot analyses (left) for the BFP-T3A3 degrader intensity and the MitoTracker-DR intensity in U2OS cells. The top left quadrant of the BFP-T3A3(−) population (Q1) and the top right quadrant of the BFP-T3A3(+) population (Q2) were plotted for their corresponding histogram analyses for the MitoTracker-DR intensity (right). (d) Effect of MitoAnchor expression on the reduction of MitoTracker-DR intensity from the BFP-T3A3(−) population (Q1) to the BFP-T3A3(+) population (Q2). Error bars represent standard deviations of *N* = 3. (e) Effect of different BFP-T*m*A*n* degraders on the reduction of MitoTracker-DR intensity from the BFP(−) to the BFP(+) population. Error bars represent standard deviations of *N* = 3. Statistical analyses are performed using two-tailed Student’s *t* test” *, *p* < 0.05; **, *p* < 0.01; ***, *p* < 0.001; ****, *p* < 0.0001; ns, no significance.

After successful targeted degradation of proteins, we sought to explore whether the AceTAC system could be extended to organelles. The targeted degradation of organelles is challenging due to the large size and interfacial area of the target. Our essential design principle was to provide multiple recognition sites on the outer membrane of the organelle of interest, followed by the presence of AceTAC degraders to initiate the degradation process (Figure 3a). We designed a SNAP-tag protein-based construct to decorate the outer mitochondrial membrane (termed MitoAnchor). The MitoAnchor contained an N-terminal Tom20 peptide for mitochondrial localization^25^ and a C-terminal ALFA-tag to bind with the AceTAC degraders. Meanwhile, to enable the visualization of AceTAC degraders in live cells, we tagged a blue fluorescent protein (BFP) to the N-terminus of the ΔN-T*m*A*n* degraders, denoted as the BFP-T*m*A*n* series (Figure S7). After co-transfecting the BFP-T3A3-encoding plasmid and the MitoAnchor-encoding plasmid for 24 h, we stained the live U2OS cells with MitoTracker Deep Red FM (MitoTracker-DR) to monitor the overall cellular mitochondrial level. Different cell populations were presented due to the nature of the transient expression. When both the BFP-T3A3 degrader and the MitoAnchor were present, the cells displayed an aggregated morphology of the mitochondrial network (Figure 3b, cell 1). In contrast, in cells where BFP-T3A3 was not expressed, mitochondria showed the regular tubular network (Figure 3b, cells 2 and 3). Similar results were observed in the co-presence of BFP-T1A1 and MitoAnchor (Figure S8). Such aggregation behavior resembled the process of mitochondrial autophagy (i.e., mitophagy),^26^ consistent with the initiation of selective mitochondrial degradation through our AceTAC system.

Next, we quantified the reduction of the mitochondrial contents by AceTAC-induced mitochondrial degradation through flow cytometry. To tune the level of MitoAnchor on the outer mitochondrial membrane, we engineered the MitoAnchor construct downstream of either the strong cytomegalovirus (CMV) promoter or the weak herpes simplex virus-thymidine kinase (HSV) promoter (Figure S9).^27^ After co-transfecting BFP-T3A3 and the MitoAnchor for 24 h in U2OS cells, we compared the intensity of MitoTracker-DR between the BFP-T3A3(+) and the BFP-T3A3(−) populations (Figure 3c,d). When the MitoAnchor was under the CMV promoter for high-level expression, MitoTracker-DR intensity was reduced ~33% in the BFP-T3A3(+) population when compared to the BFP-T3A3(−) population. By contrast, MitoTracker-DR intensity was reduced only ~13% when the MitoAnchor was expressed under the weaker HSV promoter. Since the expression of BFP-T3A3 was kept constant under the CMV promoter, we conclude that the higher number of recognition sites on the targeted organelles led to the increased degradation by the AceTAC degraders. Apart from the tunability offered by the MitoAnchor expression, we also tested the BFP-T*m*A*n* degraders for the targeted degradation of mitochondria. Under the high-level expression of MitoAnchor (CMV promoter) in U2OS cells, all six BFP-T*m*A*n* degraders induced at least 30% reduction of the cellular MitoTracker-DR intensity, with both BFP-T1A2 and BFP-T2A2 showing ~40% reduction (Figure 3e). Viability assays using alamarBlue indicated that the mitochondrial loss was not caused by cytotoxicity (Figure S10a). Moreover, when the TP53INP2-LIR motif was removed from the BFP-T1A1 degrader (i.e., BFP-A1 construct), the co-presence of BFP-A1 and MitoAnchor led to negligible degradation of mitochondria (Figure S11a). These BFP-based control constructs (BFP-A_Ctrl_, BFP-T1A_Ctrl_, and BFP-A1) were either unable to colocalize with the MitoAnchor or failed to induce the aggregation of mitochondria (Figure S12). Together, these results confirmed the feasibility and tunability of targeted mitochondrial degradation through AceTAC degradation systems.

### The AceTAC Degradation System Is Applicable to the Degradation of Different Organelles

**Figure 4.**
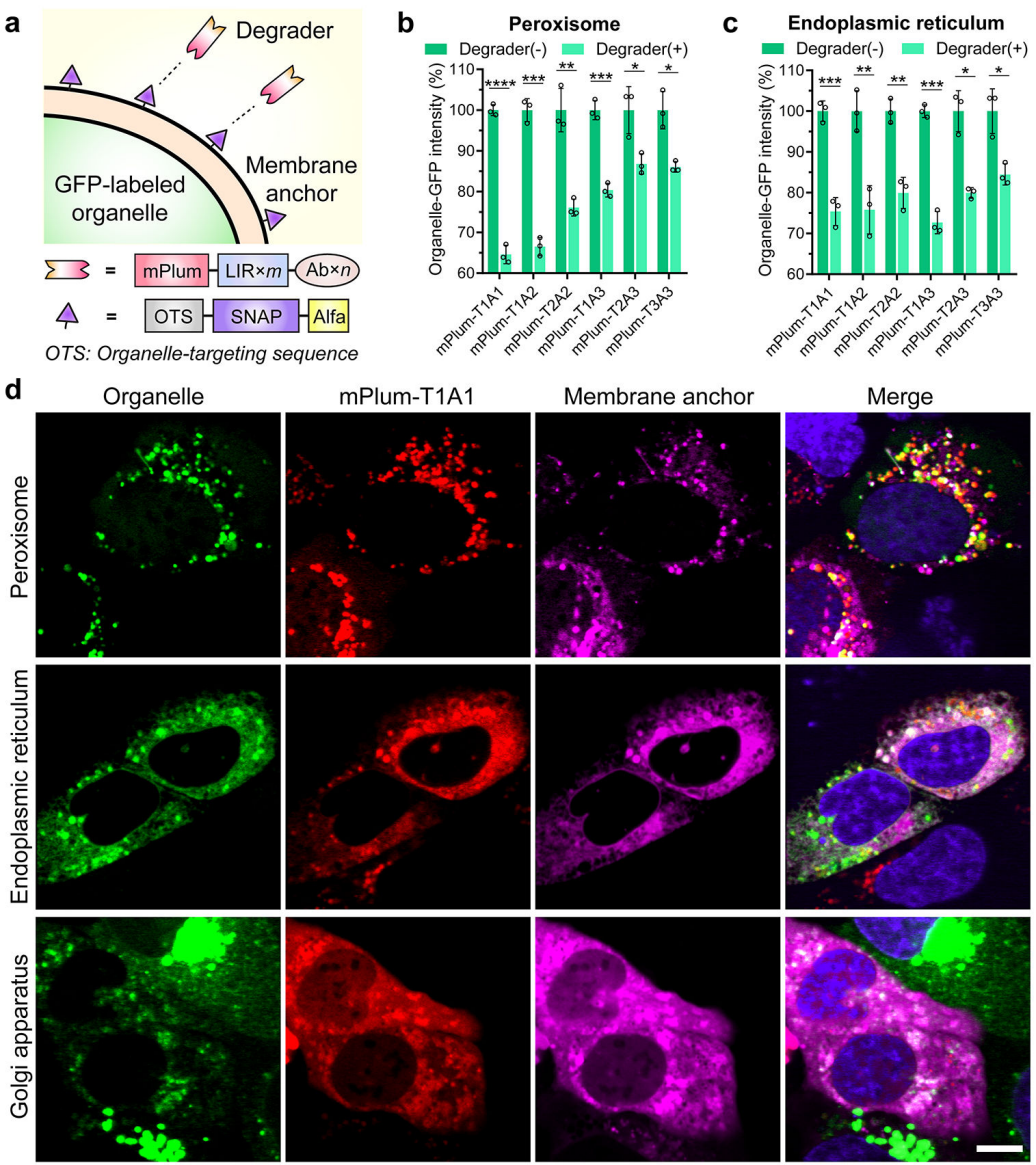
AceTAC degradation system induced the targeted degradation of peroxisomes and endoplasmic reticulum (ER). (a) Schematic illustration of the AceTAC degradation system design for GFP-labeled organelles. (b, c) Targeted degradation of GFP-labeled peroxisomes (b) and GFP-labeled ER (c) by the mPlum-T*m*A*n* degraders in U2OS cells measured by flow cytometry. Error bars represent standard deviations of *N* = 3: *, *p* < 0.05; **, *p* < 0.01; ***, *p* < 0.001; ****, *p* < 0.0001. (d) Representative images for the cellular localization of mPlum-T1A1 degrader, GFP-labeled organelles (including peroxisome, ER, and Golgi apparatus), and the corresponding membrane-anchor in live U2OS cells. The colocalized spots of these three channels were shown as white puncta in the merged channel. The merged channel also includes the nuclear staining by Hoechst 33342 (shown in blue). Scale bar, 10 *μ*m.

We envisioned that the AceTAC degradation system could be expanded to target diverse organelles in mammalian cells. Therefore, we designed a series of membrane-anchor constructs by replacing the mitochondrial localization sequence in the MitoAnchor with a targeting sequence for the organelle of interest (Figure 4a). Unlike mitochondria, most other organelles do not have a well-developed library of small-molecule dyes for specific staining and quantification. Therefore, we utilized a baculovirus-transduction platform (the BacMam system^28^) to generate cells with green fluorescence protein (GFP) labeled on the organelle of interest, taking advantage of the GFP fluorescence to quantify the level of the targeted organelle. Because GFP was not the direct target of the AceTAC degraders, the level of GFP reasonably represented the level of the labeled organelle. To avoid spectral overlap, we also replaced BFP with the mPlum fluorescent protein, generating a new series of fluorescent AceTAC degraders that were denoted as the mPlum-T*m*A*n* series.

We selected the peroxisome, ER, and Golgi apparatus as the targeted organelles for degradation. For the membrane-anchor constructs, we employed the N-terminal residues of peroxisomal biogenesis factor-3 (PEX),^29^ cytochrome P450 (CYP450),^30^ and nitric oxide synthase 3 (NOS3)^31^ as the localization tag for peroxisome, ER, and Golgi, respectively. To evaluate the targeted degradation of each organelle, we first obtained U2OS cells with GFP labeled on the targeted organelles through the BacMam system. Next, after co-transfecting the corresponding membrane-anchor construct and the mPlum-T*m*A*n*-degrader construct, we compared the organelle-GFP intensity between the mPlum-T*m*A*n*(+) and the mPlum-T*m*A*n*(−) cell populations through flow cytometry. For the targeted degradation of peroxisome, ~35% peroxisomal GFP signal reduction was achieved using the mPlum-T1A1 degrader (Figure 4b and Figures S10b, S11b, and S13a,b). For ER degradation, ~ 25% ER-GFP reduction was achieved by three mPlum-based degraders (Figure 4c and Figures S10c, S11c, and S13c,d). The rest of the degraders showed some ability to reduce both peroxisomal-GFP and ER-GFP. However, the Golgi-GFP intensity change was negligible in the presence of all AceTAC degraders (Figures S13e,f and S14).

We assessed the cellular localization of the AceTAC degradation system for targeted organelle degradation using fluorescence microscopy. For all three tested organelles, the localization of the mPlum-T1A1 degrader varied by the distribution of the specific membrane-anchor that was co-transfected (Figure 4d). This again indicated that the mPlum-T1A1 degrader bound with the C-terminal ALFA tag on the membrane anchor construct, while the localization of the membrane anchor depended on its N-terminal targeting sequence. The colocalization between the membrane-anchor/mPlum-T1A1 pair and the organelle-GFP was observed for peroxisome, ER, and Golgi. However, unlike the AceTAC-induced mitochondrial degradation, the colocalization did not appear as distinct cellular aggregates for these three organelles, presumably due to the unique nature of each organelle (e.g., organelle size, homogeneity) during degradation.^1,32-34^ Particularly regarding the Golgi apparatus, although negligible degradation was observed from flow cytometry analysis, the live cell imaging demonstrated a clear reduction in the intensity of the Golgi juxtanuclear structure in the presence of the Golgi membrane-anchor and the mPlum-T1A1 degrader, indicating that Golgi morphology was disrupted (Figure 4d). Similar to the AceTAC-induced mitochondrial degradation (Figure S12), the mPlum-based control constructs (mPlum-A_Ctrl_, mPlum-T1A_Ctrl_, and mPlum-A1) did not efficiently alter the morphology of these organelles (Figures S15, S16, and S17). Overall, the AceTAC system revealed a tunable and promising strategy for targeted organelle degradation.

### The Targeted Degradation by AceTAC Degraders Is Facilitated by Autophagy

**Figure 5.**
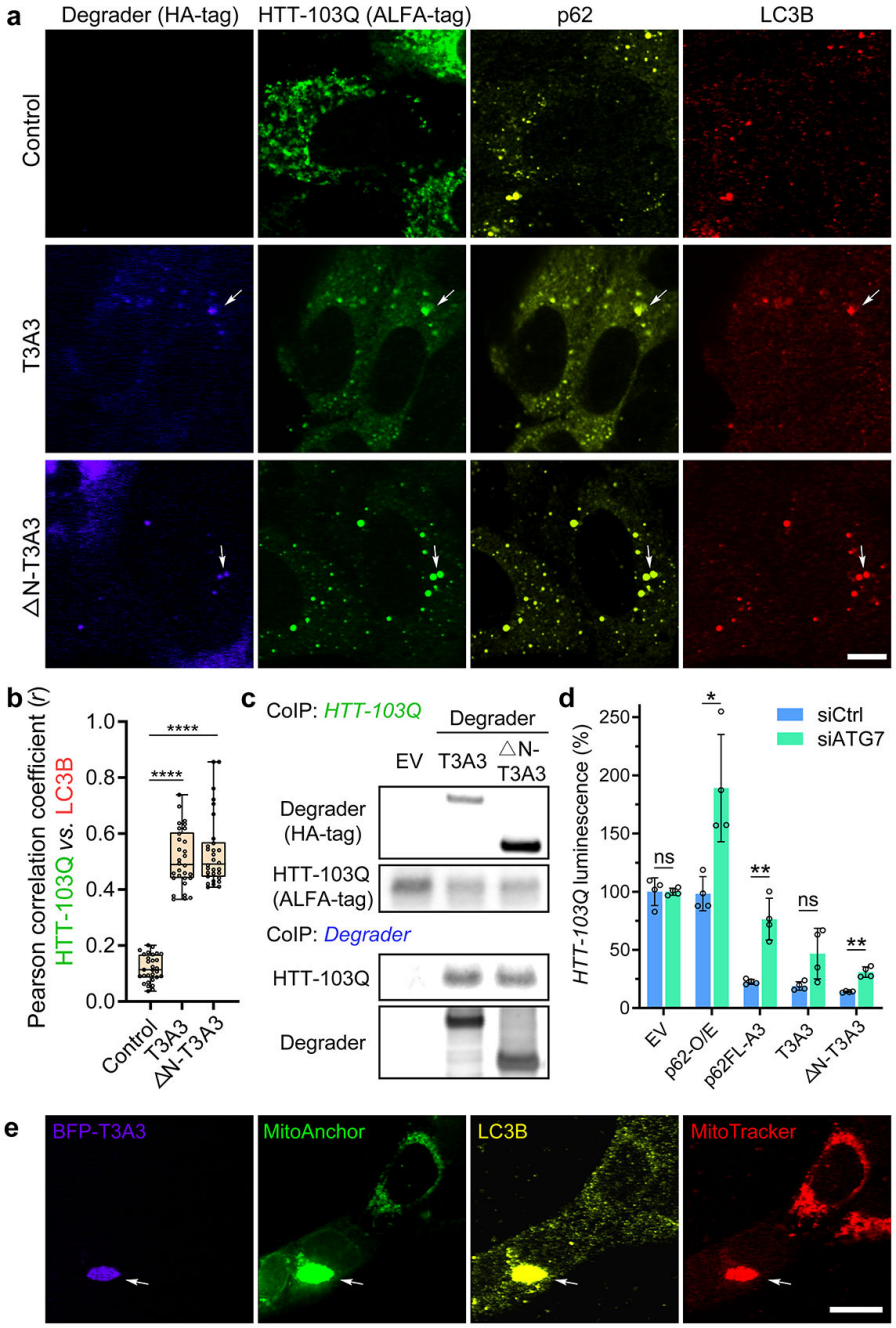
The targeted degradation induced by AceTAC degraders utilized the autophagy process. (a) Representative images for the cellular localization of the AceTAC degraders (T3A3 and ΔN-T3A3), HTT-103Q target, endogenous p62, and endogenous LC3B in U2OS cells. The plasmids encoding the AceTAC degraders and HTT-103Q were transfected to U2OS cells for 24 h. Scale bar, 10 *μ*m. (b) Pearson correlation coefficient (*r*) between HTT-103Q and LC3B from the IF image analysis of 30 representative cells in each group. Statistical analyses are performed using two-tailed Student’s *t* test: ****, *p* < 0.0001. (c) Co-immunoprecipitation (co-IP) of HTT-103Q from U2OS cells after transfection of plasmids that encode HTT-103Q and AceTAC degraders. HTT-103Q was immunoprecipitated via the ALFA-tag; HTT-103Q-bound protein complexes were then captured from the lysates and blotted for the ALFA-tag (HTT-103Q) and HA-tag (AceTac degrader). (d) HiBiT luminescence assay to evaluate the effect of ATG7-knockdown on the targeted HTT-103Q degradation by representative AceTAC degraders in U2OS cells. Error bars represent standard deviations of *N* = 4. Statistical analyses are performed using two-tailed Student’s *t* test: *, *p* < 0.05; **, *p* < 0.01; ns, no significance. (e) Representative images for the cellular localization of the BFP-T3A3 degrader, MitoAnchor, LC3B, and MitoTracker-DR in live U2OS cells. Colocalization occurs at a large cytoplasmic aggregate. Scale bar, 20 *μ*m.

We assessed whether the targeted degradation by AceTAC degraders utilized the autophagy process. First, we conducted immunofluorescence (IF) to verify the cellular localization of the degrader and protein target, along with endogenous p62 and LC3B, since both p62 and LC3B have been used as common biomarkers for autophagosomes. Two representative degraders, T3A3 and ΔN-T3A3, were compared with the control group that was only transfected with HTT-103Q in U2OS cells. In the absence of the degrader, the HTT-103Q spread over the cytoplasm as amorphous aggregates, while it minimally colocalized with p62 and LC3B. Comparatively, in the presence of either T3A3 or ΔN-T3A3 degrader, the morphology of HTT-103 changed to puncta within the cytoplasm (Figure 5a and Figures S18 and S19). Most importantly, the introduction of the AceTAC degraders led to the colocalization among the degrader itself, the degradation target (HTT-103Q), endogenous p62, and endogenous LC3B within the puncta (Figure 5b). Constructs based on control nanobodies (A_Ctrl_ and ΔN-T1A_Ctrl_) failed to induce the puncta formation of HTT-103Q (Figure S20). Interestingly, the removal of the LIR-motif in representative AceTAC degraders still led to puncta formation and colocalization signature similar to those of the complete AceTAC degraders (Figure S21). Considering the efficient degradation capability of these ΔLIR constructs (Figure 1d), the induced aggregation step was further validated to be essential in AceTAC-induced protein degradation. Moreover, HTT-103Q coimmunoprecipitated with the AceTAC degrader (i.e., T3A3 or ΔN-T3A3) from U2OS cell lysates (Figure 5c and Figure S22), demonstrating the binding between the target and the AceTAC degraders.

We also validated the mechanism of action for the AceTAC-induced mitophagy through IF. The colocalization among the BFP-T3A3 degrader, the MitoAnchor, LC3B, and the MitoTracker-DR occurred as an aggregated cytoplasmic structure (Figure 5e), consistent with colocalization observed during the targeted degradation of HTT-103Q. Next, we compared the AceTAC-induced mitophagy with CCCP, a small-molecule inducer of mitophagy^35^ (Figure S23). We conducted Western blotting of 16 proteins that were relevant to autophagy and mitophagy, including selected autophagy receptors, ATG proteins, and kinases (Figure S24). The AceTAC degradation system decreased the level of OPTN, an autophagy receptor that was found to be involved during the mitophagy of damaged mitochondria.^36^ Although both the AceTAC- and CCCP-induced mitophagy increased the LC3-II/I ratio, a different protein expression pattern was observed. For example, p62 was significantly increased in the AceTAC degradation system while it was decreased by the CCCP-treatment; the PINK1 level was boosted by CCCP-treatment as previously reported,^37^ whereas minimal change resulted from the AceTAC system. The reason for these differences will be further investigated.

We further investigated the mechanism of action for the AceTAC degradation system through several orthogonal validations. ATG7 is required for the formation of LC3-II,^38^ the lipidated LC3 that anchors to the autophagosomal membrane. ATG7-knockdown (ATG7-KD) therefore inhibits the autophagic flux. We achieved more than 90% knockdown of ATG7 using siRNA (Figure S25). When compared to wild-type U2OS cells, the degradation of HTT-103Q by the AceTAC degraders was suppressed in the cells with a reduced ATG7-level (Figure 5d). Moreover, ATG5-KD^39^ also decreased the degradation efficiency of AceTAC degraders against HTT-103Q (Figure S26). The targeted degradation efficiency of AceTAC degraders also slightly increased under autophagy-inducing conditions (Torin1-treatment^40^ and serum-starvation^41^) (Figure S27). The presence of the TP53INP2-LIR motif in the degrader not only enabled the binding between the degrader and the autophagosomal membrane (Figure 5a-c) but also increased autophagic activity even in the absence of target-specific antibody based on immunoblotting for LC3II/I ratio and p62 levels (Figure S28). This result is reasonable as the cytoplasmic presence of truncated TP53INP2 has been shown to trigger the LC3-II production and autophagosome formation.^42^ Furthermore, we assessed if the ubiquitin–proteasome system (UPS) contributed to the AceTAC degraders by treatment of carfilzomib, an irreversible proteasome inhibitor.^43^ Upon carfilzomib-treatment, no significant difference was observed between the degraders and the control group for HTT-103Q degradation (Figure S29). However, the slight differences among the three generations of AceTAC degraders suggested that we should continue to evaluate the role of UPS in later generations of the AceTAC degradation system.

Last, to our surprise, the targeted degradation efficiency of HTT-103Q by AceTAC degraders was improved in p62- knockout (p62KO) U2OS cells compared to wild-type U2OS cells (Figure S30). Previous studies have demonstrated that the depletion of p62 led to increased cytoplasmic HTT-polyQ proteins.^44^ As the autophagy machinery functions in the cytoplasm, the increased degradation efficiency of HTT-103Q by AceTAC degraders in p62KO cells might be attributed to the enhanced availability of protein targets (i.e., HTT-103Q) in the cytoplasm. All of these results together strongly supported that the targeted degradation by AceTAC degraders was facilitated by autophagy.

## CONCLUSIONS

We have described the development of Ab-fusion (AceTAC) degraders for the targeted degradation of protein aggregates and organelles. These AceTAC degraders were inspired by the structure and function of p62, a major autophagy receptor. Through protein engineering, the optimized AceTAC degraders were simple constructs containing the LC3-interacting region of TP53INP2 and an ALFA-targeting nanobody, resulting in the efficient degradation of intracellular proteins. When recognition sites were decorated on the outer membrane of a specific organelle, the AceTAC degradation system could be systematically tuned to induce the aggregation and degradation of the targeted organelle.

Thus, the AceTAC degradation technology may be suitable for removing large intracellular contents that are processed for degradation via autophagy. While several recent reports utilized the autophagy process for targeted protein degradation, it is still challenging to develop a systematic strategy for efficient targeted organelle degradation. For example, the AUTOTAC technology utilized bifunctional small-molecules to link between the p62-ZZ domain and the target, efficiently degrading protein targets, although not exploring the organelle context,^15^ while Li and co-workers showed the degradation of fluorescently labeled peroxisomes in plants.^45^ Here, we demonstrate initial inroads into targeted organelle degradation in mammalian cells, particularly for endogenous mitochondria, revealing applicable design principles for the successful degradation of peroxisomes and ER. This AceTAC degradation system is built on the interaction between an epitope tag and its corresponding single-domain antibody fragment. This use of antigen–antibody recognition is highly general, but the method can also be extended to other protein–protein interaction pairs. In addition to existing antibodies, discovery methods such as phage display^46^ will further extend the applicability of the technology, taking advantage of disease-relevant biomarkers to improve the efficiency and specificity to endogenous protein targets. Overall, the precision of the antibody-based AceTAC system possesses potential as a tool to study protein aggregates and organelle degradation as well as drug discovery platforms. The applicability of the AceTAC degradation system can be further broadened by the maturation of the protein delivery methods.

## Supplementary Material

Supporting Information

2
