# Supplementary material for: Autophagy Receptor-Inspired Antibody-Fusion Proteins for Targeted Intracellular Degradation
Source: J Am Chem Soc. Author manuscript; Available in PMC 2024 Feb 5. (PMC10636752; doi:10.1021/jacs.3c05199)
Supplement: Supporting Information [file NIHMS1934253-supplement-Supporting_Information.pdf]

Supporting Information

Autophagy receptor-inspired antibody-fusion proteins for targeted intracellular degradation

Ziwen Jiang,[*,†,‡] Yu-Hsuan Kuo,*[†,‡] Michelle R. Arkin*[*,†,‡]

[†]Department of Pharmaceutical Chemistry, [‡]Small Molecule Discovery Center, University of California, San Francisco, CA 94158, USA.

[*]Emails: ziwen@outlook.com (Z.J.); michelle.arkin@ucsf.edu (M.R.A.)

Table of Contents

1. Supplementary figures

Figure S1. Western blots of the AceTAC degrader expression in U2OS cells.

Figure S2. Targeted degradation of HTT-103Q by the deletion mutants of the T3A3 degrader.

Figure S3. Relative mRNA level of ALFA-tagged target in U2OS cells using the co-transfection method.

Figure S4. Targeted degradation of HTT-103Q could be modulated by doxycycline-inducible expression of AceTAC degraders.

Figure S5. Targeted degradation of various proteins and protein aggregates by the ΔN-T*m*A*n* degraders.

Figure S6. Western blots of protein targets and degraders in U2OS cells.

Figure S7. Western blots of the BFP-T*m*A*n* degrader expression in U2OS cells.

Figure S8. Representative images for the cellular localization of the BFP-T1A1, MitoAnchor, and MitoTracker staining in U2OS cells.

Figure S9. Flow cytometry histogram analyses for the expression of MitoAnchor-deGFP under different promoters in U2OS cells.

Figure S10. Cell viability assay evaluating the degrader and membrane-anchor in U2OS cells.

Figure S11. Effect of control constructs on mitochondria, peroxisome, and ER in U2OS cells.

Figure S12. Representative images for the cellular localization of the BFP-based constructs, MitoAnchor and MitoTracker staining in U2OS cells.

Figure S13. Flow cytometry analyses for the mPlum-T1A1 degrader intensity and the organelle-GFP intensity in U2OS cells.

Figure S14. Effect of degrader constructs on Golgi apparatus in U2OS cells.

Figure S15. Representative images for the cellular localization of the GFP-labelled peroxisome, mPlum-based constructs, and Membrane anchor in U2OS cells.

Figure S16. Representative images for the cellular localization of the GFP-labelled endoplasmic reticulum, mPlum-based constructs, and Membrane anchor in U2OS cells.

Figure S17. Representative images for the cellular localization of the GFP-labelled Golgi apparatus, mPlum-based constructs, and Membrane anchor in U2OS cells.

Figure S18. Representative images for the cellular localization of representative AceTAC degraders, HTT-103Q target, endogenous p62, and endogenous LC3B in U2OS cells (greyscale individual channels and color-coded merged channel).

Figure S19. Representative images for the cellular localization of the p62-overexpression, HTT-103Q target, endogenous p62, and LC3B in U2OS cells.

Figure S20. Representative images for the cellular localization of the control constructs, HTT-103Q target, endogenous p62, and LC3B in U2OS cells.

Figure S21. Representative images for the cellular localization of HA-tagged constructs, HTT-103Q target, endogenous p62, and LC3B in U2OS cells, comparing the effect of removing the LIR motif in AceTAC degraders.

Figure S22. Workflow of the co-immunoprecipitation and western blots for U2OS cells after plasmid transfection.

Figure S23. Dose optimization for CCCP-treatment to induce mitophagy in U2OS cells.

Figure S24. Western blot analysis of AceTAC-induced mitophagy in U2OS cells.

Figure S25. Western blot analysis to validate the successful knockdown of ATG7 in U2OS cells.

Figure S26. Effect of ATG5-knockdown on the targeted degradation of HTT-103Q by AceTAC degraders in U2OS cells.

Figure S27. Effect of autophagy-modulating conditions for AceTAC degraders in U2OS cells.

Figure S28. The LIR motif of TP53INP2 in the AceTAC degrader modulated the autophagic flux.

Figure S29. Effect of a representative proteasome inhibitor (carfilzomib) for AceTAC degraders in U2OS cells.

Figure S30. Effect of p62-knockout for AceTAC degraders in U2OS cells.

2. Supplementary methods

3. Supplementary sequences

3.1. Degraders

3.2. Targets

3.3. Membrane anchors

4. References

1. Supplementary Figures

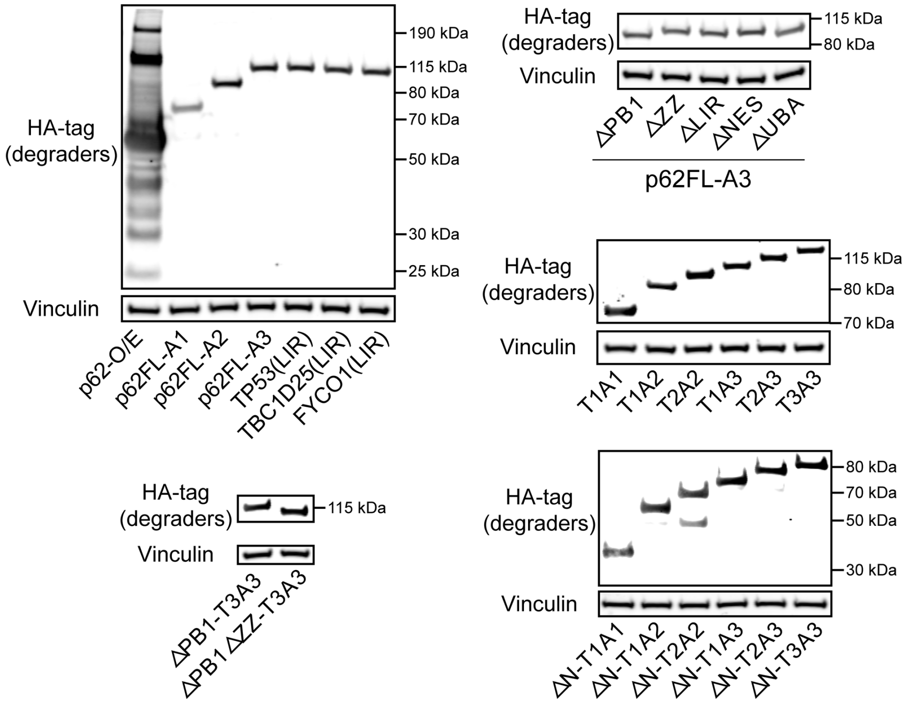

Figure S1. Western blots of the AceTAC degrader expression in U2OS cells.

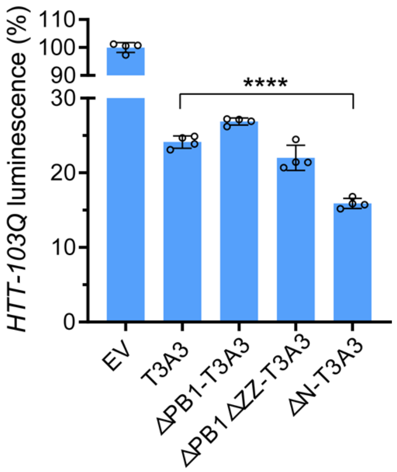

Figure S2. Targeted degradation of HTT-103Q by the deletion mutants of the T3A3 degrader. Error bars represent standard deviations of $N = 4$. Statistical analyses are performed using two-tailed Student's t test. ****, $p < 0.0001$.

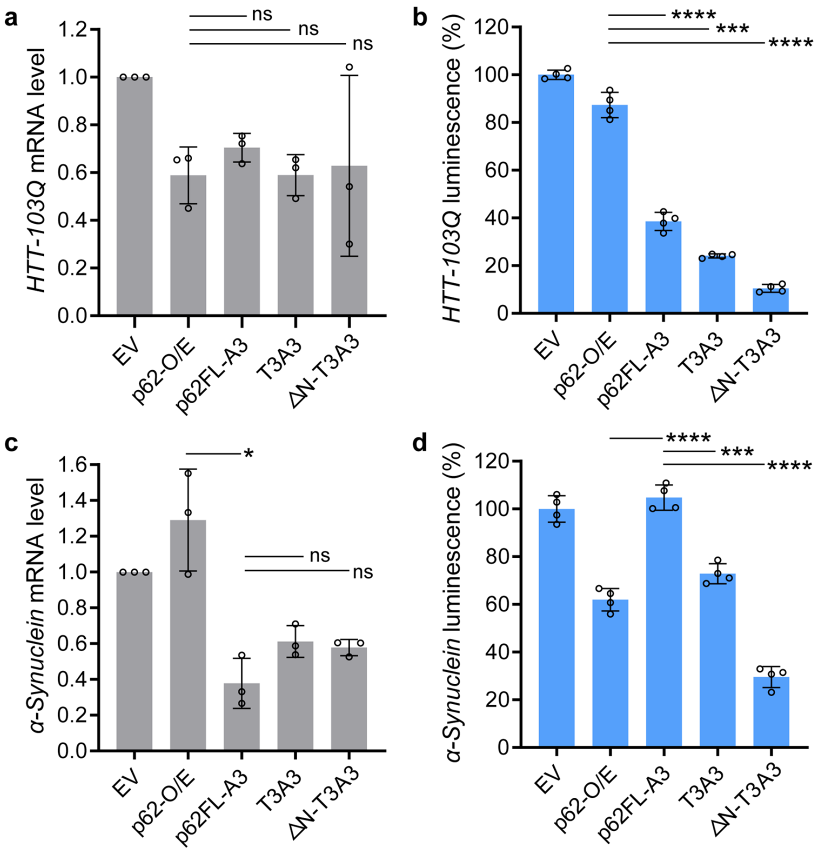

Figure S3. a, Relative mRNA level of ALFA-tagged HTT-103Q in U2OS cells after co-transfecting the HTT-103Q-encoding plasmid and the degrader-encoding/EV plasmid. The corresponding targeted HTT-103Q degradation profile was shown in (**b**). **c**, Relative mRNA level of ALFA-tagged HTT-103Q in U2OS cells after co-transfecting the HTT-103Q-encoding plasmid and the degrader-encoding/EV plasmid. The corresponding targeted HTT-103Q degradation profile was shown in (**d**). In the presence of autophagy-inducing plasmids, the mRNA level of HTT-103Q was slightly (~40%) reduced compared to mRNA in the cells with an empty vector as the second plasmid. However, the levels of HTT-103Q degradation were strikingly lower when AceTAC degraders were transfected, supporting our proposal that AceTAC-induced targeted degradation occurred at the protein level. Similarly, for ALFA-tagged α-synuclein, co-transfection with p62FL-A3 resulted in the lowest level of α-synuclein mRNA, while the α-synuclein expression was not affected. Altogether, this dataset demonstrated that the co-transfection method did not confound our assessment of targeted protein degradation. Error bars represent standard deviations of $N = 3$ (**a**,**c**) or $N = 4$ (**b**,**d**). Statistical analyses are performed using two-tailed Student's t test to compare with the group that shows the least mRNA level. *, $p < 0.05$; ***, $p < 0.001$; ****, $p < 0.0001$; ns, no significance.

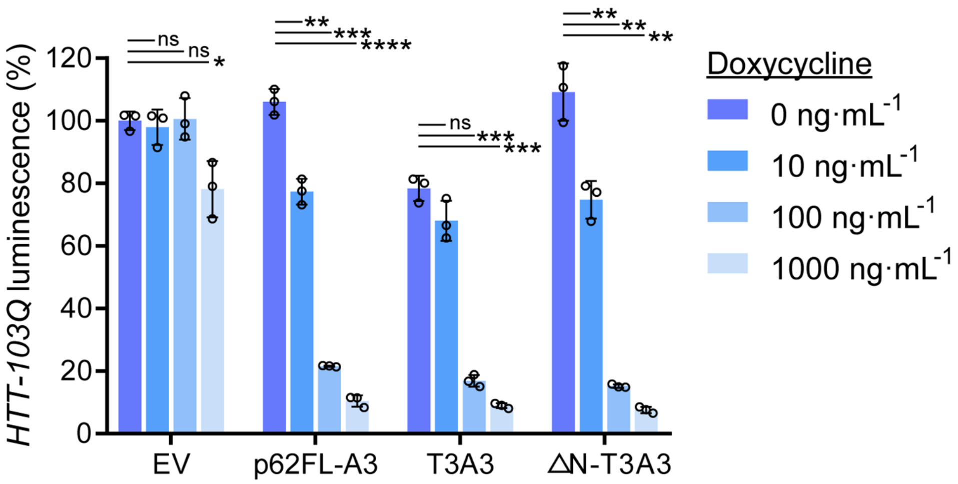

Figure S4. Targeted degradation of HTT-103Q could be modulated by doxycycline (Dox)-inducible expression of AceTAC degraders. Briefly, the HTT-103Q-encoding plasmid and Dox-inducible degrader-encoding plasmid (or EV for the control group) were co-transfected into U2OS cells for 24 hours in the presence of varied concentrations of Dox. The transfected cells were further incubated for 6 hours in fresh complete growth medium that contained the corresponding Dox concentration. Error bars represent standard deviations of $N = 3$. Statistical analyses are performed using two-tailed Student's t test. *, $p < 0.05$; **, $p < 0.01$; ***, $p < 0.001$; ****, $p < 0.0001$; ns, no significance.

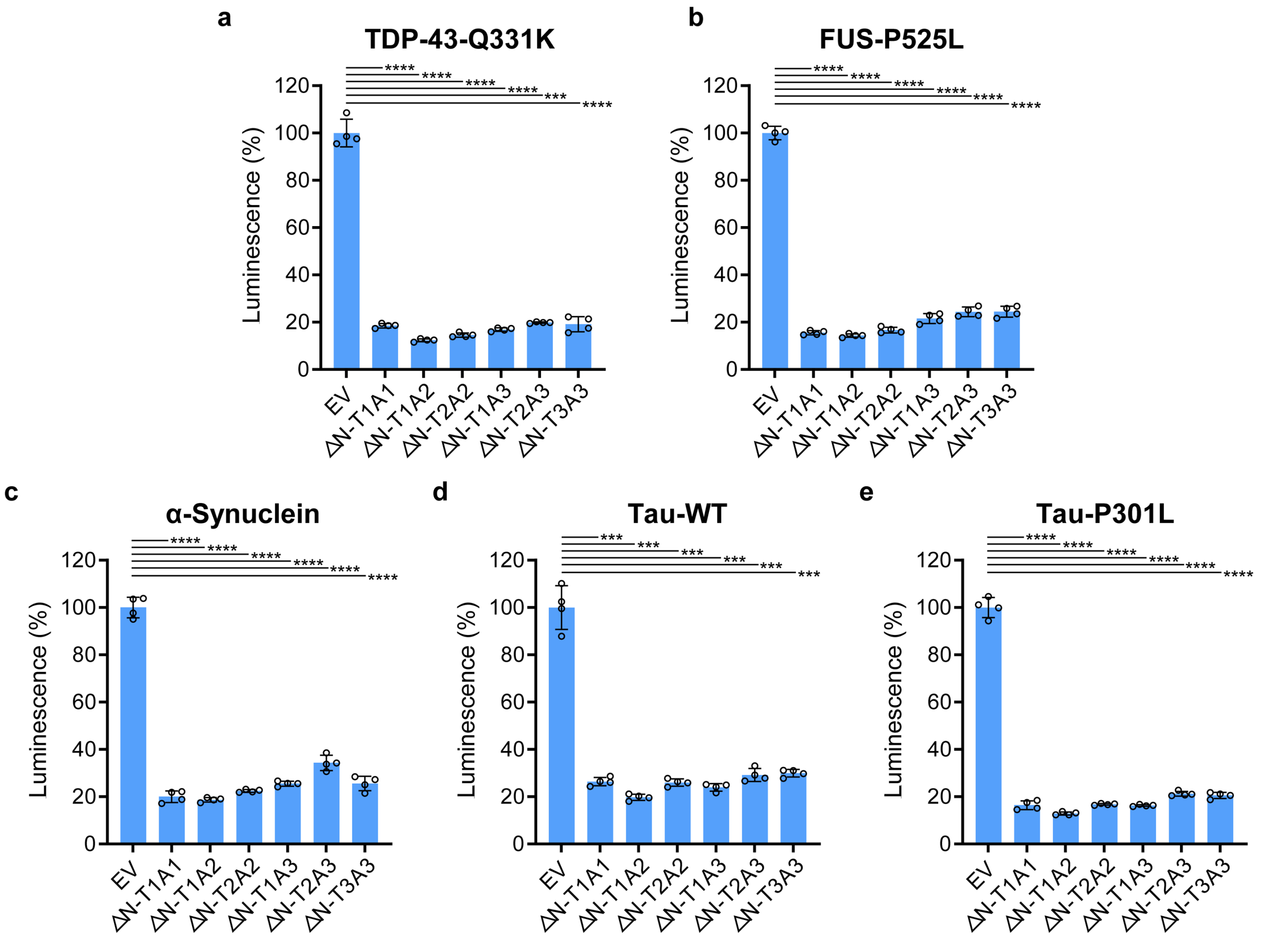

Figure S5. HiBiT luminescence assay to quantify the degradation of TDP-43-Q331K mutant (**a**), FUS-P525L mutant (**b**), wild-type α-synuclein (**c**), Tau (**d**), and Tau-P301L mutant (**e**) by the ΔN-T*m*A*n* degraders. Error bars represent standard deviations of $N = 4$. Statistical analyses are performed using two-tailed Student's t test. ***, $p < 0.001$; ****, $p < 0.0001$.

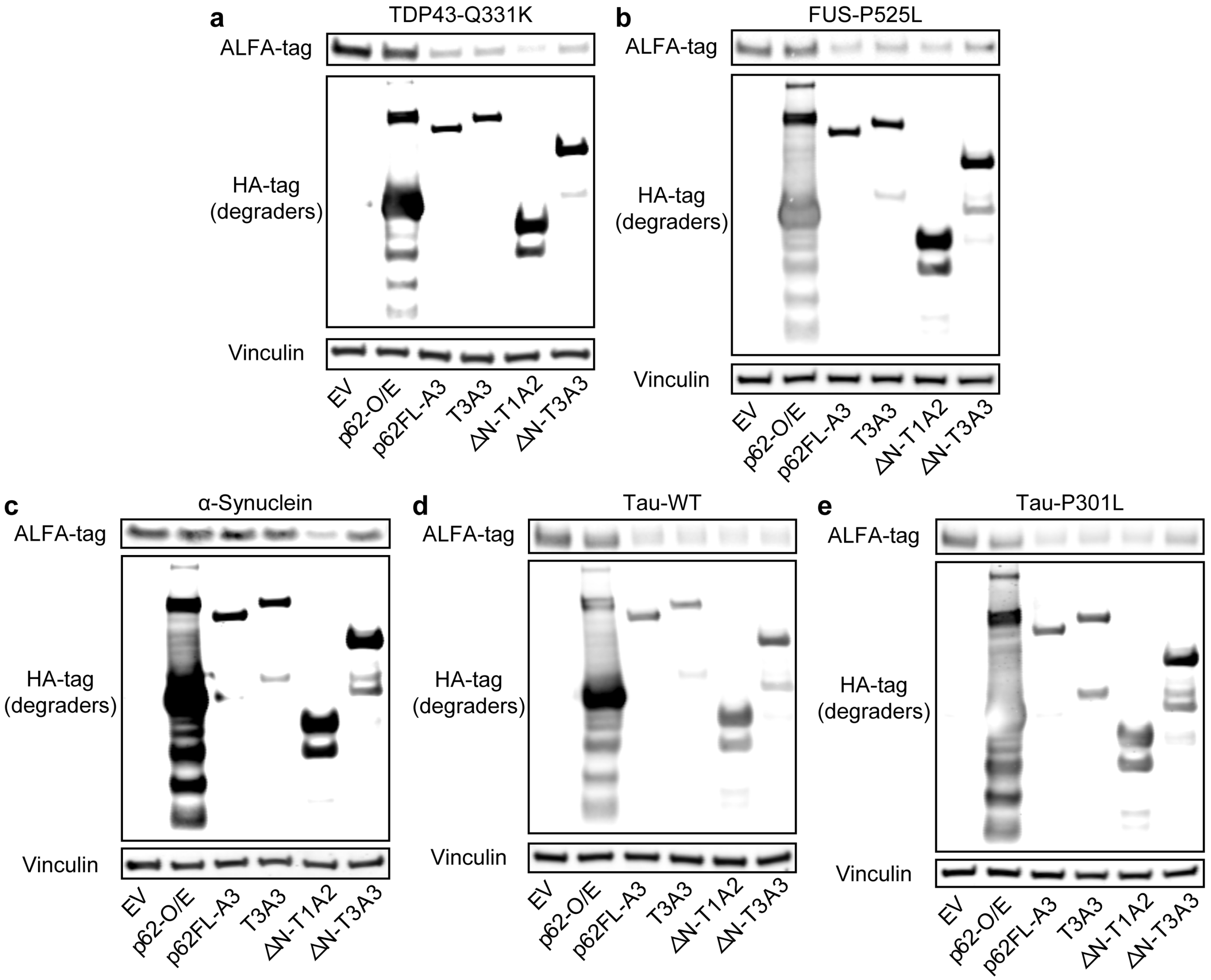

Figure S6. Western blots of protein targets (ALFA-tagged) and degraders (HA-tagged) after 24-hour plasmid transfection in U2OS cells.

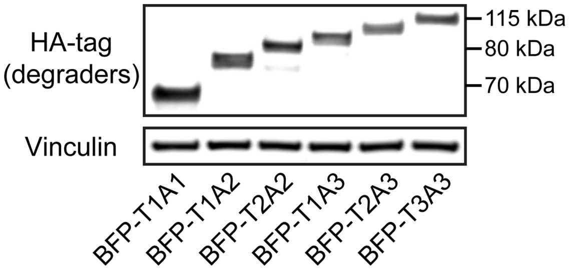

Figure S7. Western blots of the BFP-T*m*A*n* degrader expression in U2OS cells.

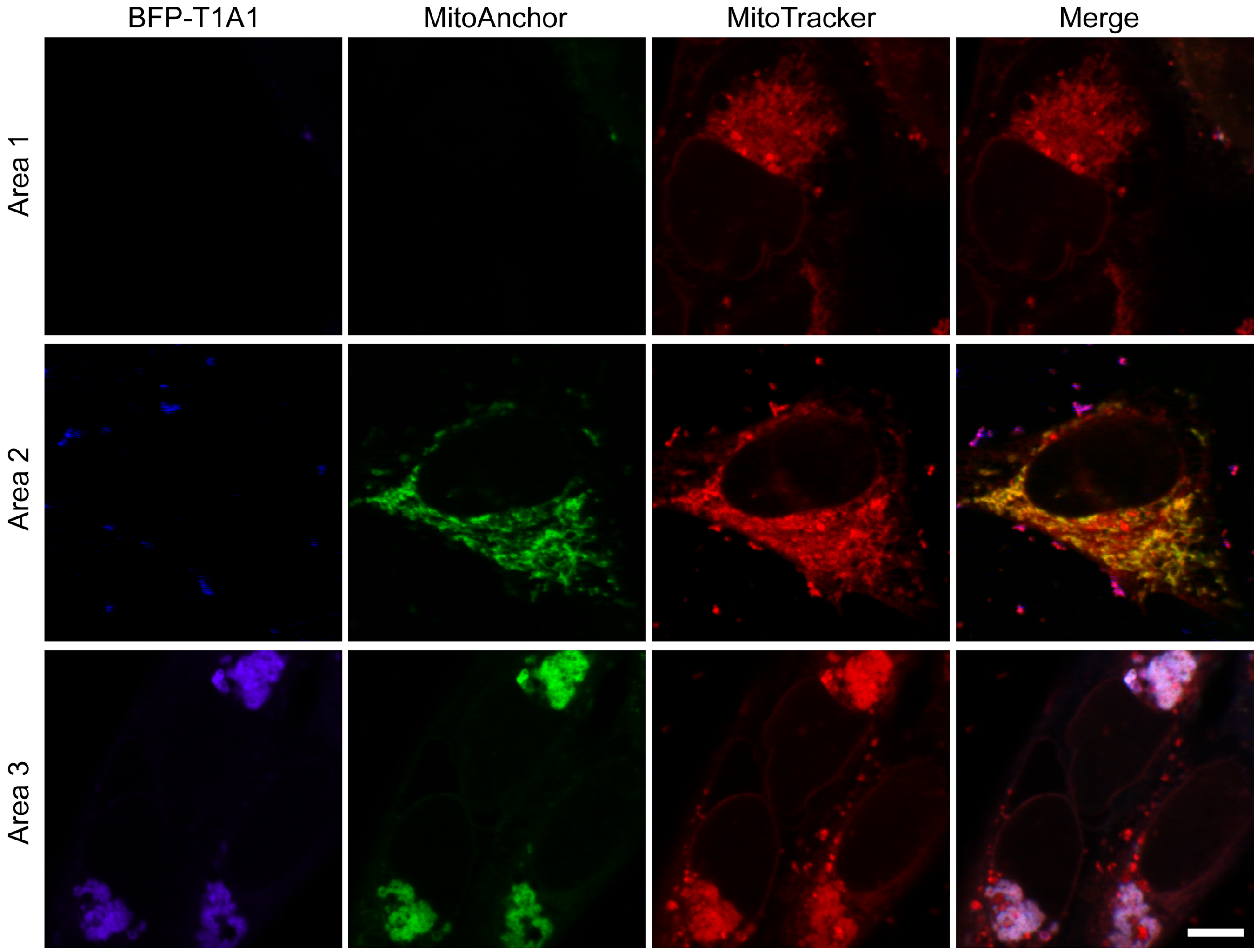

Figure S8. Representative images for the cellular localization of the BFP-T1A1, MitoAnchor (stained by SNAP-Cell Oregon Green), and MitoTracker Deep Red staining in U2OS cells. Scale bar, 10 μm.

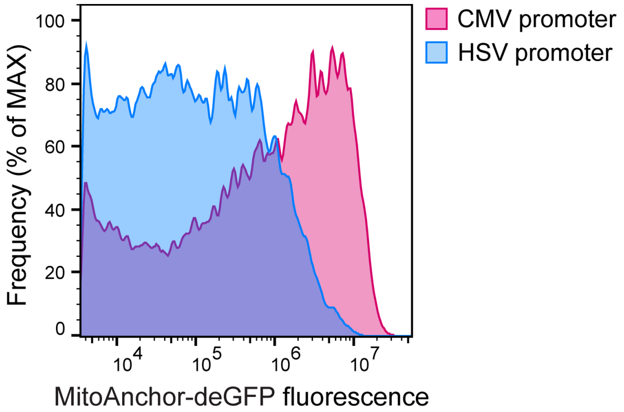

	MitoAnchor-deGFP (+) cells (%)	Median of MitoAnchor-deGFP
CMV promoter	75.5 ± 1.2	$(7.8 \pm 0.9) \times 10^5$
HSV promoter	49.7 ± 2.6	$(8.4 \pm 0.8) \times 10^4$

Figure S9. Flow cytometry histogram analyses for the expression of MitoAnchor-deGFP under different promoters in U2OS cells. The analysis was conducted after transfecting the MitoAnchor-deGFP-encoding plasmids in U2OS cells for 24 hours. deGFP is a destabilized version of GFP with a 2-hour fluorescence half-life.[SR1] Data in the table represent standard deviations of $N = 4$.

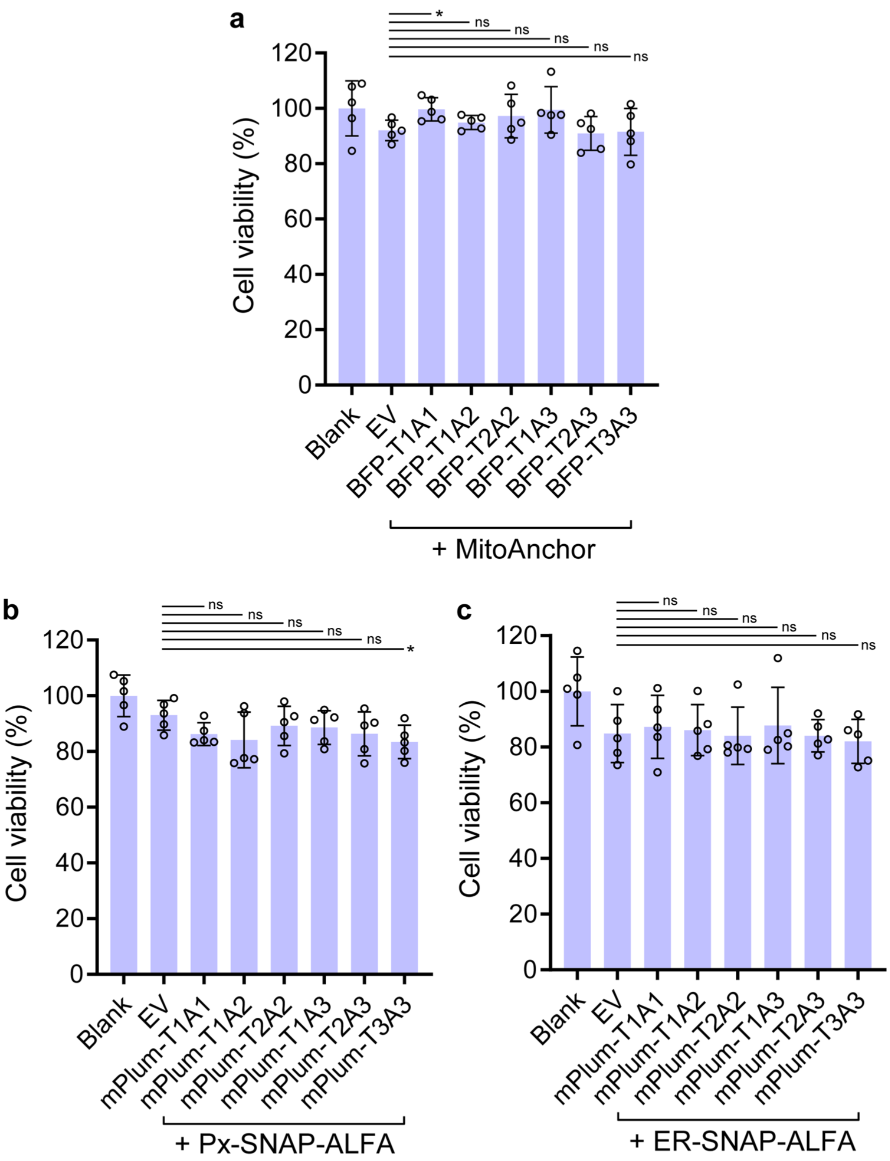

Figure S10. Cell viability (alamarBlue) assay after the co-transfection of degrader-encoding and membrane-anchor-encoding plasmids in U2OS cells for 24 hours. Cells were highly viable in the presence of both the membrane anchor and the degraders/EV. EV, empty vector. Error bars represent standard deviations of $N = 5$. Statistical analyses are performed using two-tailed Student's t test. *, $p < 0.05$; ns, no significance.

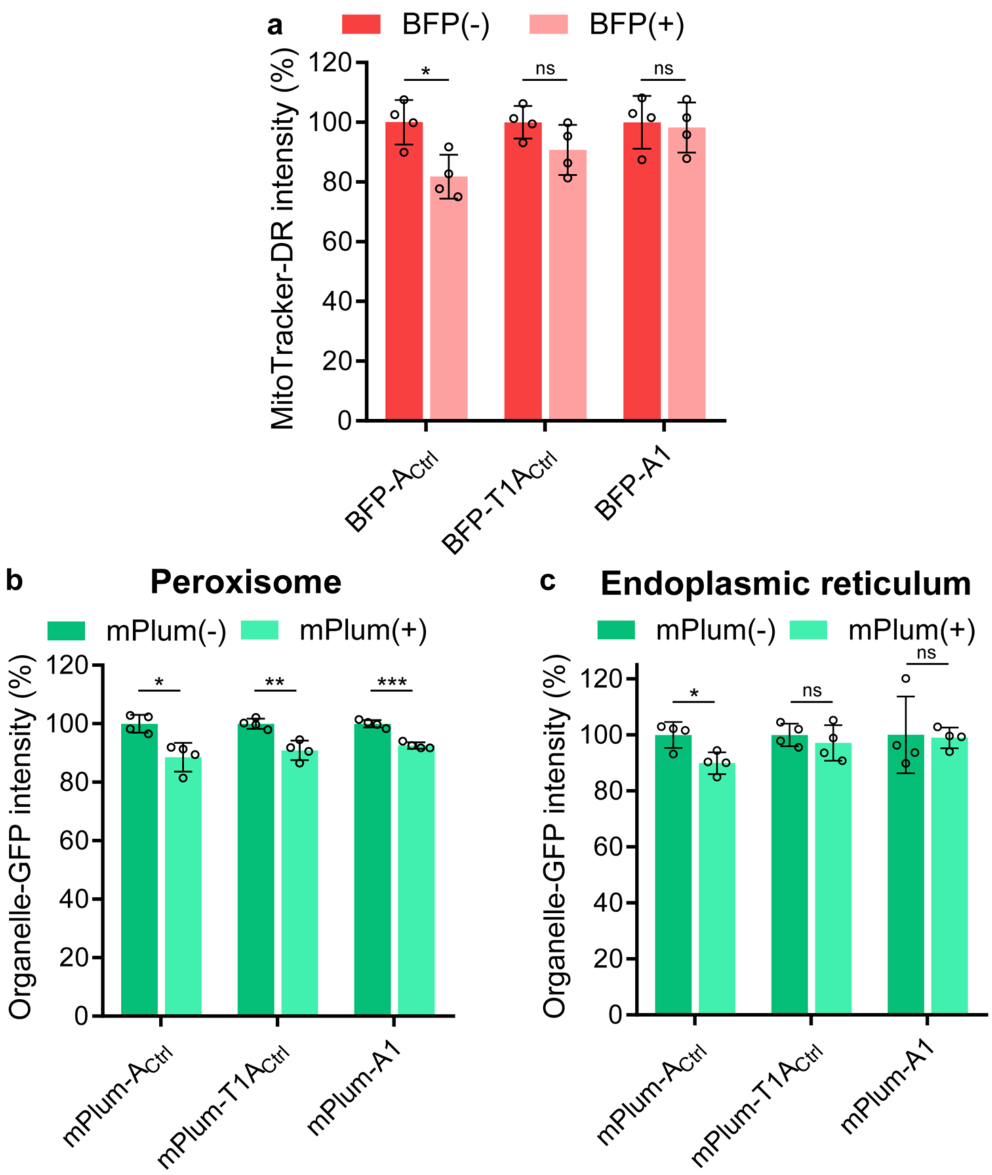

Figure S11. a, Effect of different BFP-based control constructs on the MitoTracker-DR intensity in U2OS cells. Error bars represent standard deviations of $N = 4$. **b,c**, Effect of different mPlum-based control constructs on GFP-labelled peroxisomes (**b**) and GFP-labelled ER (**c**) in U2OS cells. The quantification was conducted by flow cytometry analysis. Error bars represent standard deviations of $N = 4$. Statistical analyses are performed using two-tailed Student's t test. *, $p < 0.05$; **, $p < 0.01$; ***, $p < 0.001$; ns, no significance.

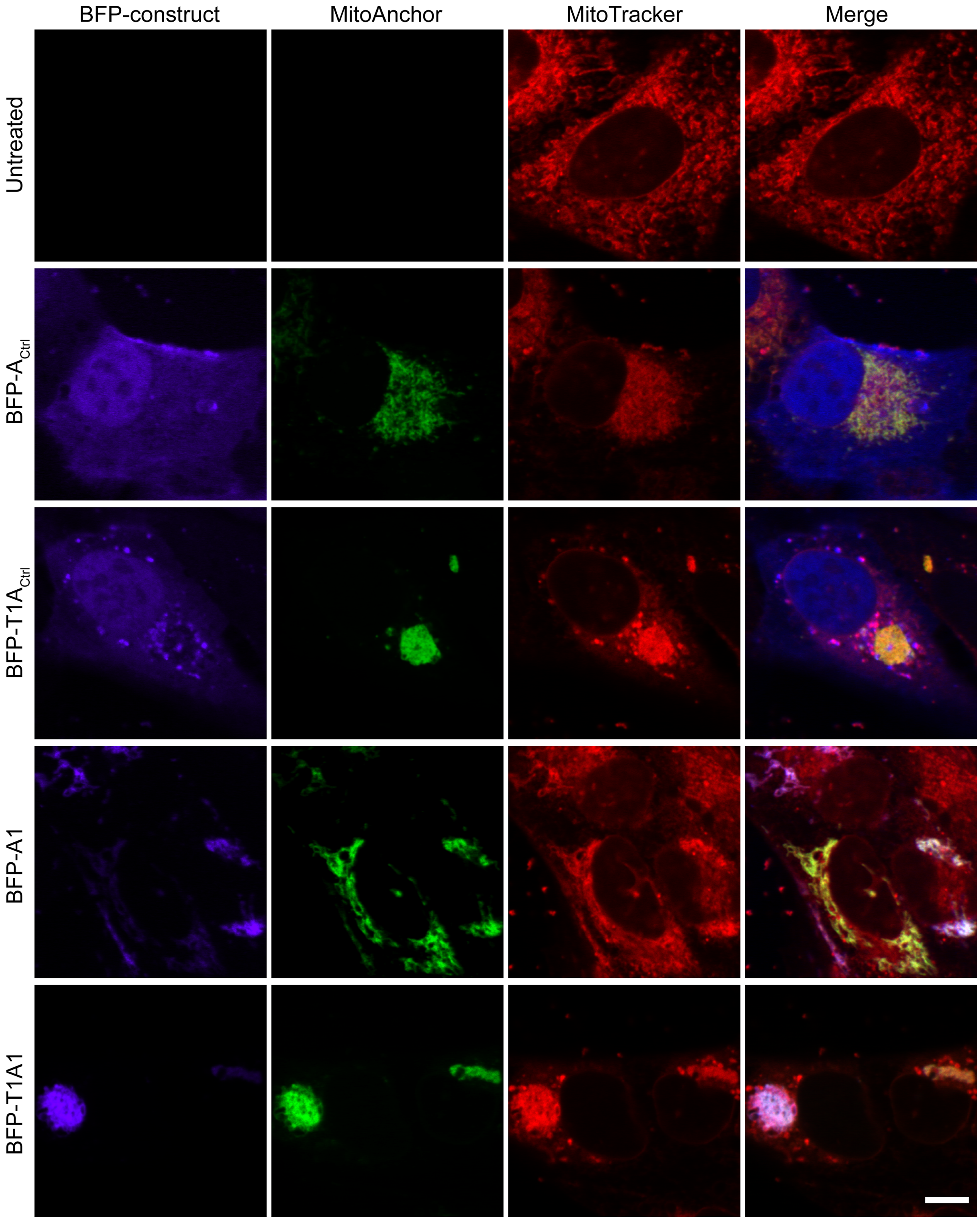

Figure S12. Representative images for the cellular localization of the BFP-based constructs, MitoAnchor (stained by SNAP-Cell Oregon Green), and MitoTracker Deep Red staining in U2OS cells. Scale bar, 10 μm.

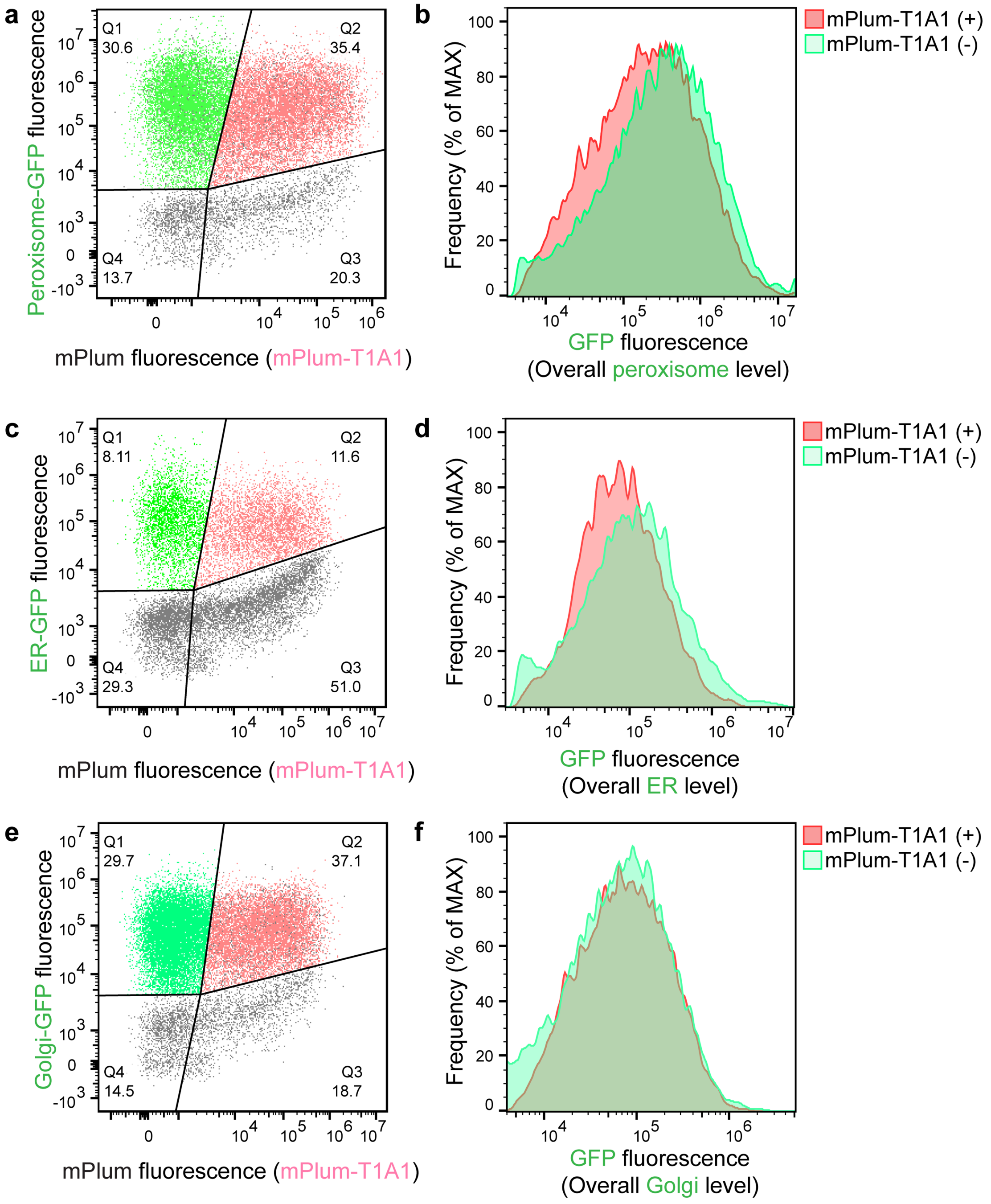

Figure S13. Flow cytometry dot plot analyses (**a**,**c**,**e**) for the mPlum-T1A1 degrader intensity and the organelle-GFP intensity in U2OS cells. The top left quadrant of the mPlum-T1A1(-) population (Q1) and the top right quadrant of the mPlum-T1A1(+) population (Q2) were plotted for their corresponding histogram analyses of the organelle-GFP intensity (**b**,**d**,**f**).

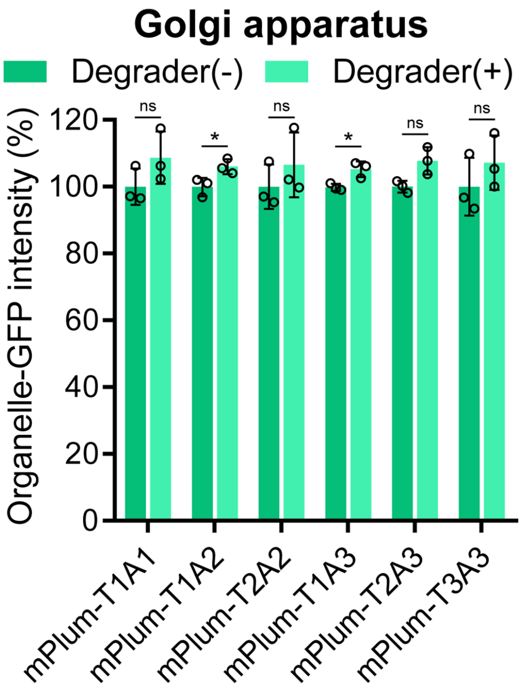

Figure S14. Effect of mPlum-T*m*A*n* degraders on GFP-labelled Golgi apparatus in U2OS cells. The quantification was conducted by flow cytometry analysis. Error bars represent standard deviations of $N = 3$. Statistical analyses are performed using two-tailed Student's t test. *, $p < 0.05$; ns, no significance.

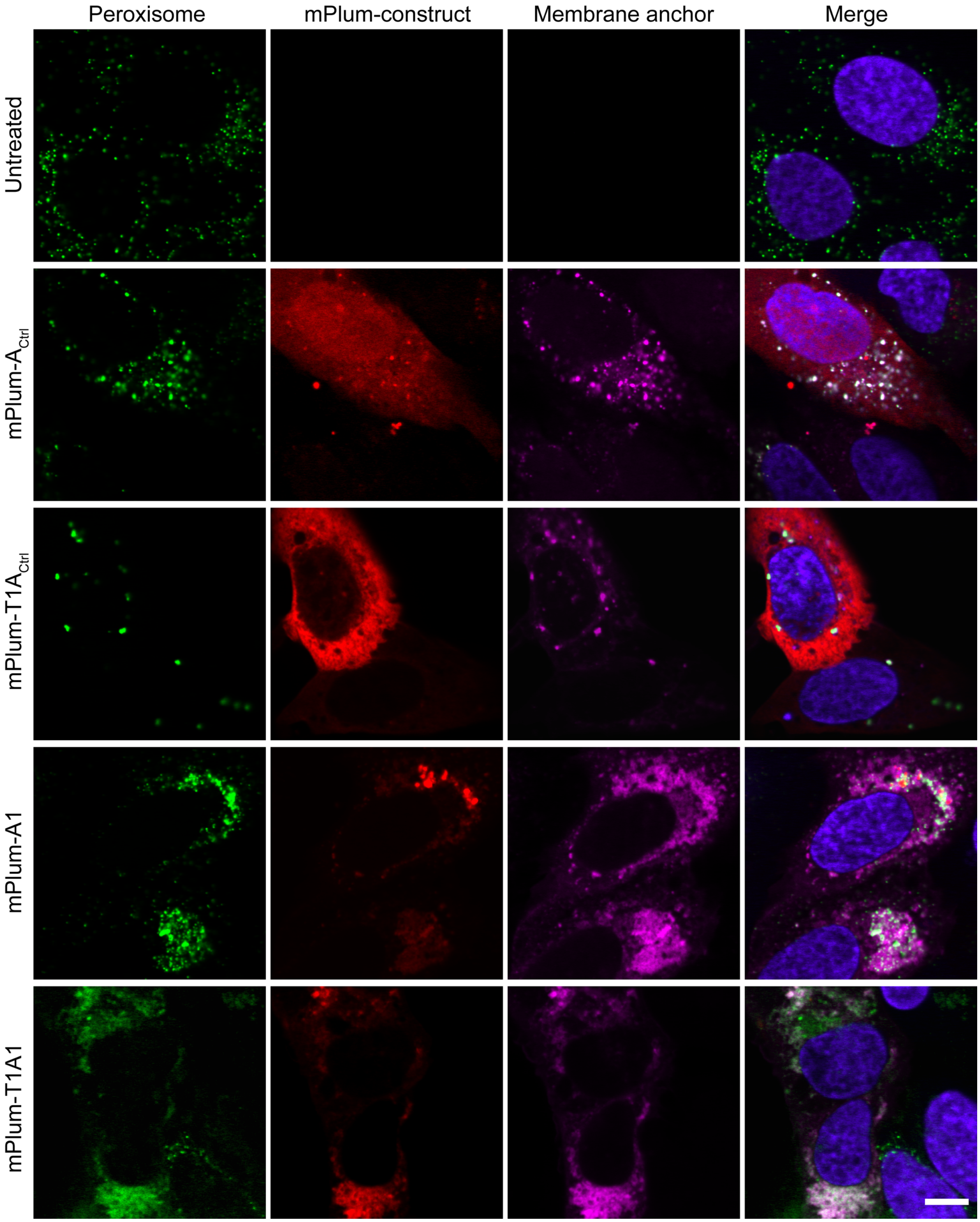

Figure S15. Representative images for the cellular localization of the GFP-labelled peroxisome, mPlum-based constructs, and Membrane anchor (for peroxisomes; Stained by SNAP-Cell 647-SiR) in U2OS cells. The merged channel also includes the nuclear staining by Hoechst 33342 (shown in blue). Scale bar, 10 μm.

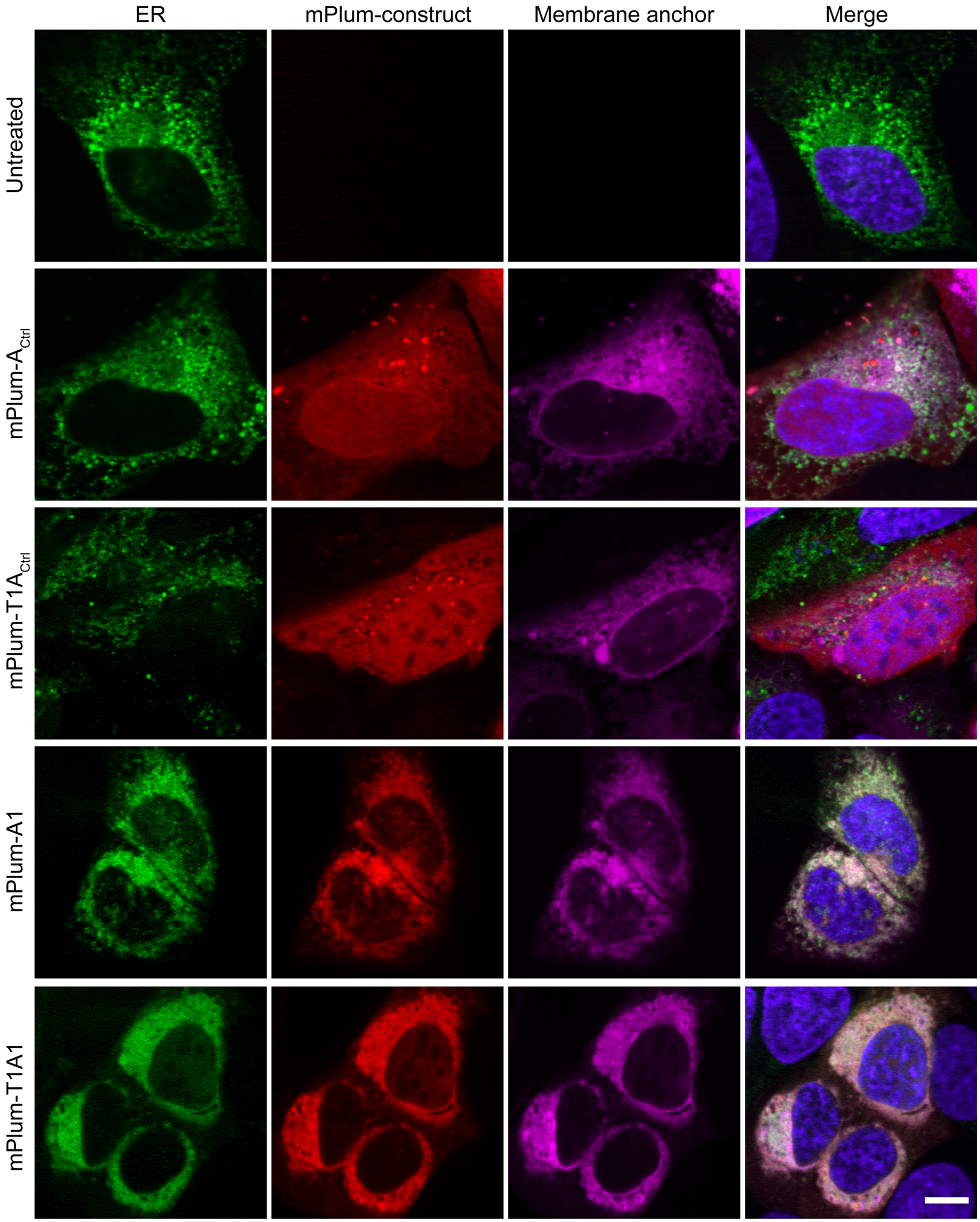

Figure S16. Representative images for the cellular localization of the GFP-labelled endoplasmic reticulum (ER), mPlum-based constructs, and Membrane anchor (for ER; Stained by SNAP-Cell 647-SiR) in U2OS cells. The merged channel also includes the nuclear staining by Hoechst 33342 (shown in blue). Scale bar, 10 μm.

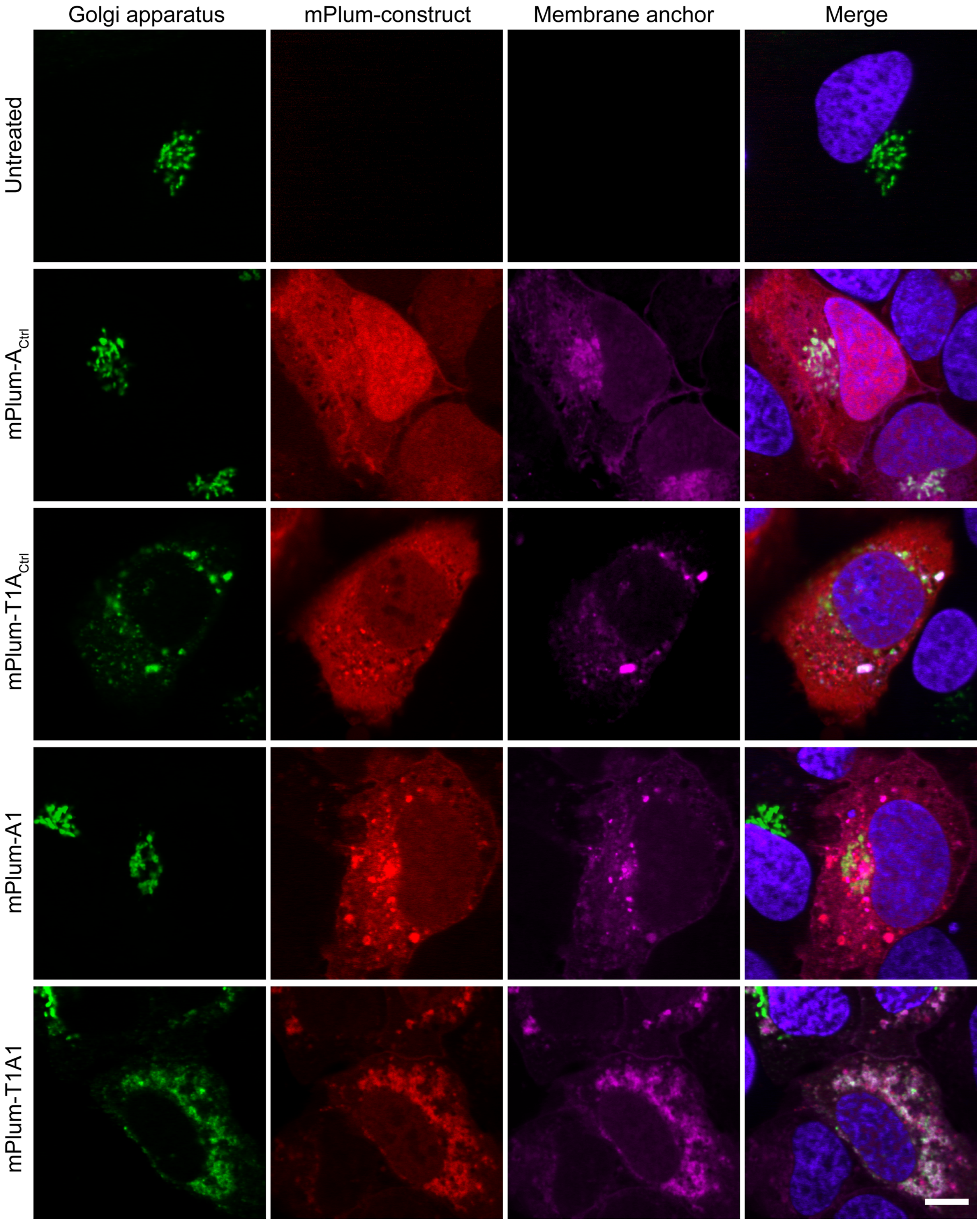

Figure S17. Representative images for the cellular localization of the GFP-labelled Golgi apparatus, mPlum-based constructs, and Membrane anchor (for Golgi apparatus; Stained by SNAP-Cell 647-SiR) in U2OS cells. The merged channel also includes the nuclear staining by Hoechst 33342 (shown in blue). Scale bar, 10 μm.

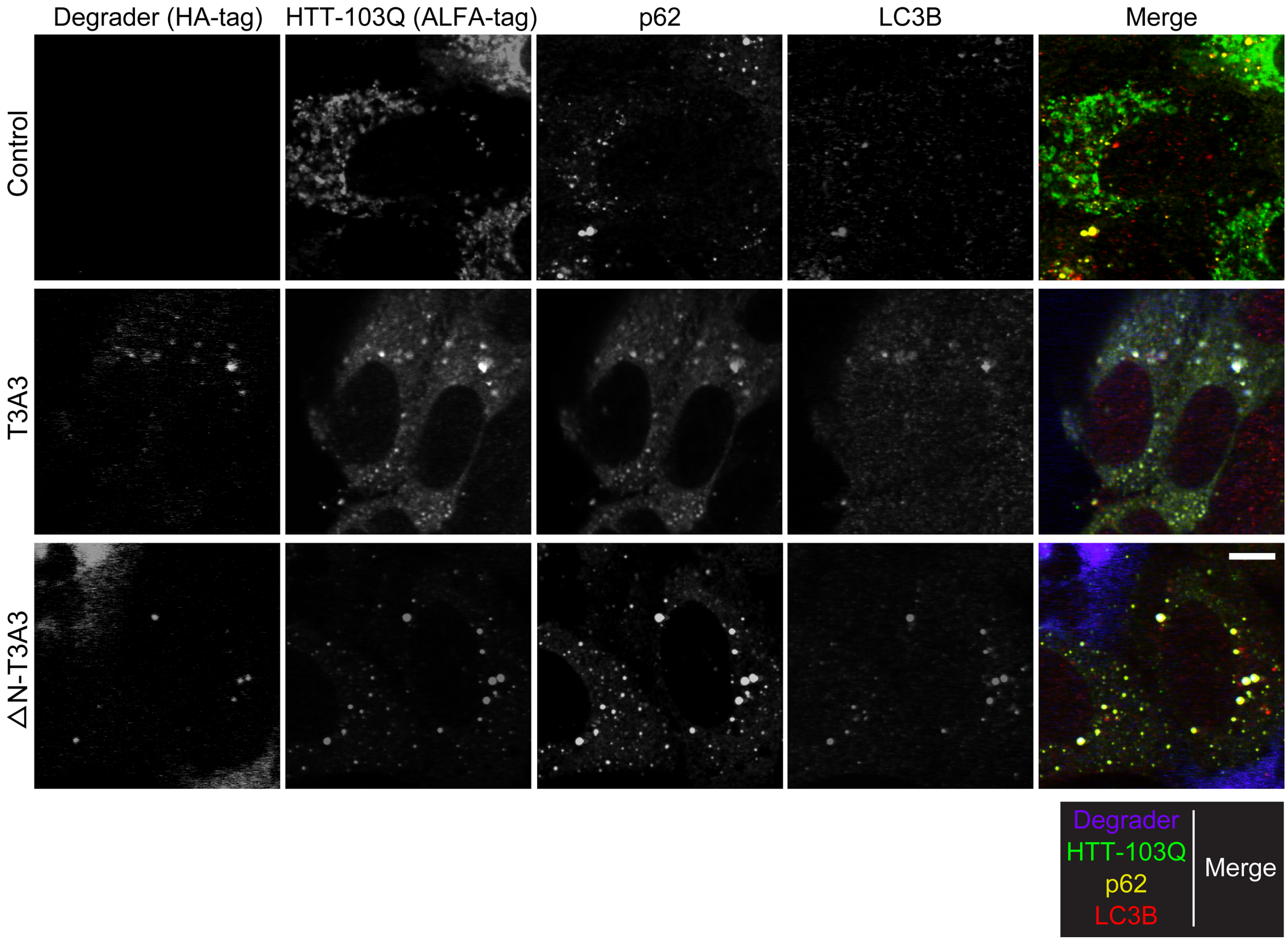

Figure S18. Representative images for the cellular localization of representative AceTAC degraders (T3A3 and ΔN-T3A3), HTT-103Q target, endogenous p62, and endogenous LC3B in U2OS cells. Each channel in Figure 5a was provided in greyscale and the merged channel was color-coded. Scale bar, 10 μm.

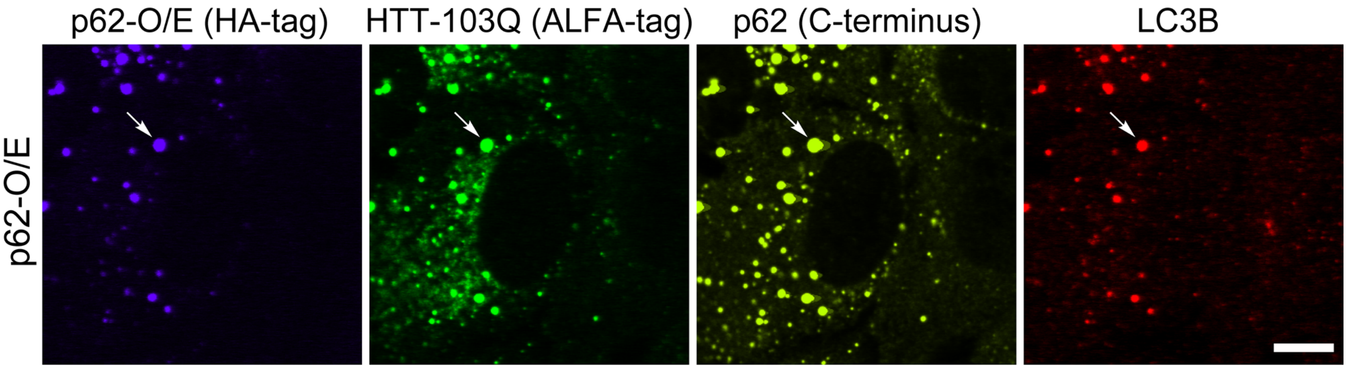

Figure S19. Representative images for the cellular localization of the p62-overexpression (HA-tagged), HTT-103Q target, endogenous p62, and LC3B in U2OS cells. Scale bar, 10 μm.

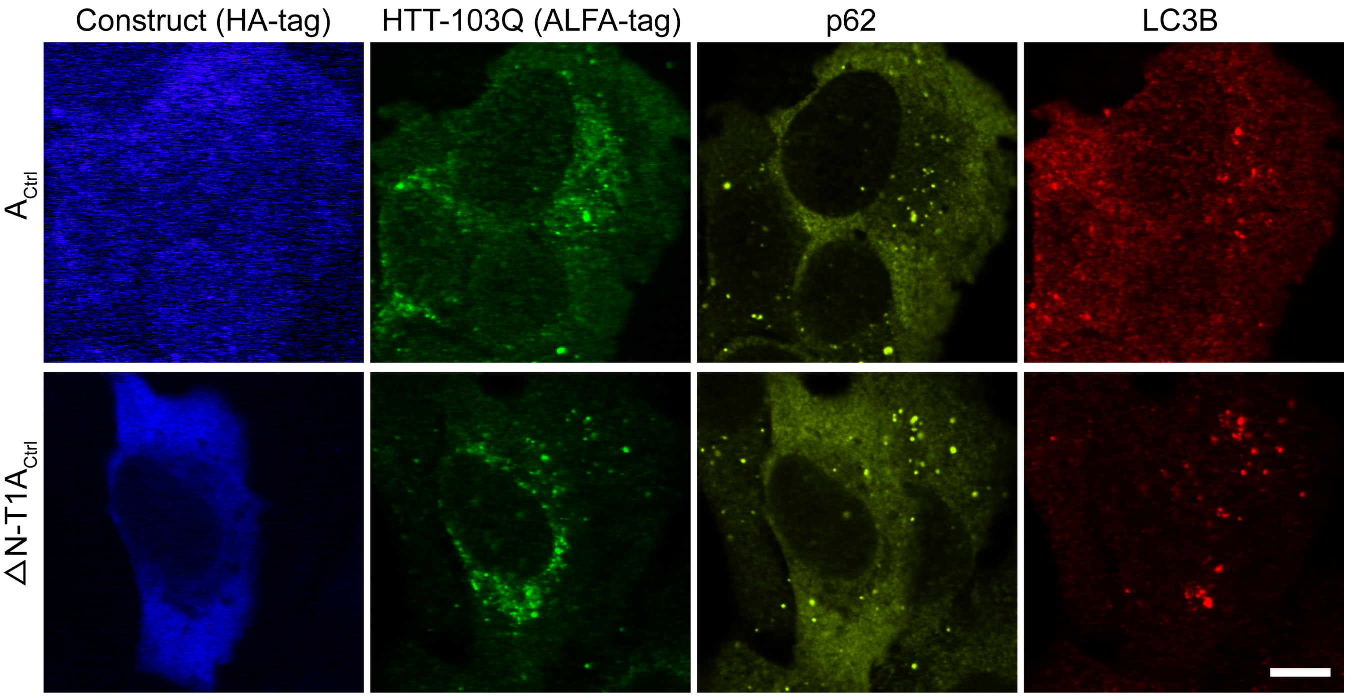

Figure S20. Representative images for the cellular localization of the control constructs, HTT-103Q target, endogenous p62, and LC3B in U2OS cells. Scale bar, 10 μm.

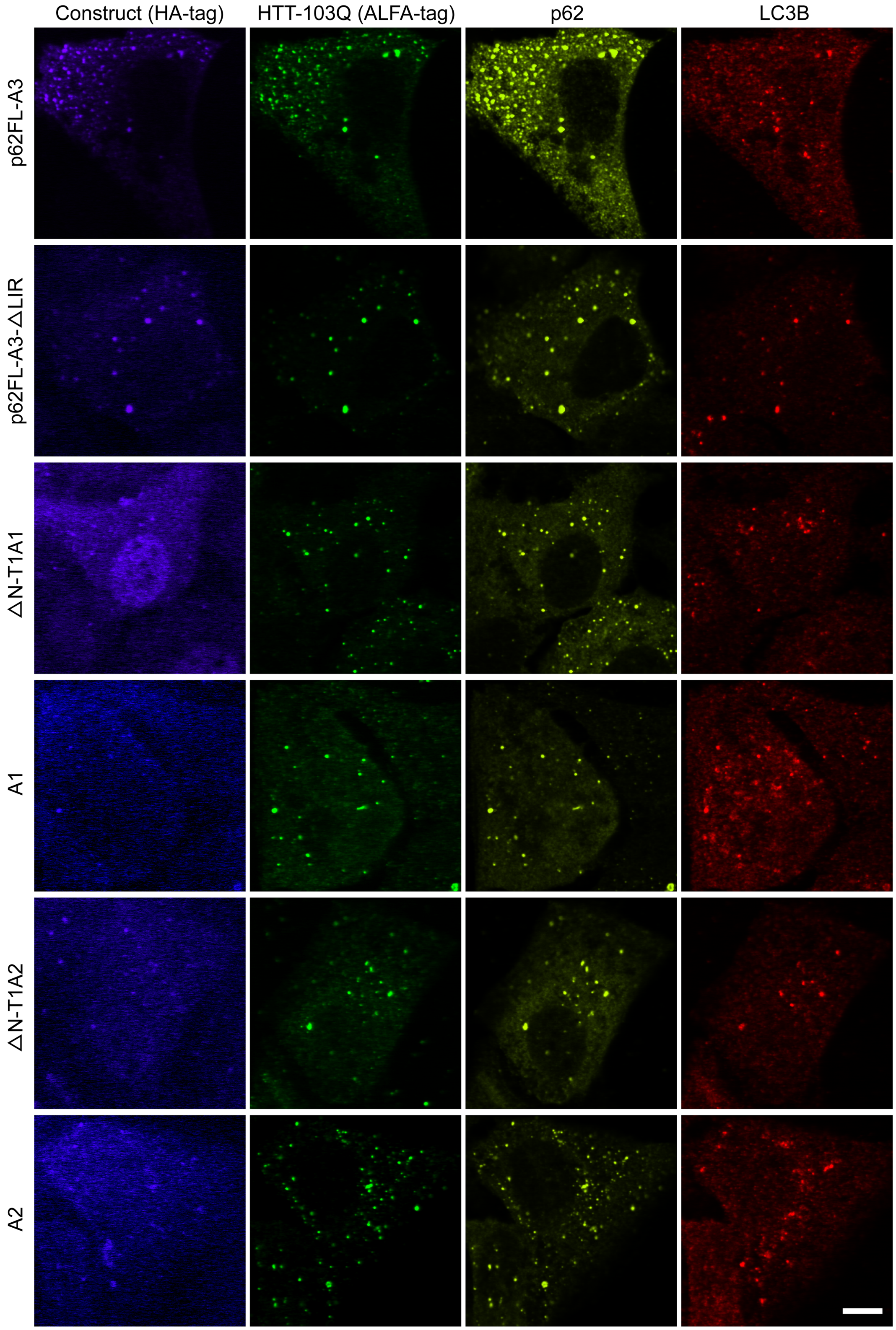

Figure S21. Representative images for the cellular localization of HA-tagged constructs, HTT-103Q target, endogenous p62, and LC3B in U2OS cells, comparing the effect of removing the LIR motif in AceTAC degraders. Scale bar, 10 μm.

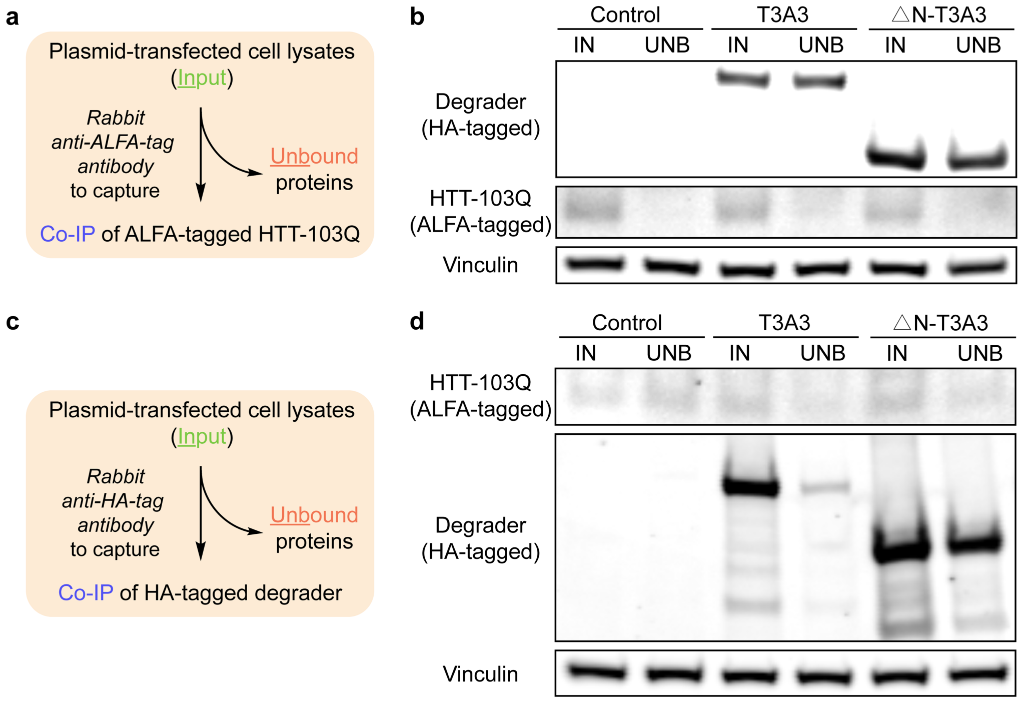

Figure S22. Workflow of the co-immunoprecipitation (co-IP) (**a,c**) and western blots for cells after plasmid transfection (**b,d**). Western blots for degraders (HA-tagged), HTT-103Q target (ALFA-tagged), and vinculin in U2OS cells were analyzed after 24-h co-transfection of plasmids that encode the degrader and the target. For each group, input proteins in the lysates (IN) before the co-IP of HTT-103Q (or HA-tagged degraders) were compared with the unbound proteins in the lysates (UNB). Control, U2OS cells with only the target-encoding plasmid transfected.

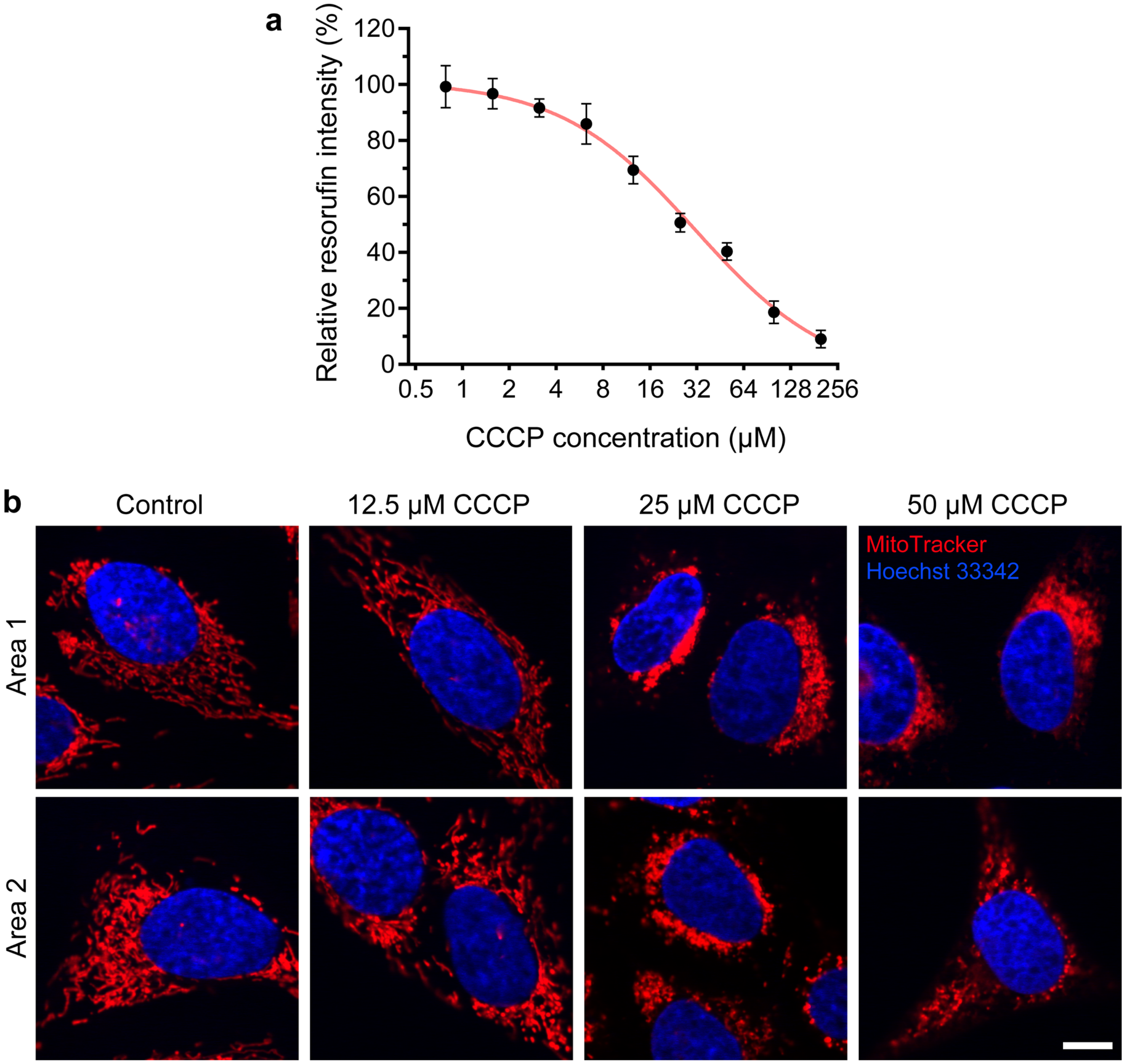

Figure S23. Dose selection for CCCP-treatment to induce mitophagy in U2OS cells. **a**, Inhibition of the cellular metabolic activity by the 24-hr treatment of CCCP (carbonyl cyanide *m*-chlorophenyl hydrazone) in U2OS cells. The fitted curve (red line) showed an IC50 value at 31.34 μM. Alamar Blue assay was used for the readout of resorufin fluorescence. Error bars represent standard deviations of $N = 6$. **b**, Representative images for the mitochondrial morphology in live U2OS cells after 24-hr CCCP treatment. From the comparison, the dose for CCCP-treatment was chosen to be 25 μM to induce mitophagy. Scale bar, 10 μm.

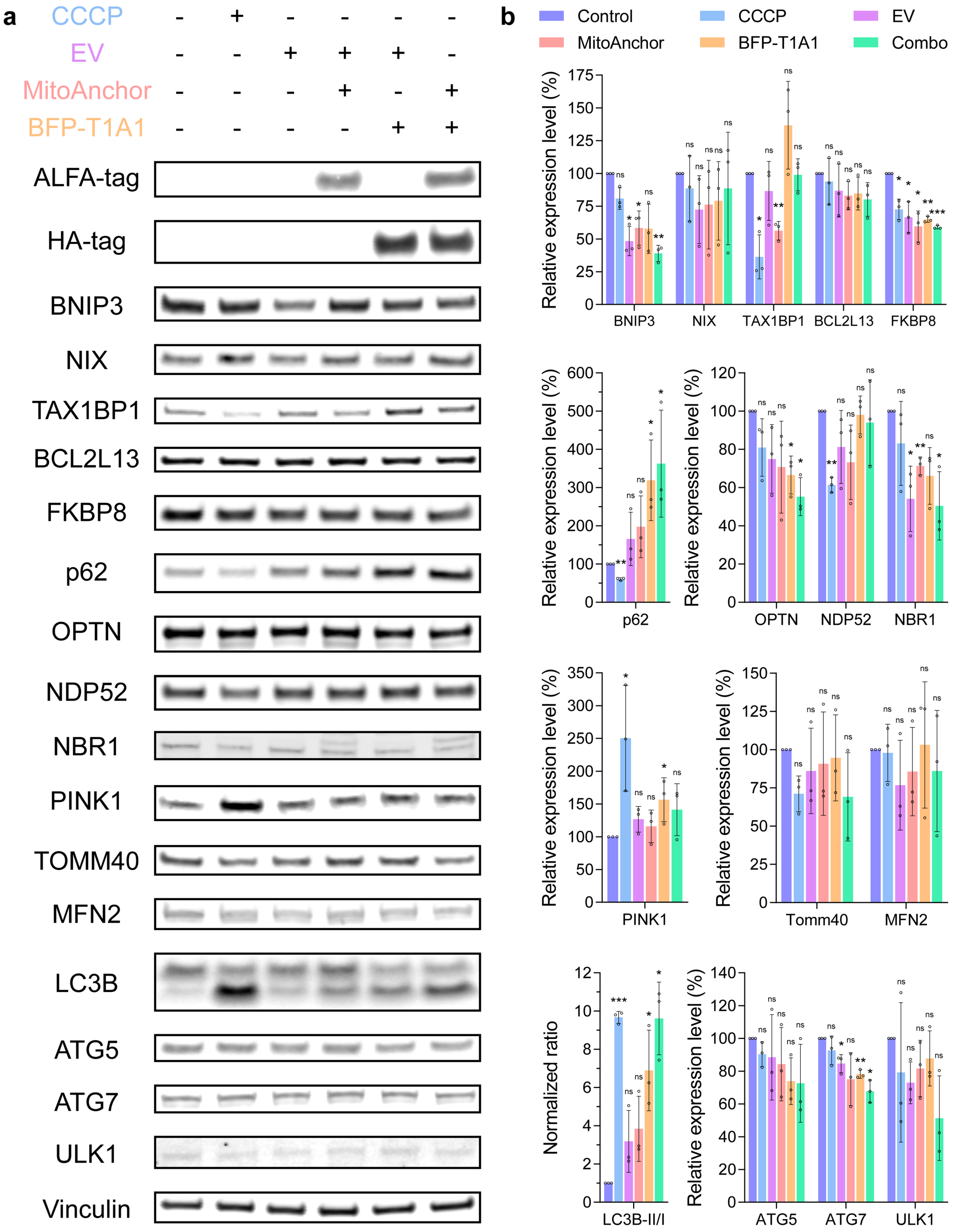

Figure S24. a, Representative western blots for selected proteins to evaluate mitophagy in U2OS cells. The CCCP dose was 25 μM for 24-hr treatment. MitoAnchor contains a C-terminal ALFA-tag and BFP-T1A1 AceTAC degrader contains a C-terminal HA-tag. Vinculin was used as the loading control. **b**, Quantification of western blots for the selected proteins in U2OS cells. Control group, U2OS cells without drug treatment or plasmid transfection. Combo group, co-presence of the MitoAnchor and the BFP-T1A1 degrader in U2OS cells. Error bars represent standard deviations of $N = 3$. Statistical analyses are performed using two-tailed Student's t test. *, $p < 0.05$; **, $p < 0.01$; ***, $p < 0.001$; ns, no significance.

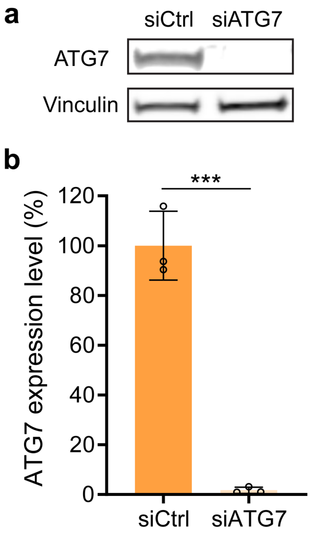

Figure S25. Western blot analysis to validate the successful knockdown of ATG7 in U2OS cells. **a**, Representative western blots for ATG7 and vinculin in U2OS cells after transfecting siRNA against ATG7 expression for 48 hours. **b**, Quantification of ATG7 expression level after ATG7-knockdown by RNA interference. Error bars represent standard deviations of $N = 3$. Statistical analyses are performed using two-tailed Student's t test. ***, $p < 0.001$.

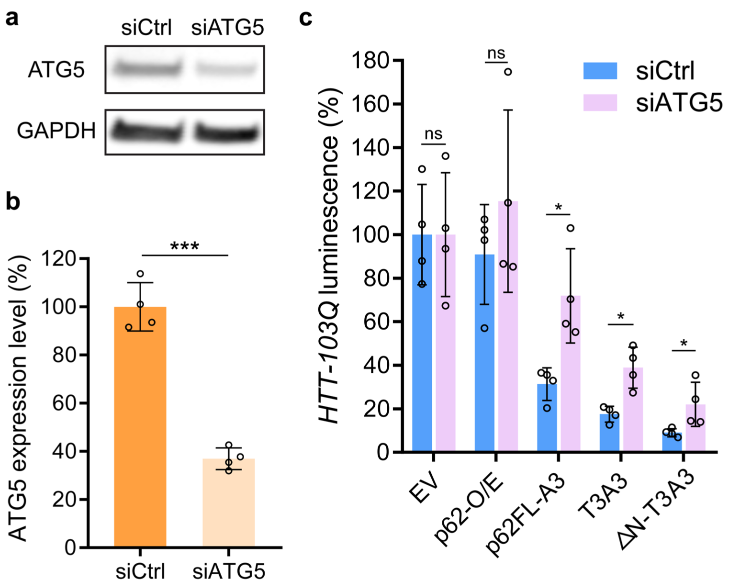

Figure S26. a,b, Western blot analysis to validate the successful knockdown of ATG5 in U2OS cells. Representative western blots for ATG5 and GAPDH in U2OS cells after transfecting siRNA against ATG5 expression for 48 hours (**a**). Quantification of ATG5 expression level after ATG5-knockdown by RNA interference (**b**). Error bars represent standard deviations of $N = 4$. Statistical analyses are performed using two-tailed Student's t test. ***, $p < 0.001$. **c**, HiBiT luminescence assay to evaluate the effect of ATG5-knockdown on the targeted HTT-103Q degradation by representative AceTAC degraders in U2OS cells. Error bars represent standard deviations of $N = 4$. Statistical analyses are performed using two-tailed Student's t test. *, $p < 0.05$; ns, no significance.

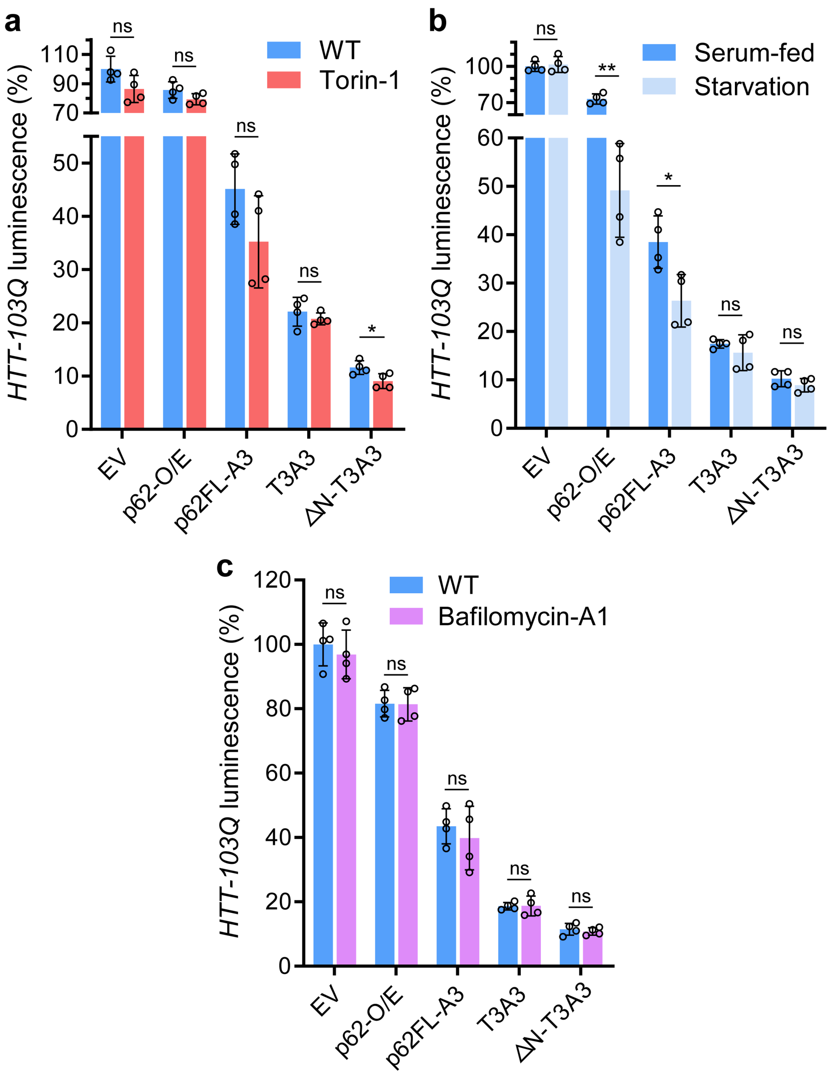

Figure S27. HiBiT luminescence assay to evaluate the effect of 250 nM Torin1 (**a**), serum starvation (**b**), and 100 nM Bafilomycin-A1 (**c**) on the targeted HTT-103Q degradation by representative AceTAC degraders in U2OS cells. Error bars represent standard deviations of $N = 4$. The autophagy-inducing (Torin-1 treatment or serum-starvation) and -inhibition (Bafilomycin-A1 treatment) conditions were applied after the 24-hour co-transfection of the target-encoding and degrader-encoding plasmids, possibly resulting in the subtle effects on the targeted protein degradation efficiency due to the limited treatment time. However, the result may be further complicated if these autophagy-modulating conditions were applied before plasmid transfection, *e.g.*, posssibly affecting the transfection efficiency. Statistical analyses are performed using two-tailed Student's t test. *, $p < 0.05$; **, $p < 0.01$; ns, no significance.

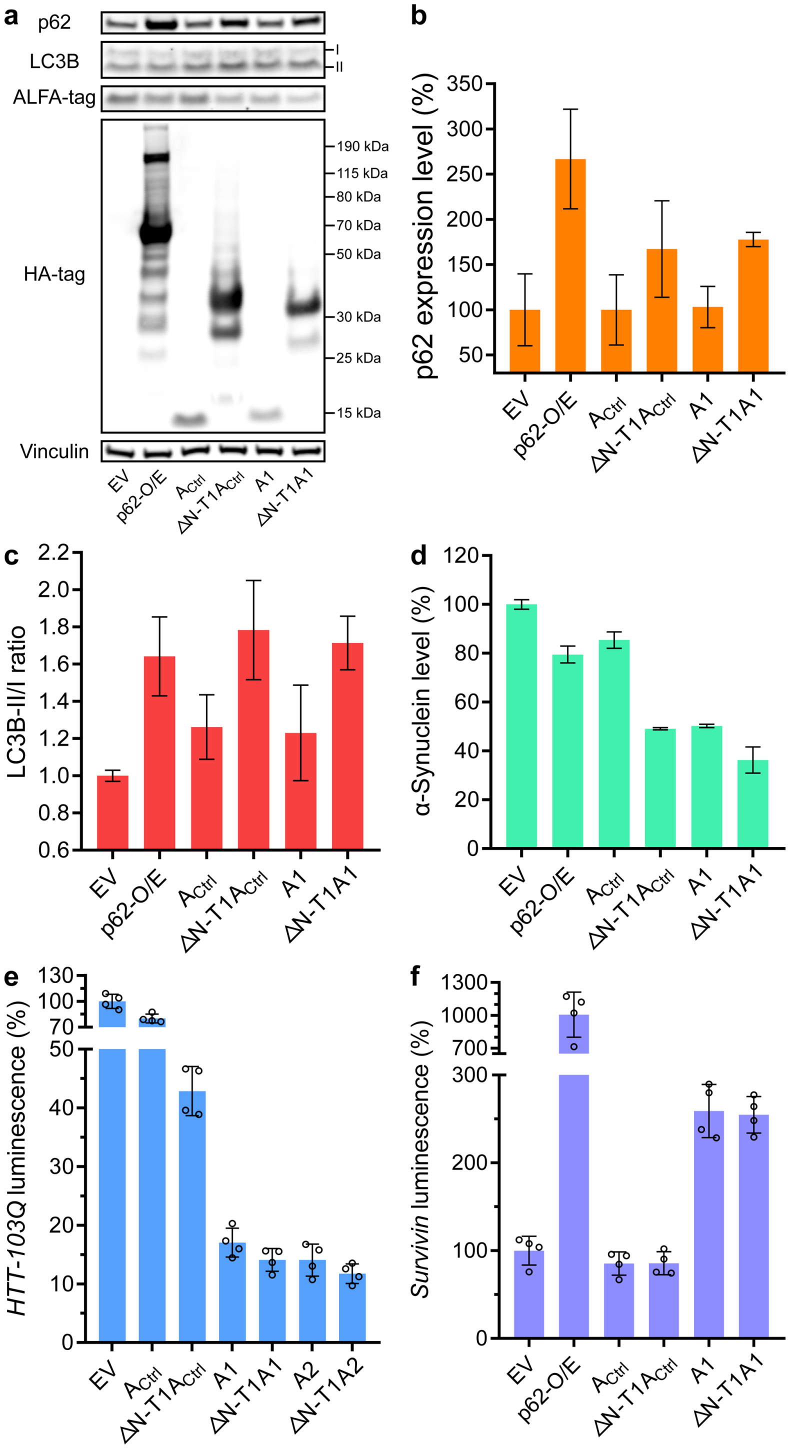

Figure S28. The LIR motif of TP53INP2 in the AceTAC degrader modulated the autophagic flux, enhancing the targeted degradation efficiency of proteins that are autophagy substrates. **a**, Representative western blots for p62, LC3B, ALFA-tagged target (α-synuclein), HA-tagged construct, and vinculin in U2OS after transfecting the construct-encoding plasmids

for 24 hours. A_{Ctrl}, control nanobody against RNase A. ΔN-T1A_{Ctrl} has a TP53INP2 LIR motif linked with the A_{Ctrl}. A_n, nanobody against the ALFA-tag; *n* represets the number of antibody repeats. **b~d**, Quantification of western blots for the p62 expression level (**b**), the LC3B-II/I ratio (**c**), and ALFA-tagged α-synuclein (**d**) in U2OS cells. The increase of p62 expression and LC3-II/I ratio along with the improved α-synuclein degradation after the addition of TP53INP2 LIR motif (ΔN-T1A_{Ctrl} *vs.* A_{Ctrl}; ΔN-T1A1 *vs.* A1) indicates the enhancement of autophagic flux. Error bars represent standard deviations of $N = 2$. **e,f**, HiBiT luminescence assay to evaluate the effect of TP53INP2 LIR motif on the selected AceTAC degraders towards the degradation of HTT-103Q (**e**) and survivin (**f**) degradation in U2OS cells. Error bars represent standard deviations of $N = 4$. For α-synuclein and HTT-103Q, either ΔN-T1A_{Ctrl} or the A1 group have caused the protein degradation. The effect from ΔN-T1A_{Ctrl} can possibly be attributed to the enhanced autophagic flux. The binding between ALFA-tag nanobody (A1) and its protein targets induced puncta formation of the target and colocalization with endogenous p62 and LC3B (shown in Figure S21), triggering the autophagy machinery. For both α-synuclein and HTT-103Q, the combination between the TP53INP2-LIR motif and the ALFA-tag nanobody led to an increased protein degradation efficiency than each component alone (ΔN-T1A1 *vs.* ΔN-T1A_{Ctrl} or A1). For survivin, the presence of either A_{Ctrl} or ΔN-T1A_{Ctrl} minimally affected the survivin level when compared with the EV group. In contrast, the survivin level was significantly increased in the presence of p62-O/E, A1, or ΔN-T1A1. It has been shown that the accumulation of p62 inhibits the degradation of ubiquitinated proteins destined for proteasomal clearance by delaying their delivery to the proteasome.[SR2] As survivin is a proteasomal substrate,[SR3] it is therefore reasonable that the increased p62 level in either the p62-O/E or ΔN-T1A1 group may have caused the excess accumulation of survivin. The tight binding between A1 and ALFA-tagged survivin may have contributed the accumulation of survivin. However, these hypotheses shall be further validated. Overall, this dataset indicates that the AceTAC degraders are more effective against proteins that are autophagy substrates rather than proteasomal substrates.

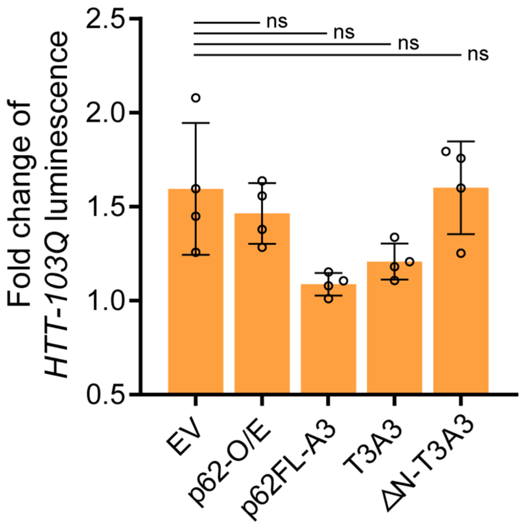

Figure S29. HiBiT luminescence assay to evaluate the effect of 200 nM carfilzomib on the targeted HTT-103Q degradation by representative AceTAC degraders in U2OS cells. The fold change describes the increase of the HiBiT luminescence value after carfilzomib-treatment. Error bars represent standard deviations of $N = 4$. Statistical analyses are performed using two-tailed Student's t test. ns, no significance.

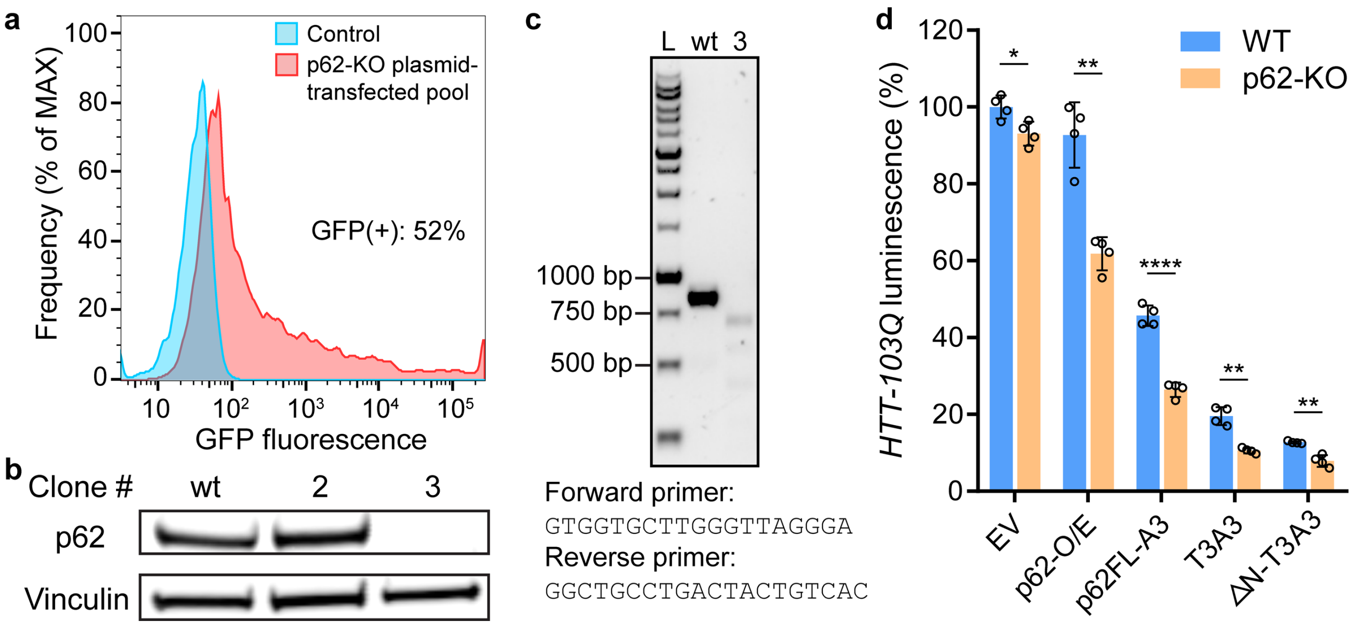

Figure S30. a~c, Generation of p62-knockout (p62-KO) U2OS cells. Flow cytometry histograms of U2OS cells transfected with p62-KO plasmids (containing EGFP-encoding sequence) (**a**). Western blots for p47 and β-actin in U2OS single cell clones (**b**). PCR amplification of the genomic DNA in wild-type (wt) U2OS cells and selected U2OS clones after p62-KO-plasmid transfection and cell sorting (**c**). **d**, HiBiT luminescence assay to evaluate the effect of p62-KO on the targeted HTT-103Q degradation by representative AceTAC degraders in U2OS cells. Error bars represent standard deviations of $N = 4$. Statistical analyses are performed using two-tailed Student's t test. *, $p < 0.05$; **, $p < 0.01$; ****, $p < 0.0001$; ns, no significance.

2. Supplementary methods

2.1. Plasmid construction

The plasmids in this work were constructed by Gibson assemblies of PCR-amplified DNA fragments. Deletion-mutant constructs were obtained by PCR-amplification and treatment of KLD enzyme mix. All the degraders were cloned into a mammalian expression vector pcDNA3.1(+). The degraders in this study contain a C-terminal HA-epitope tag (9 a.a., YPYDVPDYA). Antibody against the ALFA-epitope tag was based on a reported amino acid sequence[SR4] and codon-optimized. A 32-a.a. linker[SR5] was inserted between two units of antibodies or LIR motifs. Unless stated otherwise, all the targets for degradation were cloned into a customized mammalian expression vector under the HSV promoter (pHSV vector). The pHSV vector was designed by replacing the CMV enhancer-promoter in pcDNA3.1(+) with the HSV promoter, with the start codon 35 bp downstream from the HSV promoter. All the protein targets on the pHSV vector contain an N-terminal HiBiT tag (11 a.a., VSGWRLFKKIS) and a C-terminal ALFA-tag (13 a.a., SRLEEELRRRLTE). The doxycycline-inducible constructs for representative AceTAC degraders were based on a Tet-On 3G vector (Addgene, Cat# 96930), replacing the EGFP-coding sequence with the corresponding AceTAC degrader-coding sequence. The MitoAnchor construct was cloned into both the pcDNA3.1(+) vector and the pHSV vector. The rest of the Membrane-anchor constructs with a C-terminal ALFA-tag were built into the pcDNA3.1(+) vector. The N-terminal organelle targeting sequences were originated from previous reports: mitochondria – (Addgene, Cat# 136623), peroxisome – (Addgene, Cat# 67764), ER – (Addgene, Cat# 64855), and Golgi apparatus – (Addgene, Cat# 14873). All constructs were sequence-verified by Sanger sequencing.

2.2. Cell culture

The U2OS cell line was acquired from ATCC (Cat# HTB-96). Cells were grown at 37 °C in a humidified atmosphere (5% CO_2), cultured and passaged in Dulbecco's modified eagle medium + GlutaMAX (DMEM, ThermoFisher, Cat# 10567014) supplemented with 10% fetal bovine serum (FBS). Cells were passaged before reaching ~70% confluency, with the media replaced every 3 days.

2.3. HiBiT luminescence assay for the quantification of targeted protein degradation

A total of 3.5 k U2OS cells in 100 μL complete DMEM medium per well was seeded in a 96-well microplate (Corning, Cat# 3610) 24-hour prior to the experiment. The complexes between 90 nL Xfect transfection reagent (Takara Bio, Cat# 631318) and 300 ng plasmids [150 ng pHSV plasmids encoding the protein target and 150 ng pcDNA3.1(+) plasmids encoding the degrader/empty vector (EV; ThermoFisher, Cat# V79020)] were mixed in 10 μL Xfect reaction buffer for 10 min. After replacing the media with 90 μL fresh complete DMEM medium, the Xfect-plasmid mixture was added into the well and incubated for 24 hours. The medium that contained the Xfect-plasmid mixture was replaced with 200 μL fresh complete DMEM medium and incubated at 37 °C for another 6 hours. Meanwhile, the HiBiT lytic solution was prepared by diluting the HiBiT lytic substrate 1:50 and the LgBiT protein 1:100 into a calculated volume of HiBiT lytic buffer and further mixing with the same volume of phosphate buffer (PBS, pH 7.4). Next, the DMEM medium in each well was replaced with 150 μL HiBiT lytic solution at room temperature, followed by mixing on an orbital shaker (Benchmark Scientific, Cat# BT1502) at 600 rpm for 10 minutes. The luminescence of each well was immediately measured on a Tecan Infinite 200 PRO plate reader (Integration time: 1 second). After subtracting the value of the assay background (non-transfected U2OS cells treated with the HiBiT lytic solution), the luminescence value of the EV/target group was normalized as 100%.

2.4. Immunofluorescence (IF)

A total of 8.5 k U2OS cells in 200 μL complete DMEM medium per well were cultured in an 8-well chambered cover glass (Cellvis, Cat# C8-1.5H-N) for 24 hours before the experiment. The complexes between 0.23 μL Xfect transfection reagent and 750 ng plasmids [375 ng pHSV plasmids encoding the HTT-103Q and 375 ng pcDNA3.1(+) plasmids encoding the degrader; For the control group, 750 ng pHSV plasmids for the HTT-103Q-expression] were mixed in 10 μL Xfect reaction buffer for 10 min. After replacing the media with 130 μL fresh complete DMEM medium, the Xfect-plasmid mixture was spiked into the well. After incubating for 24 hours at 37 °C, transfected cells were rinsed once with cold PBS and fixed/permeated using the BD Fixation/Permeablization Kit (BD Biosciences, Cat# 554714). After blocking with PBST

(PBS + 0.1% Tween-20) that contains 1% BSA and 0.3 M glycine for 30 minutes, the cells were incubated at 4 °C overnight with diluted primary antibodies. After washing the cells with PBS three times, the cells were incubated with diluted secondary antibodies for 1 hour in the dark at room temperature, followed by washing with PBS. The cells in PBS were imaged on a Nikon Ti confocal microscope with a Yokagawa CSU22 spinning disk unit. The fluorescence distribution was measured with excitation wavelengths of 405 nm (Alexa Fluor 405; HA-tagged constructs), 488 nm (Alexa Fluor 488; ALFA-tagged constructs), 561 nm (Alexa Fluor 568; p62), and 647 nm (Alexa Fluor 647; LC3B). The Pearson colocalization efficient analysis was carried out using the built-in function of the Nikon NIS-Elements AR Software.

List of primary and secondary antibodies for IF (diluted in PSBT that contains 1% BSA):

Chicken anti-HA-tag antibody, Abcam, Cat# ab9111, 1:200 diluted;

Mouse anti-ALFA-tag antibody, NanoTag Biotechnologies, Cat# N1582, 1 mg·mL^{-1}, 1:500 diluted;

Guinea pig anti-p62 antibody, PROGEN, Cat# GP62-C, 1:200 diluted;

Rabbit anti-LC3B antibody, Abcam, Cat# ab192890, 1:1000 diluted;

Goat-anti-chicken secondary antibody, Alexa Fluor 405, Abcam, Cat# ab175674, 1:500 diluted;

Goat-anti-mouse secondary antibody, Alexa Fluor 488, Abcam, Cat# ab150113, 1:500 diluted;

Goat-anti-guinea pig secondary antibody, Alexa Fluor 568, Abcam, Cat# ab175714, 1:500 diluted;

Goat-anti-rabbit secondary antibody, Alexa Fluor 647, Abcam, Cat# ab150083, 1:500 diluted.

2.5. Co-immunoprecipitation (co-IP)

The general procedure for the immunoblotting experiments was based on our previous report.[SR6] For co-IP, a total of 100 k U2OS cells in complete DMEM medium were cultured in a 6-well plate (Corning, Cat# 3516) for 24 hours prior to the experiment. On the day of transfection, the complexes between 1.5 μL Xfect transfection reagent and 5 μg plasmids [2.5 μg pcDNA3.1(+) plasmids encoding HTT-103Q and 2.5 μg pcDNA3.1(+) plasmids encoding the EV or the degrader] were mixed in 100 μL Xfect reaction buffer for 10 min. The media were replaced with 900 μL fresh complete DMEM medium and

the Xfect-plasmid mixture was added dropwise into the well to incubate at 37 °C for 24 hours. Cell lysis was conducted with the NP-40 lysis buffer and the cell debris were discarded after centrifugation. The supernatant was collected as the cell lysates.

The cell lysates (~300 μL) were mixed with 3 μL rabbit anti-ALFA-tag antibodies (for the co-IP of ALFA-tagged HTT-103Q; NanoTag Biotechnologies, Cat# N1583, 1 mg·mL^{-1}) (or rabbit anti-HA-tag antibodies for the co-IP of HA-tagged constructs; CST, Cat# 3724S) and incubated overnight at 4 °C. Next, part of the lysates (200 μL) were incubated with the Protein-A magnetic beads (ThermoFisher, Cat# 88845), and the leftover lysates were labeled as the INPUT group. After one-hour incubation at room temperature, the beads were collected by a magnet and eluted at 90 °C for 5 min in 1X LDS sample buffer, resulting in the elution as the co-IP portion of the corresponding group. The lysates containing the unbound proteins were collected as the UNBOUND group).

For western blotting of the INPUT and UNBOUND groups, protein levels in each group were quantified with BCA assay and diluted to approximately equal concentrations with NP-40 lysis buffer. After heating in 1X LDS sample buffer at 90 °C for 5 min, equal amounts of INPUT and UNBOUND samples were loaded into lanes of a Bolt 4~12% bis-tris gel (ThermoFisher, Cat# NW04122BOX, NW04125BOX, or NW04127BOX; 90 V constant for 100 min). For co-IP samples, ~15 μL elution from each group was loaded in each lane. After transferring proteins from the gel to a PVDF membrane using the iBlot-2 gel transfer system, the transferred PVDF membrane was blocked with TBS blocking buffer (LI-COR Biosciences, Cat# 927-60001) at room temperature for an hour. The membrane was probed with primary antibodies overnight at 4 °C. After washing with 1X TBST (50 mM Tris·HCl, 150 mM NaCl, 0.1% Tween-20) three times, the membrane was incubated in secondary antibodies for 1 h at room temperature. The membranes were washed with 1X TBST imaged on an Odyssey CLx infrared imaging system (LI-COR Biosciences).

List of primary and secondary antibodies for co-IP and western blots (diluted in TBS blocking buffer containing 0.1% Tween-20):

Mouse anti-HA-tag antibody, CST, Cat# 2367S, 1:1000 diluted;

Rabbit anti-HA-tag antibody, CST, Cat# 3724S, 1:1000 diluted;

Mouse anti-ALFA-tag antibody, NanoTag Biotechnologies, Cat# N1582, 1:1000 diluted;

Rabbit anti-vinculin antibody, CST, Cat# 13901S, 1:2000 diluted;

Goat-anti-rabbit secondary antibody, AzureSpectra 700 conjugates, Azure Biosystems, Cat# AC2128, 1:5000 diluted;

Goat-anti-mouse secondary antibody, AzureSpectra 700 conjugates, Azure Biosystems, Cat# AC2129, 1:5000 diluted;

Goat-anti-rabbit secondary antibody, AzureSpectra 800 conjugates, Azure Biosystems, Cat# AC2134, 1:5000 diluted;

Goat-anti-mouse secondary antibody, AzureSpectra 800 conjugates, Azure Biosystems, Cat# AC2135, 1:5000 diluted.

2.6. Autophagy modulation assays

2.6.1. ATG7-knockdown assay

The RNA interference of ATG7 was conducted through reverse transfection of the siRNA for ATG7 (siATG7; CST, Cat# 6604). The complexes between 0.5 μL Xfect-RNA transfection reagent (Takara Bio, Cat# 631450) and 5 pmol siATG7 [or the control siRNA (siCtrl); MilliporeSigma, Cat# SIC001] were mixed in 10 μL Xfect reaction buffer for 10 min and added into a 96-well microplate (Corning, Cat# 3610). A total of 8 k U2OS cells in 90 μL complete DMEM medium per well were added into the microplate and incubated for 24 hours at 37 °C. Next, the HiBiT luminescence assay was conducted on these siRNA-transfected U2OS cells.

The knockdown of ATG7 was validated by western blots. Briefly, with the siRNA-transfection reagents scaled up from a 96-well microplate to a 12-well microplate, a total of 80 k U2OS cells in complete DMEM medium per well were added into in a 12-well microplate (Corning, Cat# 3513) for 24 hours. The siRNA-complex-containing medium was replaced with fresh complete DMEM medium and incubated for another 24 hours. Next, the western blotting of U2OS cells was conducted following the "Western blots" section.

List of primary antibodies for western blots (diluted in TBS blocking buffer containing 0.1% Tween-20):

Rabbit anti-vinculin antibody, CST, Cat# 13901S, 1:2000 diluted;

Mouse anti-ATG7 antibody, Proteintech, Cat# 67341-1-Ig, 1:500 diluted.

2.6.2. ATG5-knockdown assay

The RNA interference of ATG5 was conducted through reverse transfection of the siRNA for ATG5 (siATG5; Integrated DNA Technologies, sense strand: 5'-ACGCUAAAAGGCUUACAGUAUCAGA-3'). The complexes between 0.5 μL Xfect-RNA transfection reagent (Takara Bio, Cat# 631450) and 5 pmol siATG5 [or the control siRNA (siCtrl); MilliporeSigma, Cat# SIC001] were mixed in 10 μL Xfect reaction buffer for 10 min and added into a 96-well microplate (Corning, Cat# 3610). A total of 5 k U2OS cells in 90 μL complete DMEM medium per well were added into the microplate and incubated for 24 hours at 37 °C. Next, the HiBiT luminescence assay was conducted on these siRNA-transfected U2OS cells.

The knockdown of ATG5 was validated by western blots. Briefly, with the siRNA-transfection reagents scaled up from a 96-well microplate to a 12-well microplate, a total of 60 k U2OS cells in complete DMEM medium per well were added into in a 12-well microplate (Corning, Cat# 3513) for 24 hours. The siRNA-complex-containing medium was replaced with fresh complete DMEM medium and incubated for another 24 hours. Next, the western blotting of U2OS cells was conducted following the "Western blots" section.

List of primary antibodies for western blots (diluted in TBS blocking buffer containing 0.1% Tween-20):

Mouse anti-GAPDH antibody, SCBT, Cat# sc-47724, 1:5000 diluted;

Rabbit anti-ATG5 antibody, CST, Cat# 12994, 1:1000 diluted.

2.6.3. Small-molecule modulation and serum-starvation assays

The HiBiT luminescence assay was conducted with modifications after plasmid transfection. For the treatment groups, complete DMEM medium containing Torin1 (250 nM) or Bafilomycin-A1 (100 nM), or amino acid-depleted DMEM medium (for the serum-starvation condition; USBio, Cat# D9800-13) was used to replace the transfection-complex-containing medium, followed by incubation for 6 hours at 37 °C. The WT-control group was following the original HiBiT luminescence assay procedure that uses complete DMEM medium for the 6-hour incubation. After subtracting the value of the assay background (non-transfected U2OS cells treated with the HiBiT lytic solution), the luminescence value of the EV/target group in the WT-control group was normalized as 100%. For the evaluation of the ubiquitin-proteasome pathway, the carfilzomib-treatment (200 nM) was conducted following the same protocol.

2.6.4. P62-knockout (p62KO) assay

Generation of p62KO-U2OS cells were generated based on our previous report for the generation of p47KO-U2OS cells.[SR6] The p62KO-plasmid was acquired from SCBT, Cat# sc-400099. After cell-sorting and expansion, only three viable clones were obtained. Clone# C3 was verified by western blots and genomic DNA analysis using Sanger sequencing to be the p62KO U2OS clone, thus referred as the "p62-KO U2OS cell". The p62KO assay was then conducted with the "HiBiT luminescence assay" by comparing between wildtype U2OS and p62-KO U2OS cells for the HTT-103Q degradation.

2.7. Western blots

The general procedure for the immunoblotting experiments was based on our previous report.[SR6] A total of 45 k U2OS cells in 1 mL complete DMEM medium per well were cultured in a 12-well plate (Corning, Cat# 3513) for 24 hours prior to the experiment. On the day of transfection, the complexes between 0.75 μL Xfect transfection reagent and 2.5 μg plasmids [1.25 μg pHSV plasmids encoding the protein target and 1.25 μg pcDNA3.1(+) plasmids encoding the EV or the degrader] were mixed in 50 μL Xfect reaction buffer for 10 min. The medium was replaced with 450 μL fresh complete DMEM medium and the Xfect-plasmid mixture was added dropwise into the well to incubate at 37 °C for 24 hours. Cell lysis was conducted with the RIPA lysis buffer (ThermoFisher, Cat# 89900) and the supernatant after centrifugation was collected as the cell lysates. The protein gel electrophoresis, protein transfer, antibody probing on PVDF membranes were following the "Co-IP" section.

List of primary antibodies for western blots (diluted in TBS blocking buffer containing 0.1% Tween-20):

Mouse anti-LC3B antibody, CST, Cat# 83506, 1:1000 diluted;

Rabbit anti-p62 antibody, Abcam, Cat# ab109012, 1:1000 diluted;

Mouse anti-vinculin antibody, SCBT, Cat# sc-73614, 1:2000 diluted.

2.8. Quantification of targeted organelle degradation

Mitochondria. A total of 20 k U2OS cells in 500 μL complete DMEM medium per well were cultured in a 24-well microplate (Corning, Cat# 3524) for 24 hours prior to the experiment. The complexes between 0.3 μL Xfect transfection reagent and 1 μg plasmids (500 ng plasmids encoding the BFP-tagged degrader and 500 ng plasmids encoding the MitoAnchor) were mixed in 25 μL Xfect reaction buffer for 10 min. After replacing the media with 250 μL fresh complete DMEM medium, the Xfect-plasmid mixture was added dropwise into the well and incubated at 37 °C for 24 hours. Next, the transfected cells were stained with 25 nM MitoTracker-DR (ThermoFisher, Cat# M22426) in 500 μL complete FluoroBrite DMEM medium (ThermoFisher, Cat# A1896701; containing 10% FBS) at 37 °C for 15 minutes. The cells were rinsed with cold PBS and detached by TrypLE enzyme (ThermoFisher, Cat# 12604013). The fluorescence intensity within the cells was measured using flow cytometry (Beckman CytoFLEX flow cytometer) with excitation wavelengths of 405 nm (BFP) and 638 nm (MitoTracker-DR). U2OS cells without plasmid transfection and MitoTracker staining were used as the blank control group.

Peroxisome, ER, and Golgi apparatus. A total of 20 k U2OS cells in complete DMEM medium were cultured with 4 μL BacMam GFP transduction reagents (ThermoFisher, Cat# C10604, Cat# C10590, or Cat# C10592) per well in a 24-well microplate for 24 hours, resulting in the targeted organelles labeled with GFP. After co-transfecting the mPlum-tagged-degrader- and Membrane-anchor-encoding plasmids, the cells were detached for flow cytometry measurement. The fluorescence intensity within the cells was measured using flow cytometry with excitation wavelengths of 488 nm (GFP) and 638 nm (mPlum). U2OS cells without BacMam transduction and plasmid transfection were used as the blank control group.

2.9. Fluorescence imaging of organelle targeting for AceTAC degradation system

For mitochondria-related imaging, a total of 8.5 k U2OS cells in 200 μL complete DMEM medium per well were cultured in an 8-well chambered cover glass for 24 hours before the experiment. The complexes between 0.23 μL Xfect transfection reagent and 750 ng plasmids (equal amount of degrader- and MitoAnchor-encoding plasmids) were mixed in 10 μL Xfect reaction buffer for 10 min. After replacing the media with 130 μL fresh complete DMEM medium, the Xfect-plasmid mixture was spiked into the well and incubated for 24 hours at 37 °C. cells were first stained with 5 μM SNAP-Cell Oregon Green (NEB, Cat# S9102S) in complete FluoroBrite DMEM medium at 37 °C for 30 minutes. After rinsing with FluoroBrite

DMEM once, the cells were stained with with 25 nM MitoTracker-DR in FluoroBrite DMEM at 37 °C for 15 minutes. The cells were then rinsed once with PBS and ready for live-cell imaging in FluoroBrite DMEM. The IF experiment of LC3B was carried out at this step following the procedures in the "Immunofluorescence (IF)" section.

For the imaging of the GFP-labeled organelles, a total of 8.5 k U2OS cells in complete DMEM medium were cultured with 4.3 µL BacMam GFP transduction reagents (based on the organelle) in an 8-well chambered cover glass 24 hours prior to the experiments. After co-transfecting the degrader- and Membrane-anchor-encoding plasmids, U2OS cells were first stained with 3 µM SNAP-Cell 647-SiR (NEB, Cat# S9102S) in complete FluoroBrite DMEM medium at 37 °C for 30 minutes. After rinsing with FluoroBrite DMEM once, the cells were incubated with NucBlue Live Cell Stain (ThermoFisher, Cat# R37605) in FluoroBrite DMEM at 37 °C for 5 minutes. The cells were then rinsed once with PBS and ready for live-cell imaging in FluoroBrite DMEM (Note that cells can also be fixed at this point following the procedures in the "Immunofluorescence" section). The fluorescence imaging was carried out on a Nikon Ti confocal microscope with a Yokagawa CSU22 spinning disk unit or an ECHO Revolve Microscope. For the confocal microscope, the fluorescence distribution was measured with excitation wavelengths of 405 nm, 488 nm, 561 nm, and 647 nm. For the Revolve microscope, the fluorescence distribution was measured in three channels: DAPI (Ex: 380/30 Em: 450/50, DM: 425), FITC (Ex: 470/40, Em: 525/50, DM:495) and CY5 (Ex: 630/40, Em: 700/75, DM: 660).

2.10. AlamarBlue cell viability assay

A total of 3.5 k U2OS cells in 100 µL complete DMEM medium per well was seeded in a 96-well microplate (Corning, Cat# 3596) 24-hour prior to the experiment. The complexes between 90 nL Xfect transfection reagent (Takara Bio, Cat# 631318) and 300 ng plasmids [150 ng pcDNA3.1(+) plasmids encoding the corresponding membrane-anchor construct and 150 ng pcDNA3.1(+) plasmids encoding the degrader/empty vector (EV)] were mixed in 10 µL Xfect reaction buffer for 10 min. After replacing the media with 90 µL fresh complete DMEM medium, the Xfect-plasmid mixture was added into the well and incubated for 24 hours. Next, the cell viability assay was conducted with the alamarBlue HS cell viability reagent (1X diluted in the complete DMEM medium, 220 µL in total; ThermoFisher, Cat# A50101) following the protocol provided

by the manufacturer. After incubating at 37 °C for 80 minutes, 200-μL assaying medium from each well was transferred to a 96-well microplate (Corning, Cat# 3915) and measured on a Tecan Infinite 200 PRO plate reader (Fluorescence: Ex: 560 nm, Em: 590 nm). After subtracting the value of the assay background (alamarBlue-containing medium incubated in parallel without cells), the fluorescence value of the EV+membrane-anchor group was normalized as 100%.

2.11. Evaluation of AceTAC-induced mitophagy

2.11.1. CCCP dose optimization

A total of 3.5 k U2OS cells in 100 μL complete DMEM medium per well was seeded in a 96-well microplate 24-hour prior to the experiment. The media were then replaced with fresh complete DMEM growth medium that contained calculated concentrations of CCCP (CCCP, Cat# sc-202984). The CCCP concentrations were tested from 200 μM with 2-fold serial dilutions. Cells were incubated with the CCCP-containing medium for 24 hours at 37 °C. Next, the cellular metabolic activity was assessed by the AlamarBlue assay following the procedures described in Section 2.10. AlamarBlue cell viability assay, with a change in the incubation time for the AlamarBlue reagent from 80-min to 150-min.

Next, the mitochondrial morphology of U2OS cells treated with selected CCCP concentrations was evaluated by live cell imaging. Briefly, a total of 8.5 k U2OS cells in 200 μL complete DMEM medium per well were cultured in an 8-well chambered cover glass 24 hours prior to the experiments. After treatment with selected CCCP concentrations in complete DMEM growth medium at 37 °C for 24 hours, the cells were rinsed once with PBS, followed by staining with the MitoTracker Deep Red (25 nM) and the NucBlue Live Cell Stain (2 drops per 1 mL complete FluoroBrite DMEM medium) simultaneously at 37 °C for 15-min. The cells were then rinsed once with PBS and ready for live-cell imaging in FluoroBrite DMEM. The fluorescence imaging was carried out on a Nikon Ti confocal microscope with a Yokagawa CSU22 spinning disk unit. For the confocal microscope, the fluorescence distribution was measured with excitation wavelengths of 405 nm and 647 nm.

2.11.2. Western blots of autophagy/mitophagy-related proteins

A total of 100 k U2OS cells in 2 mL complete DMEM medium per well were cultured in a 6-well plate (Corning, Cat# 3516) for 24 hours prior to the experiment. For the CCCP-treatment group, the medium was replaced with 1 mL fresh complete DMEM growth medium that contains 25 μM CCCP. For the transfection-groups, on the day of transfection, the complexes between 1.5 μL Xfect transfection reagent and 5 μg plasmids (EV-only group; for the co-transfection groups, 2.5 μg of each plasmid was included) were mixed in 100 μL Xfect reaction buffer for 10 min. The media were replaced with 1 mL fresh complete DMEM medium and the Xfect-plasmid mixture was added dropwise into the well to incubate at 37 °C for 24 hours. Cell lysis was conducted with the RIPA lysis buffer (ThermoFisher, Cat# 89900) and the supernatant after centrifugation was collected as the cell lysates. The protein gel electrophoresis, protein transfer, antibody probing on PVDF membranes were following the "Co-IP" section.

List of additional primary antibodies for western blots (diluted in TBS blocking buffer containing 0.1% Tween-20):

Mouse anti-BNIP3 antibody, Proteintech, Cat# 68091-1-Ig, 1:500 diluted;

Mouse anti-NIX antibody, Proteintech, Cat# 68118-1-Ig, 1:500 diluted;

Rabbit anti-TAX1BP1 antibody, Proteintech, Cat# 14424-1-AP, 1:1000 diluted;

Rabbit anti-BCL2L13 antibody, Proteintech, Cat# 16612-1-AP, 1:1000 diluted;

Rabbit anti-FKBP8 antibody, Proteintech, Cat# 11173-1-AP, 1:1000 diluted;

Rabbit anti-OPTN antibody, Proteintech, Cat# 10837-1-AP, 1:1000 diluted;

Rabbit anti-NDP52 antibody, Proteintech, Cat# 12229-1-AP, 1:1000 diluted;

Rabbit anti-NBR1 antibody, Proteintech, Cat# 16004-1-AP, 1:1000 diluted;

Rabbit anti-PINK1 antibody, Proteintech, Cat# 23274-1-AP, 1:1000 diluted;

Mouse anti-TOMM40 antibody, Proteintech, Cat# 66658-1-Ig, 1:1000 diluted;

Mouse anti-MFN2 antibody, Proteintech, Cat# 67487-1-Ig, 1:1000 diluted;

Rabbit anti-ULK1 antibody, CST, Cat# 8054, 1:1000 diluted.

2.12. RT-qPCR

A total of 40 k U2OS cells in 1 mL complete DMEM medium per well were cultured in a 12-well plate for 24 hours prior to the experiment. On the day of transfection, the complexes between 0.75 μL Xfect transfection reagent and 2.5 μg plasmids [1.25 μg pHSV plasmids encoding the protein target and 1.25 μg pcDNA3.1(+) plasmids encoding the EV or the degrader] were mixed in 50 μL Xfect reaction buffer for 10 min. The media were replaced with 500 μL fresh complete DMEM medium and the Xfect-plasmid mixture was added dropwise into the well to incubate at 37 °C for 24 hours. Next, the cells were washed once with cold PBS and detached by trypsin. The RNA extraction was conducted using the Quick-RNA Miniprep Plus Kit (Zymo Research, Cat# R1057) following the manufacturer's protocol. The extracted RNA was quantified by a microvolume spectrophotometer (Thermo Scientific NanoDrop One/One^{c}). Next, reverse transcription was carried out (Zymo Research, Cat# R3012) using a same amount of RNA from each group to obtain cDNA as the template for qPCR. For qPCR, the RT-product was diluted 10 times as the cDNA template. TaqMan probes and primers (details below) were ordered from Integrated DNA Technologies. The real-time PCR was performed on a QuantStudio 5 Real-Time PCR System (Applied Biosystems) with Luna Universal Probe qPCR mastermix (NEB, Cat# M3004X) using the manufacturer's recommended protocol. Four technical replicates were carried out and the resulted average C_q value was used as one biological replicate. For each experiment, three biological replicates of RT-qPCR analysis were performed.

List of primers and TaqMan probes:

Forward primer for ALFA-tagged HTT-103Q: 5'-TGGCGGCTGTTCAAGAAGAT-3';

Reverse primer for ALFA-tagged HTT-103Q: 5'-CTTTTGAGGGACTCGAAGGC-3';

Probe for ALFA-tagged HTT-103Q: 5'-TCCGGTATGGCGACCCTGGAAA-3' (5'FAM/ZEN/3'IBFQ format);

Forward primer for ALFA-tagged α-synuclein: 5'-ACAACGAGGCCTACGAGAT-3';

Reverse primer for ALFA-tagged α-synuclein: 5'-TCAGTCTTCTTCTCAGCTCCTC-3';

Probe for ALFA-tagged α-synuclein: 5'-CGAAGAGGGCTACCAGGACTACGA-3' (5'FAM/ZEN/3'IBFQ format);

Forward primer for HPRT1 reference gene: 5'-GCGATGTCAATAGGACTCCAG-3';

Reverse primer for HPRT1 reference gene: 5'-TTGTTGTAGGATATGCCCTTGA-3';

Probe for HPRT1 reference gene: 5'-AGCCTAAGATGAGAGTTCAAGTTGAGTTTGG-3' (5'Cy5/TAO/3'IBRQ format).

2.13. Doxycycline-inducible expression for AceTAC degraders

The U2OS cells for doxycycline (Dox)-related experiments were cultured and passaged in DMEM (ThermoFisher, Cat# 10567014) supplemented with 10% tetracycline-negative FBS (GeminiBio, Cat# 100-800). The HiBiT luminescence assay was conducted following the Section "2.3. HiBiT luminescence assay for the quantification of targeted protein degradation". Instead of the pcDNA3.1(+) vector, the Tet-On 3G vectors for Dox-inducible expression of AceTAC degraders were co-transfected with the HTT-103Q-encoding plasmid. Varied concentrations of Dox (Sigma-Aldrich, Cat# D9891) were presented at the start of the transfection process. After the plasmid-transfection into U2OS cells for 24 hours, the plasmid-Xfect complex-containing media were replaced with fresh DMEM growth medium that contained the corresponding concentration of Dox. The cells were further incubated 6 hours at 37 °C, followed by the HiBiT luminescence assay readout. After subtracting the value of the assay background (non-transfected U2OS cells without Dox-treatment), the luminescence value of the EV/HTT-103Q group without Dox-treatment was normalized as 100%.

3. Supplementary sequences

3.1. Degraders

p62FL: HA-tagged on the C-terminus; Vector backbone: pcDNA3.1(+)

MASLTVKAYLLGKEDAAREIRRFSFCCSPEPEAEAEAAAGPGPCERLLSRVAALFPALRPGGFQAHYRD
EDGDLVAFSSDEELTMAMSYVKDDIFRIYIKEKKECRRDHRPPCAQEAPRNMVHPNVICDGCNGPVVGT
RYKCSVCPDYDLCSVCEGKGLHRGHTKLAFPSPFGHLSEGFSHSRWLRKVKHGHFGWPGWEMGPPGNWS
PRPPRAGEARPGPTAESASGPSEDPSVNFLKNVGESVAAALSPLGIEVDIDVEHGGKRSRLTPVSPESS
STEEKSSSQPSSCCSDPSKPGGNVEGATQSLAEQMRKIALESEGRPEEQMESDNCSGGDDDWTHLSSKE
VDPSTGELQSLQMPESEGPSSLDPSQEGPTGLKEAALYPHLPPEADPRLIESLSQMLSMGFSDEGGWLT
RLLQTKNYDIGAALDTIQYSKHPPPLRGRYPYDVPDYA*

p62FL-A1: p62FL—Ab; Vector backbone: pcDNA3.1(+)

MASLTVKAYLLGKEDAAREIRRFSFCCSPEPEAEAEAAAGPGPCERLLSRVAALFPALRPGGFQAHYRD
EDGDLVAFSSDEELTMAMSYVKDDIFRIYIKEKKECRRDHRPPCAQEAPRNMVHPNVICDGCNGPVVGT
RYKCSVCPDYDLCSVCEGKGLHRGHTKLAFPSPFGHLSEGFSHSRWLRKVKHGHFGWPGWEMGPPGNWS
PRPPRAGEARPGPTAESASGPSEDPSVNFLKNVGESVAAALSPLGIEVDIDVEHGGKRSRLTPVSPESS
STEEKSSSQPSSCCSDPSKPGGNVEGATQSLAEQMRKIALESEGRPEEQMESDNCSGGDDDWTHLSSKE
VDPSTGELQSLQMPESEGPSSLDPSQEGPTGLKEAALYPHLPPEADPRLIESLSQMLSMGFSDEGGWLT
RLLQTKNYDIGAALDTIQYSKHPPPLRGRSGGSSGGSSGSETPGTSESATPESSGGSSGGSMEVQLQES
GGGLVQPGGSLRLSCTASGVTISALNAMAMGWYRQAPGERRVMVAAVSERGNAMYRESVQGRFTVTRDF
TNKMVSLQMDNLKPEDTAVYYCHVLEDRVDSFHDYWGQGTQVTVSSRGRYPYDVPDYA*

p62FL-A2: p62FL—Ab—Ab; Vector backbone: pcDNA3.1(+)

MASLTVKAYLLGKEDAAREIRRFSFCCSPEPEAEAEAAAGPGPCERLLSRVAALFPALRPGGFQAHYRD
EDGDLVAFSSDEELTMAMSYVKDDIFRIYIKEKKECRRDHRPPCAQEAPRNMVHPNVICDGCNGPVVGT
RYKCSVCPDYDLCSVCEGKGLHRGHTKLAFPSPFGHLSEGFSHSRWLRKVKHGHFGWPGWEMGPPGNWS
PRPPRAGEARPGPTAESASGPSEDPSVNFLKNVGESVAAALSPLGIEVDIDVEHGGKRSRLTPVSPESS
STEEKSSSQPSSCCSDPSKPGGNVEGATQSLAEQMRKIALESEGRPEEQMESDNCSGGDDDWTHLSSKE
VDPSTGELQSLQMPESEGPSSLDPSQEGPTGLKEAALYPHLPPEADPRLIESLSQMLSMGFSDEGGWLT
RLLQTKNYDIGAALDTIQYSKHPPPLSGGSSGGSSGSETPGTSESATPESSGGSSGGSMEVQLQESGGG
LVQPGGSLRLSCTASGVTISALNAMAMGWYRQAPGERRVMVAAVSERGNAMYRESVQGRFTVTRDFTNK
MVSLQMDNLKPEDTAVYYCHVLEDRVDSFHDYWGQGTQVTVSSSGGSSGGSSGSETPGTSESATPESSG
GSSGGSMEVQLQESGGGLVQPGGSLRLSCTASGVTISALNAMAMGWYRQAPGERRVMVAAVSERGNAMY
RESVQGRFTVTRDFTNKMVSLQMDNLKPEDTAVYYCHVLEDRVDSFHDYWGQGTQVTVSSRGRYPYDVP
DYA*

p62FL-A3: p62FL—Ab—Ab—Ab; Vector backbone: pcDNA3.1(+) or Tet-On 3G

MASLTVKAYLLGKEDAAREIRRFSFCCSPEPEAEAEAAAGPGPCERLLSRVAALFPALRPGGFQAHYRD
EDGDLVAFSSDEELTMAMSYVKDDIFRIYIKEKKECRRDHRPPCAQEAPRNMVHPNVICDGCNGPVVGT
RYKCSVCPDYDLCSVCEGKGLHRGHTKLAFPSPFGHLSEGFSHSRWLRKVKHGHFGWPGWEMGPPGNWS
PRPPRAGEARPGPTAESASGPSEDPSVNFLKNVGESVAAALSPLGIEVDIDVEHGGKRSRLTPVSPESS
STEEKSSSQPSSCCSDPSKPGGNVEGATQSLAEQMRKIALESEGRPEEQMESDNCSGGDDDWTHLSSKE
VDPSTGELQSLQMPESEGPSSLDPSQEGPTGLKEAALYPHLPPEADPRLIESLSQMLSMGFSDEGGWLT
RLLQTKNYDIGAALDTIQYSKHPPPLSGGSSGGSSGSETPGTSESATPESSGGSSGGSMEVQLQESGGG
LVQPGGSLRLSCTASGVTISALNAMAMGWYRQAPGERRVMVAAVSERGNAMYRESVQGRFTVTRDFTNK
MVSLQMDNLKPEDTAVYYCHVLEDRVDSFHDYWGQGTQVTVSSSGGSSGGSSGSETPGTSESATPESSG
GSSGGSMEVQLQESGGGLVQPGGSLRLSCTASGVTISALNAMAMGWYRQAPGERRVMVAAVSERGNAMY
RESVQGRFTVTRDFTNKMVSLQMDNLKPEDTAVYYCHVLEDRVDSFHDYWGQGTQVTVSSSGGSSGGSS
GSETPGTSESATPESSGGSSGGSMEVQLQESGGGLVQPGGSLRLSCTASGVTISALNAMAMGWYRQAPG
ERRVMVAAVSERGNAMYRESVQGRFTVTRDFTNKMVSLQMDNLKPEDTAVYYCHVLEDRVDSFHDYWGQ
GTQVTVSSRGRYPYDVPDYA*

p62FL-A3$^{\Delta PB1}$: p62ΔPB1—Ab—Ab—Ab; Vector backbone: pcDNA3.1(+)

MAKECRRDHRPPCAQEAPRNMVHPNVICDGCNGPVVGTRYKCSVCPDYDLCSVCEGKGLHRGHTKLAFP
SPFGHLSEGFSHSRWLRKVKHGHFGWPGWEMGPPGNWSPRPPRAGEARPGPTAESASGPSEDPSVNFLK
NVGESVAAALSPLGIEVDIDVEHGGKRSRLTPVSPESSSTEEKSSSQPSSCCSDPSKPGGNVEGATQSL
AEQMRKIALESEGRPEEQMESDNCSGGDDDWTHLSSKEVDPSTGELQSLQMPESEGPSSLDPSQEGPTG
LKEAALYPHLPPEADPRLIESLSQMLSMGFSDEGGWLTRLLQTKNYDIGAALDTIQYSKHPPPLSGGSS
GGSSGSETPGTSESATPESSGGSSGGSMEVQLQESGGGLVQPGGSLRLSCTASGVTISALNAMAMGWYR
QAPGERRVMVAAVSERGNAMYRESVQGRFTVTRDFTNKMVSLQMDNLKPEDTAVYYCHVLEDRVDSFHD
YWGQGTQVTVSSSGGSSGGSSGSETPGTSESATPESSGGSSGGSMEVQLQESGGGLVQPGGSLRLSCTA
SGVTISALNAMAMGWYRQAPGERRVMVAAVSERGNAMYRESVQGRFTVTRDFTNKMVSLQMDNLKPEDT
AVYYCHVLEDRVDSFHDYWGQGTQVTVSSSGGSSGGSSGSETPGTSESATPESSGGSSGGSMEVQLQES
GGGLVQPGGSLRLSCTASGVTISALNAMAMGWYRQAPGERRVMVAAVSERGNAMYRESVQGRFTVTRDF
TNKMVSLQMDNLKPEDTAVYYCHVLEDRVDSFHDYWGQGTQVTVSSRGRYPYDVPDYA*

p62FL-A3$^{\Delta ZZ}$: p62ΔZZ—Ab—Ab—Ab; Vector backbone: pcDNA3.1(+)

MASLTVKAYLLGKEDAAREIRRFSFCCSPEPEAEAEAAAGPGPCERLLSRVAALFPALRPGGFQAHYRD
EDGDLVAFSSDEELTMAMSYVKDDIFRIYIKEKKECRRDHRPPCAQEAPRNMFPSPFGHLSEGFSHSRW
LRKVKHGHFGWPGWEMGPPGNWSPRPPRAGEARPGPTAESASGPSEDPSVNFLKNVGESVAAALSPLGI
EVDIDVEHGGKRSRLTPVSPESSSTEEKSSSQPSSCCSDPSKPGGNVEGATQSLAEQMRKIALESEGRP
EEQMESDNCSGGDDDWTHLSSKEVDPSTGELQSLQMPESEGPSSLDPSQEGPTGLKEAALYPHLPPEAD
PRLIESLSQMLSMGFSDEGGWLTRLLQTKNYDIGAALDTIQYSKHPPPLSGGSSGGSSGSETPGTSESA
TPESSGGSSGGSMEVQLQESGGGLVQPGGSLRLSCTASGVTISALNAMAMGWYRQAPGERRVMVAAVSE
RGNAMYRESVQGRFTVTRDFTNKMVSLQMDNLKPEDTAVYYCHVLEDRVDSFHDYWGQGTQVTVSSSGG
SSGGSSGSETPGTSESATPESSGGSSGGSMEVQLQESGGGLVQPGGSLRLSCTASGVTISALNAMAMGW
YRQAPGERRVMVAAVSERGNAMYRESVQGRFTVTRDFTNKMVSLQMDNLKPEDTAVYYCHVLEDRVDSF
HDYWGQGTQVTVSSSGGSSGGSSGSETPGTSESATPESSGGSSGGSMEVQLQESGGGLVQPGGSLRLSC
TASGVTISALNAMAMGWYRQAPGERRVMVAAVSERGNAMYRESVQGRFTVTRDFTNKMVSLQMDNLKPE
DTAVYYCHVLEDRVDSFHDYWGQGTQVTVSSRGRYPYDVPDYA*

p62FL-A3ΔLIR: p62ΔLIR—Ab—Ab—Ab; Vector backbone: pcDNA3.1(+)

MASLTVKAYLLGKEDAAREIRRFSFCCSPEPEAEAEAAAGPGPCERLLSRVAALFPALRPGGFQAHYRD
EDGDLVAFSSDEELTMAMSYVKDDIFRIYIKEKKECRRDHRPPCAQEAPRNMVHPNVICDGCNGPVVGT
RYKCSVCPDYDLCSVCEGKGLHRGHTKLAFPSPFGHLSEGFSHSRWLRKVKHGHFGWPGWEMGPPGNWS
PRPPRAGEARPGPTAESASGPSEDPSVNFLKNVGESVAAALSPLGIEVDIDVEHGGKRSRLTPVSPESS
STEEKSSSQPSSCCSDPSKPGGNVEGATQSLAEQMRKIALESEGSKEVDPSTGELQSLQMPESEGPSSL
DPSQEGPTGLKEAALYPHLPPEADPRLIESLSQMLSMGFSDEGGWLTRLLQTKNYDIGAALDTIQYSKH
PPPLSGGSSGGSSGSETPGTSESATPESSGGSSGGSMEVQLQESGGGLVQPGGSLRLSCTASGVTISAL
NAMAMGWYRQAPGERRVMVAAVSERGNAMYRESVQGRFTVTRDFTNKMVSLQMDNLKPEDTAVYYCHVL
EDRVDSFHDYWGQGTQVTVSSSGGSSGGSSGSETPGTSESATPESSGGSSGGSMEVQLQESGGGLVQPG
GSLRLSCTASGVTISALNAMAMGWYRQAPGERRVMVAAVSERGNAMYRESVQGRFTVTRDFTNKMVSLQ
MDNLKPEDTAVYYCHVLEDRVDSFHDYWGQGTQVTVSSSGGSSGGSSGSETPGTSESATPESSGGSSGG
SMEVQLQESGGGLVQPGGSLRLSCTASGVTISALNAMAMGWYRQAPGERRVMVAAVSERGNAMYRESVQ
GRFTVTRDFTNKMVSLQMDNLKPEDTAVYYCHVLEDRVDSFHDYWGQGTQVTVSSRGRYPYDVPDYA*

p62FL-A3ΔNES: p62ΔNES—Ab—Ab—Ab; Vector backbone: pcDNA3.1(+)

MASLTVKAYLLGKEDAAREIRRFSFCCSPEPEAEAEAAAGPGPCERLLSRVAALFPALRPGGFQAHYRD
EDGDLVAFSSDEELTMAMSYVKDDIFRIYIKEKKECRRDHRPPCAQEAPRNMVHPNVICDGCNGPVVGT
RYKCSVCPDYDLCSVCEGKGLHRGHTKLAFPSPFGHLSEGFSHSRWLRKVKHGHFGWPGWEMGPPGNWS
PRPPRAGEARPGPTAESASGPSEDPSVNFLKNVGESVAAALSPLGIEVDIDVEHGGKRSRLTPVSPESS
STEEKSSSQPSSCCSDPSKPGGNVEGRPEEQMESDNCSGGDDDWTHLSSKEVDPSTGELQSLQMPESEG
PSSLDPSQEGPTGLKEAALYPHLPPEADPRLIESLSQMLSMGFSDEGGWLTRLLQTKNYDIGAALDTIQ
YSKHPPPLSGGSSGGSSGSETPGTSESATPESSGGSSGGSMEVQLQESGGGLVQPGGSLRLSCTASGVT
ISALNAMAMGWYRQAPGERRVMVAAVSERGNAMYRESVQGRFTVTRDFTNKMVSLQMDNLKPEDTAVYY
CHVLEDRVDSFHDYWGQGTQVTVSSSGGSSGGSSGSETPGTSESATPESSGGSSGGSMEVQLQESGGGL
VQPGGSLRLSCTASGVTISALNAMAMGWYRQAPGERRVMVAAVSERGNAMYRESVQGRFTVTRDFTNKM
VSLQMDNLKPEDTAVYYCHVLEDRVDSFHDYWGQGTQVTVSSSGGSSGGSSGSETPGTSESATPESSGG
SSGGSMEVQLQESGGGLVQPGGSLRLSCTASGVTISALNAMAMGWYRQAPGERRVMVAAVSERGNAMYR
ESVQGRFTVTRDFTNKMVSLQMDNLKPEDTAVYYCHVLEDRVDSFHDYWGQGTQVTVSSRGRYPYDVPD
YA*

p62FL-A3ΔUBA: p62ΔUBA—Ab—Ab—Ab; Vector backbone: pcDNA3.1(+)

MASLTVKAYLLGKEDAAREIRRFSFCCSPEPEAEAEAAAGPGPCERLLSRVAALFPALRPGGFQAHYRD
EDGDLVAFSSDEELTMAMSYVKDDIFRIYIKEKKECRRDHRPPCAQEAPRNMVHPNVICDGCNGPVVGT
RYKCSVCPDYDLCSVCEGKGLHRGHTKLAFPSPFGHLSEGFSHSRWLRKVKHGHFGWPGWEMGPPGNWS
PRPPRAGEARPGPTAESASGPSEDPSVNFLKNVGESVAAALSPLGIEVDIDVEHGGKRSRLTPVSPESS
STEEKSSSQPSSCCSDPSKPGGNVEGATQSLAEQMRKIALESEGRPEEQMESDNCSGGDDDWTHLSSKE
VDPSTGELQSLQMPESEGPSSLDPSQEGPTGLKEAALYPHSGGSSGGSSGSETPGTSESATPESSGGSS
GGSMEVQLQESGGGLVQPGGSLRLSCTASGVTISALNAMAMGWYRQAPGERRVMVAAVSERGNAMYRES
VQGRFTVTRDFTNKMVSLQMDNLKPEDTAVYYCHVLEDRVDSFHDYWGQGTQVTVSSSGGSSGGSSGSE

TPGTSESATPESSGGSSGGSMEVQLQESGGGLVQPGGSLRLSCTASGVTISALNAMAMGWYRQAPGERR
VMVAAVSERGNAMYRESVQGRFTVTRDFTNKMVSLQMDNLKPEDTAVYYCHVLEDRVDSFHDYWGQGTQ
VTVSSSGGSSGGSSGSETPGTSESATPESSGGSSGGSMEVQLQESGGGLVQPGGSLRLSCTASGVTISA
LNAMAMGWYRQAPGERRVMVAAVSERGNAMYRESVQGRFTVTRDFTNKMVSLQMDNLKPEDTAVYYCHV
LEDRVDSFHDYWGQGTQVTVSSRGRYPYDVPDYA*

p62FL-A3/TP53INP2(LIR): p62FL/TP53INP2(LIR)—Ab—Ab—Ab; Vector backbone: pcDNA3.1(+)

MASLTVKAYLLGKEDAAREIRRFSFCCSPEPEAEAEAAAGPGPCERLLSRVAALFPALRPGGFQAHYRD
EDGDLVAFSSDEELTMAMSYVKDDIFRIYIKEKKECRRDHRPPCAQEAPRNMVHPNVICDGCNGPVVGT
RYKCSVCPDYDLCSVCEGKGLHRGHTKLAFPSPFGHLSEGFSHSRWLRKVKHGHFGWPGWEMGPPGNWS
PRPPRAGEARPGPTAESASGPSEDPSVNFLKNVGESVAAALSPLGIEVDIDVEHGGKRSRLTPVSPESS
STEEKSSSQPSSCCSDPSKPGGNVEGATQSLAEQMRKIALESEGFVSEEDEVDGWLIIDLPDSYAAPPS
SKEVDPSTGELQSLQMPESEGPSSLDPSQEGPTGLKEAALYPHLPPEADPRLIESLSQMLSMGFSDEGG
WLTRLLQTKNYDIGAALDTIQYSKHPPPLSGGSSGGSSGSETPGTSESATPESSGGSSGGSMEVQLQES
GGGLVQPGGSLRLSCTASGVTISALNAMAMGWYRQAPGERRVMVAAVSERGNAMYRESVQGRFTVTRDF
TNKMVSLQMDNLKPEDTAVYYCHVLEDRVDSFHDYWGQGTQVTVSSSGGSSGGSSGSETPGTSESATPE
SSGGSSGGSMEVQLQESGGGLVQPGGSLRLSCTASGVTISALNAMAMGWYRQAPGERRVMVAAVSERGN
AMYRESVQGRFTVTRDFTNKMVSLQMDNLKPEDTAVYYCHVLEDRVDSFHDYWGQGTQVTVSSSGGSSG
GSSGSETPGTSESATPESSGGSSGGSMEVQLQESGGGLVQPGGSLRLSCTASGVTISALNAMAMGWYRQ
APGERRVMVAAVSERGNAMYRESVQGRFTVTRDFTNKMVSLQMDNLKPEDTAVYYCHVLEDRVDSFHDY
WGQGTQVTVSSRGRYPYDVPDYA*

p62FL-A3/TBC1D25(LIR): p62FL/TBC1D25(LIR)—Ab—Ab—Ab; Vector backbone: pcDNA3.1(+)

MASLTVKAYLLGKEDAAREIRRFSFCCSPEPEAEAEAAAGPGPCERLLSRVAALFPALRPGGFQAHYRD
EDGDLVAFSSDEELTMAMSYVKDDIFRIYIKEKKECRRDHRPPCAQEAPRNMVHPNVICDGCNGPVVGT
RYKCSVCPDYDLCSVCEGKGLHRGHTKLAFPSPFGHLSEGFSHSRWLRKVKHGHFGWPGWEMGPPGNWS
PRPPRAGEARPGPTAESASGPSEDPSVNFLKNVGESVAAALSPLGIEVDIDVEHGGKRSRLTPVSPESS
STEEKSSSQPSSCCSDPSKPGGNVEGATQSLAEQMRKIALESEGPSEDSPLLEDWDIISPKDVIGSDVL
SKEVDPSTGELQSLQMPESEGPSSLDPSQEGPTGLKEAALYPHLPPEADPRLIESLSQMLSMGFSDEGG
WLTRLLQTKNYDIGAALDTIQYSKHPPPLSGGSSGGSSGSETPGTSESATPESSGGSSGGSMEVQLQES
GGGLVQPGGSLRLSCTASGVTISALNAMAMGWYRQAPGERRVMVAAVSERGNAMYRESVQGRFTVTRDF
TNKMVSLQMDNLKPEDTAVYYCHVLEDRVDSFHDYWGQGTQVTVSSSGGSSGGSSGSETPGTSESATPE
SSGGSSGGSMEVQLQESGGGLVQPGGSLRLSCTASGVTISALNAMAMGWYRQAPGERRVMVAAVSERGN
AMYRESVQGRFTVTRDFTNKMVSLQMDNLKPEDTAVYYCHVLEDRVDSFHDYWGQGTQVTVSSSGGSSG
GSSGSETPGTSESATPESSGGSSGGSMEVQLQESGGGLVQPGGSLRLSCTASGVTISALNAMAMGWYRQ
APGERRVMVAAVSERGNAMYRESVQGRFTVTRDFTNKMVSLQMDNLKPEDTAVYYCHVLEDRVDSFHDY
WGQGTQVTVSSRGRYPYDVPDYA*

p62FL-A3/FYCO1(LIR): p62FL/FYCO1(LIR)—Ab—Ab—Ab; Vector backbone: pcDNA3.1(+)

MASLTVKAYLLGKEDAAREIRRFSFCCSPEPEAEAEAAAGPGPCERLLSRVAALFPALRPGGFQAHYRD
EDGDLVAFSSDEELTMAMSYVKDDIFRIYIKEKKECRRDHRPPCAQEAPRNMVHPNVICDGCNGPVVGT

RYKCSVCPDYDLCSVCEGKGLHRGHTKLAFPSPFGHLSEGFSHSRWLRKVKHGHFGWPGWEMGPPGNWS
PRPPRAGEARPGPTAESASGPSEDPSVNFLKNVGESVAAALSPLGIEVDIDVEHGGKRSRLTPVSPESS
STEEKSSSQPSSCCSDPSKPGGNVEGATQSLAEQMRKIALESEGRPPDDAVFDIITDEELCQIQESGSS
KEVDPSTGELQSLQMPESEGPSSLDPSQEGPTGLKEAALYPHLPPEADPRLIESLSQMLSMGFSDEGGW
LTRLLQTKNYDIGAALDTIQYSKHPPPLSGGSSGGSSGSETPGTSESATPESSGGSSGGSMEVQLQESG
GGLVQPGGSLRLSCTASGVTISALNAMAMGWYRQAPGERRVMVAAVSERGNAMYRESVQGRFTVTRDFT
NKMVSLQMDNLKPEDTAVYYCHVLEDRVDSFHDYWGQGTQVTVSSSGGSSGGSSGSETPGTSESATPES
SGGSSGGSMEVQLQESGGGLVQPGGSLRLSCTASGVTISALNAMAMGWYRQAPGERRVMVAAVSERGNA
MYRESVQGRFTVTRDFTNKMVSLQMDNLKPEDTAVYYCHVLEDRVDSFHDYWGQGTQVTVSSSGGSSGG
SSGSETPGTSESATPESSGGSSGGSMEVQLQESGGGLVQPGGSLRLSCTASGVTISALNAMAMGWYRQA
PGERRVMVAAVSERGNAMYRESVQGRFTVTRDFTNKMVSLQMDNLKPEDTAVYYCHVLEDRVDSFHDYW
GQGTQVTVSSRGRYPYDVPDYA*

T1A1: p62ΔUBA—LIR(TP53INP2)—Ab; Vector backbone: pcDNA3.1(+)

MASLTVKAYLLGKEDAAREIRRFSFCCSPEPEAEAEAAAGPGPCERLLSRVAALFPALRPGGFQAHYRD
EDGDLVAFSSDEELTMAMSYVKDDIFRIYIKEKKECRRDHRPPCAQEAPRNMVHPNVICDGCNGPVVGT
RYKCSVCPDYDLCSVCEGKGLHRGHTKLAFPSPFGHLSEGFSHSRWLRKVKHGHFGWPGWEMGPPGNWS
PRPPRAGEARPGPTAESASGPSEDPSVNFLKNVGESVAAALSPLGIEVDIDVEHGGKRSRLTPVSPESS
STEEKSSSQPSSCCSDPSKPGGNVEGATQSLAEQMRKIALESEGFVSEEDEVDGWLIIDLPDSYAAPPS
SKEVDPSTGELQSLQMPESEGPSSLDPSQEGPTGLKEAALYPHSGGSSGGSSGSETPGTSESATPESSG
GSSGGSMEVQLQESGGGLVQPGGSLRLSCTASGVTISALNAMAMGWYRQAPGERRVMVAAVSERGNAMY
RESVQGRFTVTRDFTNKMVSLQMDNLKPEDTAVYYCHVLEDRVDSFHDYWGQGTQVTVSSRGRYPYDVP
DYA*

T1A2: p62ΔUBA—LIR(TP53INP2)—Ab—Ab; Vector backbone: pcDNA3.1(+)

MASLTVKAYLLGKEDAAREIRRFSFCCSPEPEAEAEAAAGPGPCERLLSRVAALFPALRPGGFQAHYRD
EDGDLVAFSSDEELTMAMSYVKDDIFRIYIKEKKECRRDHRPPCAQEAPRNMVHPNVICDGCNGPVVGT
RYKCSVCPDYDLCSVCEGKGLHRGHTKLAFPSPFGHLSEGFSHSRWLRKVKHGHFGWPGWEMGPPGNWS
PRPPRAGEARPGPTAESASGPSEDPSVNFLKNVGESVAAALSPLGIEVDIDVEHGGKRSRLTPVSPESS
STEEKSSSQPSSCCSDPSKPGGNVEGATQSLAEQMRKIALESEGFVSEEDEVDGWLIIDLPDSYAAPPS
SKEVDPSTGELQSLQMPESEGPSSLDPSQEGPTGLKEAALYPHSGGSSGGSSGSETPGTSESATPESSG
GSSGGSMEVQLQESGGGLVQPGGSLRLSCTASGVTISALNAMAMGWYRQAPGERRVMVAAVSERGNAMY
RESVQGRFTVTRDFTNKMVSLQMDNLKPEDTAVYYCHVLEDRVDSFHDYWGQGTQVTVSSSGGSSGGSS
GSETPGTSESATPESSGGSSGGSMEVQLQESGGGLVQPGGSLRLSCTASGVTISALNAMAMGWYRQAPG
ERRVMVAAVSERGNAMYRESVQGRFTVTRDFTNKMVSLQMDNLKPEDTAVYYCHVLEDRVDSFHDYWGQ
GTQVTVSSRGRYPYDVPDYA*

T2A2: p62ΔUBA—LIR(TP53INP2)—LIR(TP53INP2)—Ab—Ab; Vector backbone: pcDNA3.1(+)

MASLTVKAYLLGKEDAAREIRRFSFCCSPEPEAEAEAAAGPGPCERLLSRVAALFPALRPGGFQAHYRD
EDGDLVAFSSDEELTMAMSYVKDDIFRIYIKEKKECRRDHRPPCAQEAPRNMVHPNVICDGCNGPVVGT
RYKCSVCPDYDLCSVCEGKGLHRGHTKLAFPSPFGHLSEGFSHSRWLRKVKHGHFGWPGWEMGPPGNWS

PRPPRAGEARPGPTAESASGPSEDPSVNFLKNVGESVAAALSPLGIEVDIDVEHGGKRSRLTPVSPESS
STEEKSSSQPSSCCSDPSKPGGNVEGATQSLAEQMRKIALESEGFVSEEDEVDGWLIIDLPDSYAAPPS
SGGSSGGSSGSETPGTSESATPESSGGSSGGSFVSEEDEVDGWLIIDLPDSYAAPPSSKEVDPSTGELQ
SLQMPESEGPSSLDPSQEGPTGLKEAALYPHSGGSSGGSSGSETPGTSESATPESSGGSSGGSMEVQLQ
ESGGGLVQPGGSLRLSCTASGVTISALNAMAMGWYRQAPGERRVMVAAVSERGNAMYRESVQGRFTVTR
DFTNKMVSLQMDNLKPEDTAVYYCHVLEDRVDSFHDYWGQGTQVTVSSSGGSSGGSSGSETPGTSESAT
PESSGGSSGGSMEVQLQESGGGLVQPGGSLRLSCTASGVTISALNAMAMGWYRQAPGERRVMVAAVSER
GNAMYRESVQGRFTVTRDFTNKMVSLQMDNLKPEDTAVYYCHVLEDRVDSFHDYWGQGTQVTVSSRGRY
PYDVPDYA*

T1A3: p62ΔUBA—LIR(TP53INP2)—Ab—Ab—Ab; Vector backbone: pcDNA3.1(+)

MASLTVKAYLLGKEDAAREIRRFSFCCSPEPEAEAEAAAGPGPCERLLSRVAALFPALRPGGFQAHYRD
EDGDLVAFSSDEELTMAMSYVKDDIFRIYIKEKKECRRDHRPPCAQEAPRNMVHPNVICDGCNGPVVGT
RYKCSVCPDYDLCSVCEGKGLHRGHTKLAFPSPFGHLSEGFSHSRWLRKVKHGHFGWPGWEMGPPGNWS
PRPPRAGEARPGPTAESASGPSEDPSVNFLKNVGESVAAALSPLGIEVDIDVEHGGKRSRLTPVSPESS
STEEKSSSQPSSCCSDPSKPGGNVEGATQSLAEQMRKIALESEGFVSEEDEVDGWLIIDLPDSYAAPPS
SKEVDPSTGELQSLQMPESEGPSSLDPSQEGPTGLKEAALYPHSGGSSGGSSGSETPGTSESATPESSG
GSSGGSMEVQLQESGGGLVQPGGSLRLSCTASGVTISALNAMAMGWYRQAPGERRVMVAAVSERGNAMY
RESVQGRFTVTRDFTNKMVSLQMDNLKPEDTAVYYCHVLEDRVDSFHDYWGQGTQVTVSSSGGSSGGSS
GSETPGTSESATPESSGGSSGGSMEVQLQESGGGLVQPGGSLRLSCTASGVTISALNAMAMGWYRQAPG
ERRVMVAAVSERGNAMYRESVQGRFTVTRDFTNKMVSLQMDNLKPEDTAVYYCHVLEDRVDSFHDYWGQ
GTQVTVSSSGGSSGGSSGSETPGTSESATPESSGGSSGGSMEVQLQESGGGLVQPGGSLRLSCTASGVT
ISALNAMAMGWYRQAPGERRVMVAAVSERGNAMYRESVQGRFTVTRDFTNKMVSLQMDNLKPEDTAVYY
CHVLEDRVDSFHDYWGQGTQVTVSSRGRYPYDVPDYA*

T2A3: p62ΔUBA—LIR(TP53INP2)—LIR(TP53INP2)—Ab—Ab—Ab; Vector backbone: pcDNA3.1(+)

MASLTVKAYLLGKEDAAREIRRFSFCCSPEPEAEAEAAAGPGPCERLLSRVAALFPALRPGGFQAHYRD
EDGDLVAFSSDEELTMAMSYVKDDIFRIYIKEKKECRRDHRPPCAQEAPRNMVHPNVICDGCNGPVVGT
RYKCSVCPDYDLCSVCEGKGLHRGHTKLAFPSPFGHLSEGFSHSRWLRKVKHGHFGWPGWEMGPPGNWS
PRPPRAGEARPGPTAESASGPSEDPSVNFLKNVGESVAAALSPLGIEVDIDVEHGGKRSRLTPVSPESS
STEEKSSSQPSSCCSDPSKPGGNVEGATQSLAEQMRKIALESEGFVSEEDEVDGWLIIDLPDSYAAPPS
SGGSSGGSSGSETPGTSESATPESSGGSSGGSFVSEEDEVDGWLIIDLPDSYAAPPSSKEVDPSTGELQ
SLQMPESEGPSSLDPSQEGPTGLKEAALYPHSGGSSGGSSGSETPGTSESATPESSGGSSGGSMEVQLQ
ESGGGLVQPGGSLRLSCTASGVTISALNAMAMGWYRQAPGERRVMVAAVSERGNAMYRESVQGRFTVTR
DFTNKMVSLQMDNLKPEDTAVYYCHVLEDRVDSFHDYWGQGTQVTVSSSGGSSGGSSGSETPGTSESAT
PESSGGSSGGSMEVQLQESGGGLVQPGGSLRLSCTASGVTISALNAMAMGWYRQAPGERRVMVAAVSER
GNAMYRESVQGRFTVTRDFTNKMVSLQMDNLKPEDTAVYYCHVLEDRVDSFHDYWGQGTQVTVSSSGGS
SGGSSGSETPGTSESATPESSGGSSGGSMEVQLQESGGGLVQPGGSLRLSCTASGVTISALNAMAMGWY
RQAPGERRVMVAAVSERGNAMYRESVQGRFTVTRDFTNKMVSLQMDNLKPEDTAVYYCHVLEDRVDSFH
DYWGQGTQVTVSSRGRYPYDVPDYA*

T3A3: p62ΔUBA—LIR(TP53INP2)—LIR(TP53INP2)—LIR(TP53INP2)—Ab—Ab—Ab; Vector backbone:

pcDNA3.1(+) or Tet-On 3G

MASLTVKAYLLGKEDAAREIRRFSFCCSPEPEAEAEAAAGPGPCERLLSRVAALFPALRPGGFQAHYRD
EDGDLVAFSSDEELTMAMSYVKDDIFRIYIKEKKECRRDHRPPCAQEAPRNMVHPNVICDGCNGPVVGT
RYKCSVCPDYDLCSVCEGKGLHRGHTKLAFPSPFGHLSEGFSHSRWLRKVKHGHFGWPGWEMGPPGNWS
PRPPRAGEARPGPTAESASGPSEDPSVNFLKNVGESVAAALSPLGIEVDIDVEHGGKRSRLTPVSPESS
STEEKSSSQPSSCCSDPSKPGGNVEGATQSLAEQMRKIALESEGFVSEEDEVDGWLIIDLPDSYAAPPS
SGGSSGGSSGSETPGTSESATPESSGGSSGGSFVSEEDEVDGWLIIDLPDSYAAPPSSGGSSGGSSGSE
TPGTSESATPESSGGSSGGSFVSEEDEVDGWLIIDLPDSYAAPPSSKEVDPSTGELQSLQMPESEGPSS
LDPSQEGPTGLKEAALYPHSGGSSGGSSGSETPGTSESATPESSGGSSGGSMEVQLQESGGGLVQPGGS
LRLSCTASGVTISALNAMAMGWYRQAPGERRVMVAAVSERGNAMYRESVQGRFTVTRDFTNKMVSLQMD
NLKPEDTAVYYCHVLEDRVDSFHDYWGQGTQVTVSSSGGSSGGSSGSETPGTSESATPESSGGSSGGSM
EVQLQESGGGLVQPGGSLRLSCTASGVTISALNAMAMGWYRQAPGERRVMVAAVSERGNAMYRESVQGR
FTVTRDFTNKMVSLQMDNLKPEDTAVYYCHVLEDRVDSFHDYWGQGTQVTVSSSGGSSGGSSGSETPGT
SESATPESSGGSSGGSMEVQLQESGGGLVQPGGSLRLSCTASGVTISALNAMAMGWYRQAPGERRVMVA
AVSERGNAMYRESVQGRFTVTRDFTNKMVSLQMDNLKPEDTAVYYCHVLEDRVDSFHDYWGQGTQVTVS
SRGRYPYDVPDYA*

T3A3$^{\Delta PB1}$: p62ΔPB1ΔUBA—LIR(TP53INP2)—LIR(TP53INP2)—LIR(TP53INP2)—Ab—Ab—Ab; Vector backbone:

pcDNA3.1(+)

MAKECRRDHRPPCAQEAPRNMVHPNVICDGCNGPVVGTRYKCSVCPDYDLCSVCEGKGLHRGHTKLAFP
SPFGHLSEGFSHSRWLRKVKHGHFGWPGWEMGPPGNWSPRPPRAGEARPGPTAESASGPSEDPSVNFLK
NVGESVAAALSPLGIEVDIDVEHGGKRSRLTPVSPESSSTEEKSSSQPSSCCSDPSKPGGNVEGATQSL
AEQMRKIALESEGFVSEEDEVDGWLIIDLPDSYAAPPSSGGSSGGSSGSETPGTSESATPESSGGSSGG
SFVSEEDEVDGWLIIDLPDSYAAPPSSGGSSGGSSGSETPGTSESATPESSGGSSGGSFVSEEDEVDGW
LIIDLPDSYAAPPSSKEVDPSTGELQSLQMPESEGPSSLDPSQEGPTGLKEAALYPHSGGSSGGSSGSE
TPGTSESATPESSGGSSGGSMEVQLQESGGGLVQPGGSLRLSCTASGVTISALNAMAMGWYRQAPGERR
VMVAAVSERGNAMYRESVQGRFTVTRDFTNKMVSLQMDNLKPEDTAVYYCHVLEDRVDSFHDYWGQGTQ
VTVSSSGGSSGGSSGSETPGTSESATPESSGGSSGGSMEVQLQESGGGLVQPGGSLRLSCTASGVTISA
LNAMAMGWYRQAPGERRVMVAAVSERGNAMYRESVQGRFTVTRDFTNKMVSLQMDNLKPEDTAVYYCHV
LEDRVDSFHDYWGQGTQVTVSSSGGSSGGSSGSETPGTSESATPESSGGSSGGSMEVQLQESGGGLVQP
GGSLRLSCTASGVTISALNAMAMGWYRQAPGERRVMVAAVSERGNAMYRESVQGRFTVTRDFTNKMVSL
QMDNLKPEDTAVYYCHVLEDRVDSFHDYWGQGTQVTVSSRGRYPYDVPDYA*

T3A3$^{\Delta PB1\Delta ZZ}$: p62ΔPB1ΔZZΔUBA—LIR(TP53INP2)—LIR(TP53INP2)—LIR(TP53INP2)—Ab—Ab—Ab; Vector

backbone: pcDNA3.1(+)

MAFPSPFGHLSEGFSHSRWLRKVKHGHFGWPGWEMGPPGNWSPRPPRAGEARPGPTAESASGPSEDPSV
NFLKNVGESVAAALSPLGIEVDIDVEHGGKRSRLTPVSPESSSTEEKSSSQPSSCCSDPSKPGGNVEGA
TQSLAEQMRKIALESEGFVSEEDEVDGWLIIDLPDSYAAPPSSGGSSGGSSGSETPGTSESATPESSGG

SSGGSFVSEEDEVDGWLIIDLPDSYAAPPSSGGSSGGSSGSETPGTSESATPESSGGSSGGSFVSEEDE
VDGWLIIDLPDSYAAPPSSKEVDPSTGELQSLQMPESEGPSSLDPSQEGPTGLKEAALYPHSGGSSGGS
SGSETPGTSESATPESSGGSSGGSMEVQLQESGGGLVQPGGSLRLSCTASGVTISALNAMAMGWYRQAP
GERRVMVAAVSERGNAMYRESVQGRFTVTRDFTNKMVSLQMDNLKPEDTAVYYCHVLEDRVDSFHDYWG
QGTQVTVSSSGGSSGGSSGSETPGTSESATPESSGGSSGGSMEVQLQESGGGLVQPGGSLRLSCTASGV
TISALNAMAMGWYRQAPGERRVMVAAVSERGNAMYRESVQGRFTVTRDFTNKMVSLQMDNLKPEDTAVY
YCHVLEDRVDSFHDYWGQGTQVTVSSSGGSSGGSSGSETPGTSESATPESSGGSSGGSMEVQLQESGGG
LVQPGGSLRLSCTASGVTISALNAMAMGWYRQAPGERRVMVAAVSERGNAMYRESVQGRFTVTRDFTNK
MVSLQMDNLKPEDTAVYYCHVLEDRVDSFHDYWGQGTQVTVSSRGRYPYDVPDYA*

ΔN-T1A1: LIR(TP53INP2)—Ab; Vector backbone: pcDNA3.1(+)

MAFVSEEDEVDGWLIIDLPDSYAAPPSSKEVDPSTGELQSLQMPESEGPSSLDPSQEGPTGLKEAALYP
HSGGSSGGSSGSETPGTSESATPESSGGSSGGSMEVQLQESGGGLVQPGGSLRLSCTASGVTISALNAM
AMGWYRQAPGERRVMVAAVSERGNAMYRESVQGRFTVTRDFTNKMVSLQMDNLKPEDTAVYYCHVLEDR
VDSFHDYWGQGTQVTVSSRGRYPYDVPDYA*

ΔN-T1A2: LIR(TP53INP2)—Ab—Ab; Vector backbone: pcDNA3.1(+)

MAFVSEEDEVDGWLIIDLPDSYAAPPSSKEVDPSTGELQSLQMPESEGPSSLDPSQEGPTGLKEAALYP
HSGGSSGGSSGSETPGTSESATPESSGGSSGGSMEVQLQESGGGLVQPGGSLRLSCTASGVTISALNAM
AMGWYRQAPGERRVMVAAVSERGNAMYRESVQGRFTVTRDFTNKMVSLQMDNLKPEDTAVYYCHVLEDR
VDSFHDYWGQGTQVTVSSSGGSSGGSSGSETPGTSESATPESSGGSSGGSMEVQLQESGGGLVQPGGSL
RLSCTASGVTISALNAMAMGWYRQAPGERRVMVAAVSERGNAMYRESVQGRFTVTRDFTNKMVSLQMDN
LKPEDTAVYYCHVLEDRVDSFHDYWGQGTQVTVSSRGRYPYDVPDYA*

ΔN-T2A2: LIR(TP53INP2)—LIR(TP53INP2)—Ab—Ab; Vector backbone: pcDNA3.1(+)

MAFVSEEDEVDGWLIIDLPDSYAAPPSSGGSSGGSSGSETPGTSESATPESSGGSSGGSFVSEEDEVDG
WLIIDLPDSYAAPPSSKEVDPSTGELQSLQMPESEGPSSLDPSQEGPTGLKEAALYPHSGGSSGGSSGS
ETPGTSESATPESSGGSSGGSMEVQLQESGGGLVQPGGSLRLSCTASGVTISALNAMAMGWYRQAPGER
RVMVAAVSERGNAMYRESVQGRFTVTRDFTNKMVSLQMDNLKPEDTAVYYCHVLEDRVDSFHDYWGQGT
QVTVSSSGGSSGGSSGSETPGTSESATPESSGGSSGGSMEVQLQESGGGLVQPGGSLRLSCTASGVTIS
ALNAMAMGWYRQAPGERRVMVAAVSERGNAMYRESVQGRFTVTRDFTNKMVSLQMDNLKPEDTAVYYCH
VLEDRVDSFHDYWGQGTQVTVSSRGRYPYDVPDYA*

ΔN-T1A3: LIR(TP53INP2)—Ab—Ab—Ab; Vector backbone: pcDNA3.1(+)

MAFVSEEDEVDGWLIIDLPDSYAAPPSSKEVDPSTGELQSLQMPESEGPSSLDPSQEGPTGLKEAALYP
HSGGSSGGSSGSETPGTSESATPESSGGSSGGSMEVQLQESGGGLVQPGGSLRLSCTASGVTISALNAM
AMGWYRQAPGERRVMVAAVSERGNAMYRESVQGRFTVTRDFTNKMVSLQMDNLKPEDTAVYYCHVLEDR
VDSFHDYWGQGTQVTVSSSGGSSGGSSGSETPGTSESATPESSGGSSGGSMEVQLQESGGGLVQPGGSL
RLSCTASGVTISALNAMAMGWYRQAPGERRVMVAAVSERGNAMYRESVQGRFTVTRDFTNKMVSLQMDN

LKPEDTAVYYCHVLEDRVDSFHDYWGQGTQVTVSSSGGSSGGSSGSETPGTSESATPESSGGSSGGSME
VQLQESGGGLVQPGGSLRLSCTASGVTISALNAMAMGWYRQAPGERRVMVAAVSERGNAMYRESVQGRF
TVTRDFTNKMVSLQMDNLKPEDTAVYYCHVLEDRVDSFHDYWGQGTQVTVSSRGRYPYDVPDYA*

ΔN-T2A3: LIR(TP53INP2)—LIR(TP53INP2)—Ab—Ab—Ab; Vector backbone: pcDNA3.1(+)

MAFVSEEDEVDGWLIIDLPDSYAAPPSSGGSSGGSSGSETPGTSESATPESSGGSSGGSFVSEEDEVDG
WLIIDLPDSYAAPPSSKEVDPSTGELQSLQMPESEGPSSLDPSQEGPTGLKEAALYPHSGGSSGGSSGS
ETPGTSESATPESSGGSSGGSMEVQLQESGGGLVQPGGSLRLSCTASGVTISALNAMAMGWYRQAPGER
RVMVAAVSERGNAMYRESVQGRFTVTRDFTNKMVSLQMDNLKPEDTAVYYCHVLEDRVDSFHDYWGQGT
QVTVSSSGGSSGGSSGSETPGTSESATPESSGGSSGGSMEVQLQESGGGLVQPGGSLRLSCTASGVTIS
ALNAMAMGWYRQAPGERRVMVAAVSERGNAMYRESVQGRFTVTRDFTNKMVSLQMDNLKPEDTAVYYCH
VLEDRVDSFHDYWGQGTQVTVSSSGGSSGGSSGSETPGTSESATPESSGGSSGGSMEVQLQESGGGLVQ
PGGSLRLSCTASGVTISALNAMAMGWYRQAPGERRVMVAAVSERGNAMYRESVQGRFTVTRDFTNKMVS
LQMDNLKPEDTAVYYCHVLEDRVDSFHDYWGQGTQVTVSSRGRYPYDVPDYA*

ΔN-T3A3: LIR(TP53INP2)—LIR(TP53INP2)—LIR(TP53INP2)—Ab—Ab—Ab; Vector backbone: pcDNA3.1(+) or Tet-On 3G

MAFVSEEDEVDGWLIIDLPDSYAAPPSSGGSSGGSSGSETPGTSESATPESSGGSSGGSFVSEEDEVDG
WLIIDLPDSYAAPPSSGGSSGGSSGSETPGTSESATPESSGGSSGGSFVSEEDEVDGWLIIDLPDSYAA
PPSSKEVDPSTGELQSLQMPESEGPSSLDPSQEGPTGLKEAALYPHSGGSSGGSSGSETPGTSESATPE
SSGGSSGGSMEVQLQESGGGLVQPGGSLRLSCTASGVTISALNAMAMGWYRQAPGERRVMVAAVSERGN
AMYRESVQGRFTVTRDFTNKMVSLQMDNLKPEDTAVYYCHVLEDRVDSFHDYWGQGTQVTVSSSGGSSG
GSSGSETPGTSESATPESSGGSSGGSMEVQLQESGGGLVQPGGSLRLSCTASGVTISALNAMAMGWYRQ
APGERRVMVAAVSERGNAMYRESVQGRFTVTRDFTNKMVSLQMDNLKPEDTAVYYCHVLEDRVDSFHDY
WGQGTQVTVSSSGGSSGGSSGSETPGTSESATPESSGGSSGGSMEVQLQESGGGLVQPGGSLRLSCTAS
GVTISALNAMAMGWYRQAPGERRVMVAAVSERGNAMYRESVQGRFTVTRDFTNKMVSLQMDNLKPEDTA
VYYCHVLEDRVDSFHDYWGQGTQVTVSSRGRYPYDVPDYA*

A1: Ab; Vector backbone: pcDNA3.1(+)

MEVQLQESGGGLVQPGGSLRLSCTASGVTISALNAMAMGWYRQAPGERRVMVAAVSERGNAMYRESVQG
RFTVTRDFTNKMVSLQMDNLKPEDTAVYYCHVLEDRVDSFHDYWGQGTQVTVSSRGRYPYDVPDYA*

A2: Ab—Ab; Vector backbone: pcDNA3.1(+)

MEVQLQESGGGLVQPGGSLRLSCTASGVTISALNAMAMGWYRQAPGERRVMVAAVSERGNAMYRESVQG
RFTVTRDFTNKMVSLQMDNLKPEDTAVYYCHVLEDRVDSFHDYWGQGTQVTVSSSGGSSGGSSGSETPG
TSESATPESSGGSSGGSMEVQLQESGGGLVQPGGSLRLSCTASGVTISALNAMAMGWYRQAPGERRVMV
AAVSERGNAMYRESVQGRFTVTRDFTNKMVSLQMDNLKPEDTAVYYCHVLEDRVDSFHDYWGQGTQVTV
SSRGRYPYDVPDYA*

A_{Ctrl}: Ab(RNase-A); Vector backbone: pcDNA3.1(+)

MFRQAPGKEREGVAAMDSGGGGTLYADSVKGRFTISRDKGKNTVYLQMDSLKPEDTATYYCAAGGYELR
DRTYGQWGQGTQVTVSSHAAAGAPVPYPDPLEPRRGRYPYDVPDYA*

ΔN-T1A_{Ctrl}: LIR(TP53INP2)—Ab(RNase-A); Vector backbone: pcDNA3.1(+)

MAFVSEEDEVDGWLIIDLPDSYAAPPSSKEVDPSTGELQSLQMPESEGPSSLDPSQEGPTGLKEAALYP
HSGGSSGGSSGSETPGTSESATPESSGGSSGGSMQVQLVESGGGLVQAGGSLRLSCAASGYAYTYIYMG
WFRQAPGKEREGVAAMDSGGGGTLYADSVKGRFTISRDKGKNTVYLQMDSLKPEDTATYYCAAGGYELR
DRTYGQWGQGTQVTVSSHAAAGAPVPYPDPLEPRRGRYPYDVPDYA*

BFP-T1A1: mTagBFP2—LIR(TP53INP2)—Ab; Vector backbone: pcDNA3.1(+)

MVSKGEELIKENMHMKLYMEGTVDNHHFKCTSEGEGKPYEGTQTMRIKVVEGGPLPFAFDILATSFLYG
SKTFINHTQGIPDFFKQSFPEGFTWERVTTYEDGGVLTATQDTSLQDGCLIYNVKIRGVNFTSNGPVMQ
KKTLGWEAFTETLYPADGGLEGRNDMALKLVGGSHLIANAKTTYRSKKPAKNLKMPGVYYVDYRLERIK
EANNETYVEQHEVAVARYCDLPSKLGHKLNSGLRSGGSGGFVSEEDEVDGWLIIDLPDSYAAPPSSKEV
DPSTGELQSLQMPESEGPSSLDPSQEGPTGLKEAALYPHSGGSSGGSSGSETPGTSESATPESSGGSSG
GSMEVQLQESGGGLVQPGGSLRLSCTASGVTISALNAMAMGWYRQAPGERRVMVAAVSERGNAMYRESV
QGRFTVTRDFTNKMVSLQMDNLKPEDTAVYYCHVLEDRVDSFHDYWGQGTQVTVSSRGRYPYDVPDYA*

BFP-T1A2: mTagBFP2—LIR(TP53INP2)—Ab—Ab; Vector backbone: pcDNA3.1(+)

MVSKGEELIKENMHMKLYMEGTVDNHHFKCTSEGEGKPYEGTQTMRIKVVEGGPLPFAFDILATSFLYG
SKTFINHTQGIPDFFKQSFPEGFTWERVTTYEDGGVLTATQDTSLQDGCLIYNVKIRGVNFTSNGPVMQ
KKTLGWEAFTETLYPADGGLEGRNDMALKLVGGSHLIANAKTTYRSKKPAKNLKMPGVYYVDYRLERIK
EANNETYVEQHEVAVARYCDLPSKLGHKLNSGLRSGGSGGFVSEEDEVDGWLIIDLPDSYAAPPSSKEV
DPSTGELQSLQMPESEGPSSLDPSQEGPTGLKEAALYPHSGGSSGGSSGSETPGTSESATPESSGGSSG
GSMEVQLQESGGGLVQPGGSLRLSCTASGVTISALNAMAMGWYRQAPGERRVMVAAVSERGNAMYRESV
QGRFTVTRDFTNKMVSLQMDNLKPEDTAVYYCHVLEDRVDSFHDYWGQGTQVTVSSSGGSSGGSSGSET
PGTSESATPESSGGSSGGSMEVQLQESGGGLVQPGGSLRLSCTASGVTISALNAMAMGWYRQAPGERRV
MVAAVSERGNAMYRESVQGRFTVTRDFTNKMVSLQMDNLKPEDTAVYYCHVLEDRVDSFHDYWGQGTQV
TVSSRGRYPYDVPDYA*

BFP-T2A2: mTagBFP2—LIR(TP53INP2)—LIR(TP53INP2)—Ab—Ab; Vector backbone: pcDNA3.1(+)

MVSKGEELIKENMHMKLYMEGTVDNHHFKCTSEGEGKPYEGTQTMRIKVVEGGPLPFAFDILATSFLYG
SKTFINHTQGIPDFFKQSFPEGFTWERVTTYEDGGVLTATQDTSLQDGCLIYNVKIRGVNFTSNGPVMQ
KKTLGWEAFTETLYPADGGLEGRNDMALKLVGGSHLIANAKTTYRSKKPAKNLKMPGVYYVDYRLERIK
EANNETYVEQHEVAVARYCDLPSKLGHKLNSGLRSGGSGGFVSEEDEVDGWLIIDLPDSYAAPPSSGGS

SGGSSGSETPGTSESATPESSGGSSGGSFVSEEDEVDGWLIIDLPDSYAAPPSSKEVDPSTGELQSLQM
PESEGPSSLDPSQEGPTGLKEAALYPHSGGSSGGSSGSETPGTSESATPESSGGSSGGSMEVQLQESGG
GLVQPGGSLRLSCTASGVTISALNAMAMGWYRQAPGERRVMVAAVSERGNAMYRESVQGRFTVTRDFTN
KMVSLQMDNLKPEDTAVYYCHVLEDRVDSFHDYWGQGTQVTVSSSGGSSGGSSGSETPGTSESATPESS
GGSSGGSMEVQLQESGGGLVQPGGSLRLSCTASGVTISALNAMAMGWYRQAPGERRVMVAAVSERGNAM
YRESVQGRFTVTRDFTNKMVSLQMDNLKPEDTAVYYCHVLEDRVDSFHDYWGQGTQVTVSSRGRYPYDV
PDYA*

BFP-T1A3: mTagBFP2—LIR(TP53INP2)—Ab—Ab—Ab; Vector backbone: pcDNA3.1(+)

MVSKGEELIKENMHMKLYMEGTVDNHHFKCTSEGEGKPYEGTQTMRIKVVEGGPLPFAFDILATSFLYG
SKTFINHTQGIPDFFKQSFPEGFTWERVTTYEDGGVLTATQDTSLQDGCLIYNVKIRGVNFTSNGPVMQ
KKTLGWEAFTETLYPADGGLEGRNDMALKLVGGSHLIANAKTTYRSKKPAKNLKMPGVYYVDYRLERIK
EANNETYVEQHEVAVARYCDLPSKLGHKLNSGLRSGGSGGFVSEEDEVDGWLIIDLPDSYAAPPSSKEV
DPSTGELQSLQMPESEGPSSLDPSQEGPTGLKEAALYPHSGGSSGGSSGSETPGTSESATPESSGGSSG
GSMEVQLQESGGGLVQPGGSLRLSCTASGVTISALNAMAMGWYRQAPGERRVMVAAVSERGNAMYRESV
QGRFTVTRDFTNKMVSLQMDNLKPEDTAVYYCHVLEDRVDSFHDYWGQGTQVTVSSSGGSSGGSSGSET
PGTSESATPESSGGSSGGSMEVQLQESGGGLVQPGGSLRLSCTASGVTISALNAMAMGWYRQAPGERRV
MVAAVSERGNAMYRESVQGRFTVTRDFTNKMVSLQMDNLKPEDTAVYYCHVLEDRVDSFHDYWGQGTQV
TVSSSGGSSGGSSGSETPGTSESATPESSGGSSGGSMEVQLQESGGGLVQPGGSLRLSCTASGVTISAL
NAMAMGWYRQAPGERRVMVAAVSERGNAMYRESVQGRFTVTRDFTNKMVSLQMDNLKPEDTAVYYCHVL
EDRVDSFHDYWGQGTQVTVSSRGRYPYDVPDYA*

BFP-T2A3: mTagBFP2—LIR(TP53INP2)—LIR(TP53INP2)—Ab—Ab—Ab; Vector backbone: pcDNA3.1(+)

MVSKGEELIKENMHMKLYMEGTVDNHHFKCTSEGEGKPYEGTQTMRIKVVEGGPLPFAFDILATSFLYG
SKTFINHTQGIPDFFKQSFPEGFTWERVTTYEDGGVLTATQDTSLQDGCLIYNVKIRGVNFTSNGPVMQ
KKTLGWEAFTETLYPADGGLEGRNDMALKLVGGSHLIANAKTTYRSKKPAKNLKMPGVYYVDYRLERIK
EANNETYVEQHEVAVARYCDLPSKLGHKLNSGLRSGGSGGFVSEEDEVDGWLIIDLPDSYAAPPSSGGS
SGGSSGSETPGTSESATPESSGGSSGGSFVSEEDEVDGWLIIDLPDSYAAPPSSKEVDPSTGELQSLQM
PESEGPSSLDPSQEGPTGLKEAALYPHSGGSSGGSSGSETPGTSESATPESSGGSSGGSMEVQLQESGG
GLVQPGGSLRLSCTASGVTISALNAMAMGWYRQAPGERRVMVAAVSERGNAMYRESVQGRFTVTRDFTN
KMVSLQMDNLKPEDTAVYYCHVLEDRVDSFHDYWGQGTQVTVSSSGGSSGGSSGSETPGTSESATPESS
GGSSGGSMEVQLQESGGGLVQPGGSLRLSCTASGVTISALNAMAMGWYRQAPGERRVMVAAVSERGNAM
YRESVQGRFTVTRDFTNKMVSLQMDNLKPEDTAVYYCHVLEDRVDSFHDYWGQGTQVTVSSSGGSSGGS
SGSETPGTSESATPESSGGSSGGSMEVQLQESGGGLVQPGGSLRLSCTASGVTISALNAMAMGWYRQAP
GERRVMVAAVSERGNAMYRESVQGRFTVTRDFTNKMVSLQMDNLKPEDTAVYYCHVLEDRVDSFHDYWG
QGTQVTVSSRGRYPYDVPDYA*

BFP-T3A3: mTagBFP2—LIR(TP53INP2)—LIR(TP53INP2)—LIR(TP53INP2)—Ab—Ab—Ab; Vector backbone:

pcDNA3.1(+)

MVSKGEELIKENMHMKLYMEGTVDNHHFKCTSEGEGKPYEGTQTMRIKVVEGGPLPFAFDILATSFLYG
SKTFINHTQGIPDFFKQSFPEGFTWERVTTYEDGGVLTATQDTSLQDGCLIYNVKIRGVNFTSNGPVMQ
KKTLGWEAFTETLYPADGGLEGRNDMALKLVGGSHLIANAKTTYRSKKPAKNLKMPGVYYVDYRLERIK
EANNETYVEQHEVAVARYCDLPSKLGHKLNSGLRSGGSGGFVSEEDEVDGWLIIDLPDSYAAPPSSGGS
SGGSSGSETPGTSESATPESSGGSSGGSFVSEEDEVDGWLIIDLPDSYAAPPSSGGSSGGSSGSETPGT
SESATPESSGGSSGGSFVSEEDEVDGWLIIDLPDSYAAPPSSKEVDPSTGELQSLQMPESEGPSSLDPS
QEGPTGLKEAALYPHSGGSSGGSSGSETPGTSESATPESSGGSSGGSMEVQLQESGGGLVQPGGSLRLS
CTASGVTISALNAMAMGWYRQAPGERRVMVAAVSERGNAMYRESVQGRFTVTRDFTNKMVSLQMDNLKP
EDTAVYYCHVLEDRVDSFHDYWGQGTQVTVSSSGGSSGGSSGSETPGTSESATPESSGGSSGGSMEVQL
QESGGGLVQPGGSLRLSCTASGVTISALNAMAMGWYRQAPGERRVMVAAVSERGNAMYRESVQGRFTVT
RDFTNKMVSLQMDNLKPEDTAVYYCHVLEDRVDSFHDYWGQGTQVTVSSSGGSSGGSSGSETPGTSESA
TPESSGGSSGGSMEVQLQESGGGLVQPGGSLRLSCTASGVTISALNAMAMGWYRQAPGERRVMVAAVSE
RGNAMYRESVQGRFTVTRDFTNKMVSLQMDNLKPEDTAVYYCHVLEDRVDSFHDYWGQGTQVTVSSRGR
YPYDVPDYA*

BFP-A1: mTagBFP2—Ab; Vector backbone: pcDNA3.1(+)

MVSKGEELIKENMHMKLYMEGTVDNHHFKCTSEGEGKPYEGTQTMRIKVVEGGPLPFAFDILATSFLYG
SKTFINHTQGIPDFFKQSFPEGFTWERVTTYEDGGVLTATQDTSLQDGCLIYNVKIRGVNFTSNGPVMQ
KKTLGWEAFTETLYPADGGLEGRNDMALKLVGGSHLIANAKTTYRSKKPAKNLKMPGVYYVDYRLERIK
EANNETYVEQHEVAVARYCDLPSKLGHKLNSGLRSGGSGGMEVQLQESGGGLVQPGGSLRLSCTASGVT
ISALNAMAMGWYRQAPGERRVMVAAVSERGNAMYRESVQGRFTVTRDFTNKMVSLQMDNLKPEDTAVYY
CHVLEDRVDSFHDYWGQGTQVTVSSRGRYPYDVPDYA*

BFP-A$_{Ctrl}$: mTagBFP2—Ab(RNase-A); Vector backbone: pcDNA3.1(+)

MVSKGEELIKENMHMKLYMEGTVDNHHFKCTSEGEGKPYEGTQTMRIKVVEGGPLPFAFDILATSFLYG
SKTFINHTQGIPDFFKQSFPEGFTWERVTTYEDGGVLTATQDTSLQDGCLIYNVKIRGVNFTSNGPVMQ
KKTLGWEAFTETLYPADGGLEGRNDMALKLVGGSHLIANAKTTYRSKKPAKNLKMPGVYYVDYRLERIK
EANNETYVEQHEVAVARYCDLPSKLGHKLNSGLRSGGSGGMQVQLVESGGGLVQAGGSLRLSCAASGYA
YTYIYMGWFRQAPGKEREGVAAMDSGGGGTLYADSVKGRFTISRDKGKNTVYLQMDSLKPEDTATYYCA
AGGYELRDRTYGQWGQGTQVTVSSHAAAGAPVPYPDPLEPRRGRYPYDVPDYA*

BFP-T1A$_{Ctrl}$: mTagBFP2—LIR(TP53INP2)—Ab(RNase-A); Vector backbone: pcDNA3.1(+)

MVSKGEELIKENMHMKLYMEGTVDNHHFKCTSEGEGKPYEGTQTMRIKVVEGGPLPFAFDILATSFLYG
SKTFINHTQGIPDFFKQSFPEGFTWERVTTYEDGGVLTATQDTSLQDGCLIYNVKIRGVNFTSNGPVMQ
KKTLGWEAFTETLYPADGGLEGRNDMALKLVGGSHLIANAKTTYRSKKPAKNLKMPGVYYVDYRLERIK
EANNETYVEQHEVAVARYCDLPSKLGHKLNSGLRSGGSGGFVSEEDEVDGWLIIDLPDSYAAPPSSKEV
DPSTGELQSLQMPESEGPSSLDPSQEGPTGLKEAALYPHSGGSSGGSSGSETPGTSESATPESSGGSSG
GSMQVQLVESGGGLVQAGGSLRLSCAASGYAYTYIYMGWFRQAPGKEREGVAAMDSGGGGTLYADSVKG
RFTISRDKGKNTVYLQMDSLKPEDTATYYCAAGGYELRDRTYGQWGQGTQVTVSSHAAAGAPVPYPDPL
EPRRGRYPYDVPDYA*

mPlum-T1A1: mPlum—LIR(TP53INP2)—Ab; Vector backbone: pcDNA3.1(+)

MVSKGEEVIKEFMRFKEHMEGSVNGHEFEIEGEGEGRPYEGTQTARLKVTKGGPLPFAWDILSPQIMYG
SKAYVKHPADIPDYLKLSFPEGFKWERVMNFEDGGVVTVTQDSSLQDGEFIYKVKVRGTNFPSDGPVMQ
KKTMGWEASSERMYPEDGALKGEMKMRLRLKDGGHYDAEVKTTYMAKKPVQLPGAYKTDIKLDITSHNE
DYTIVEQYERAEGRHSTGASGLRSGGSGGFVSEEDEVDGWLIIDLPDSYAAPPSSKEVDPSTGELQSLQ
MPESEGPSSLDPSQEGPTGLKEAALYPHSGGSSGGSSGSETPGTSESATPESSGGSSGGSMEVQLQESG
GGLVQPGGSLRLSCTASGVTISALNAMAMGWYRQAPGERRVMVAAVSERGNAMYRESVQGRFTVTRDFT
NKMVSLQMDNLKPEDTAVYYCHVLEDRVDSFHDYWGQGTQVTVSSRGRYPYDVPDYA*

mPlum-T1A2: mPlum—LIR(TP53INP2)—Ab—Ab; Vector backbone: pcDNA3.1(+)

MVSKGEEVIKEFMRFKEHMEGSVNGHEFEIEGEGEGRPYEGTQTARLKVTKGGPLPFAWDILSPQIMYG
SKAYVKHPADIPDYLKLSFPEGFKWERVMNFEDGGVVTVTQDSSLQDGEFIYKVKVRGTNFPSDGPVMQ
KKTMGWEASSERMYPEDGALKGEMKMRLRLKDGGHYDAEVKTTYMAKKPVQLPGAYKTDIKLDITSHNE
DYTIVEQYERAEGRHSTGASGLRSGGSGGFVSEEDEVDGWLIIDLPDSYAAPPSSKEVDPSTGELQSLQ
MPESEGPSSLDPSQEGPTGLKEAALYPHSGGSSGGSSGSETPGTSESATPESSGGSSGGSMEVQLQESG
GGLVQPGGSLRLSCTASGVTISALNAMAMGWYRQAPGERRVMVAAVSERGNAMYRESVQGRFTVTRDFT
NKMVSLQMDNLKPEDTAVYYCHVLEDRVDSFHDYWGQGTQVTVSSSGGSSGGSSGSETPGTSESATPES
SGGSSGGSMEVQLQESGGGLVQPGGSLRLSCTASGVTISALNAMAMGWYRQAPGERRVMVAAVSERGNA
MYRESVQGRFTVTRDFTNKMVSLQMDNLKPEDTAVYYCHVLEDRVDSFHDYWGQGTQVTVSSRGRYPYD
VPDYA*

mPlum-T2A2: mPlum—LIR(TP53INP2)—LIR(TP53INP2)—Ab—Ab; Vector backbone: pcDNA3.1(+)

MVSKGEEVIKEFMRFKEHMEGSVNGHEFEIEGEGEGRPYEGTQTARLKVTKGGPLPFAWDILSPQIMYG
SKAYVKHPADIPDYLKLSFPEGFKWERVMNFEDGGVVTVTQDSSLQDGEFIYKVKVRGTNFPSDGPVMQ
KKTMGWEASSERMYPEDGALKGEMKMRLRLKDGGHYDAEVKTTYMAKKPVQLPGAYKTDIKLDITSHNE
DYTIVEQYERAEGRHSTGASGLRSGGSGGFVSEEDEVDGWLIIDLPDSYAAPPSSGGSSGGSSGSETPG
TSESATPESSGGSSGGSFVSEEDEVDGWLIIDLPDSYAAPPSSKEVDPSTGELQSLQMPESEGPSSLDP
SQEGPTGLKEAALYPHSGGSSGGSSGSETPGTSESATPESSGGSSGGSMEVQLQESGGGLVQPGGSLRL
SCTASGVTISALNAMAMGWYRQAPGERRVMVAAVSERGNAMYRESVQGRFTVTRDFTNKMVSLQMDNLK
PEDTAVYYCHVLEDRVDSFHDYWGQGTQVTVSSSGGSSGGSSGSETPGTSESATPESSGGSSGGSMEVQ
LQESGGGLVQPGGSLRLSCTASGVTISALNAMAMGWYRQAPGERRVMVAAVSERGNAMYRESVQGRFTV
TRDFTNKMVSLQMDNLKPEDTAVYYCHVLEDRVDSFHDYWGQGTQVTVSSRGRYPYDVPDYA*

mPlum-T1A3: mPlum—LIR(TP53INP2)—Ab—Ab—Ab; Vector backbone: pcDNA3.1(+)

MVSKGEEVIKEFMRFKEHMEGSVNGHEFEIEGEGEGRPYEGTQTARLKVTKGGPLPFAWDILSPQIMYG
SKAYVKHPADIPDYLKLSFPEGFKWERVMNFEDGGVVTVTQDSSLQDGEFIYKVKVRGTNFPSDGPVMQ
KKTMGWEASSERMYPEDGALKGEMKMRLRLKDGGHYDAEVKTTYMAKKPVQLPGAYKTDIKLDITSHNE
DYTIVEQYERAEGRHSTGASGLRSGGSGGFVSEEDEVDGWLIIDLPDSYAAPPSSKEVDPSTGELQSLQ
MPESEGPSSLDPSQEGPTGLKEAALYPHSGGSSGGSSGSETPGTSESATPESSGGSSGGSMEVQLQESG
GGLVQPGGSLRLSCTASGVTISALNAMAMGWYRQAPGERRVMVAAVSERGNAMYRESVQGRFTVTRDFT

NKMVSLQMDNLKPEDTAVYYCHVLEDRVDSFHDYWGQGTQVTVSSSGGSSGGSSGSETPGTSESATPES
SGGSSGGSMEVQLQESGGGLVQPGGSLRLSCTASGVTISALNAMAMGWYRQAPGERRVMVAAVSERGNA
MYRESVQGRFTVTRDFTNKMVSLQMDNLKPEDTAVYYCHVLEDRVDSFHDYWGQGTQVTVSSSGGSSGG
SSGSETPGTSESATPESSGGSSGGSMEVQLQESGGGLVQPGGSLRLSCTASGVTISALNAMAMGWYRQA
PGERRVMVAAVSERGNAMYRESVQGRFTVTRDFTNKMVSLQMDNLKPEDTAVYYCHVLEDRVDSFHDYW
GQGTQVTVSSRGRYPYDVPDYA*

mPlum-T2A3: mPlum—LIR(TP53INP2)—LIR(TP53INP2)—Ab—Ab—Ab; Vector backbone: pcDNA3.1(+)

MVSKGEEVIKEFMRFKEHMEGSVNGHEFEIEGEGEGRPYEGTQTARLKVTKGGPLPFAWDILSPQIMYG
SKAYVKHPADIPDYLKLSFPEGFKWERVMNFEDGGVVTVTQDSSLQDGEFIYKVKVRGTNFPSDGPVMQ
KKTMGWEASSERMYPEDGALKGEMKMRLRLKDGGHYDAEVKTTYMAKKPVQLPGAYKTDIKLDITSHNE
DYTIVEQYERAEGRHSTGASGLRSGGSGGFVSEEDEVDGWLIIDLPDSYAAPPSSGGSSGGSSGSETPG
TSESATPESSGGSSGGSFVSEEDEVDGWLIIDLPDSYAAPPSSKEVDPSTGELQSLQMPESEGPSSLDP
SQEGPTGLKEAALYPHSGGSSGGSSGSETPGTSESATPESSGGSSGGSMEVQLQESGGGLVQPGGSLRL
SCTASGVTISALNAMAMGWYRQAPGERRVMVAAVSERGNAMYRESVQGRFTVTRDFTNKMVSLQMDNLK
PEDTAVYYCHVLEDRVDSFHDYWGQGTQVTVSSSGGSSGGSSGSETPGTSESATPESSGGSSGGSMEVQ
LQESGGGLVQPGGSLRLSCTASGVTISALNAMAMGWYRQAPGERRVMVAAVSERGNAMYRESVQGRFTV
TRDFTNKMVSLQMDNLKPEDTAVYYCHVLEDRVDSFHDYWGQGTQVTVSSSGGSSGGSSGSETPGTSES
ATPESSGGSSGGSMEVQLQESGGGLVQPGGSLRLSCTASGVTISALNAMAMGWYRQAPGERRVMVAAVS
ERGNAMYRESVQGRFTVTRDFTNKMVSLQMDNLKPEDTAVYYCHVLEDRVDSFHDYWGQGTQVTVSSRG
RYPYDVPDYA*

mPlum-T3A3: mPlum—LIR(TP53INP2)—LIR(TP53INP2)—LIR(TP53INP2)—Ab—Ab—Ab; Vector backbone:

pcDNA3.1(+)

MVSKGEEVIKEFMRFKEHMEGSVNGHEFEIEGEGEGRPYEGTQTARLKVTKGGPLPFAWDILSPQIMYG
SKAYVKHPADIPDYLKLSFPEGFKWERVMNFEDGGVVTVTQDSSLQDGEFIYKVKVRGTNFPSDGPVMQ
KKTMGWEASSERMYPEDGALKGEMKMRLRLKDGGHYDAEVKTTYMAKKPVQLPGAYKTDIKLDITSHNE
DYTIVEQYERAEGRHSTGASGLRSGGSGGFVSEEDEVDGWLIIDLPDSYAAPPSSGGSSGGSSGSETPG
TSESATPESSGGSSGGSFVSEEDEVDGWLIIDLPDSYAAPPSSGGSSGGSSGSETPGTSESATPESSGG
SSGGSFVSEEDEVDGWLIIDLPDSYAAPPSSKEVDPSTGELQSLQMPESEGPSSLDPSQEGPTGLKEAA
LYPHSGGSSGGSSGSETPGTSESATPESSGGSSGGSMEVQLQESGGGLVQPGGSLRLSCTASGVTISAL
NAMAMGWYRQAPGERRVMVAAVSERGNAMYRESVQGRFTVTRDFTNKMVSLQMDNLKPEDTAVYYCHVL
EDRVDSFHDYWGQGTQVTVSSSGGSSGGSSGSETPGTSESATPESSGGSSGGSMEVQLQESGGGLVQPG
GSLRLSCTASGVTISALNAMAMGWYRQAPGERRVMVAAVSERGNAMYRESVQGRFTVTRDFTNKMVSLQ
MDNLKPEDTAVYYCHVLEDRVDSFHDYWGQGTQVTVSSSGGSSGGSSGSETPGTSESATPESSGGSSGG
SMEVQLQESGGGLVQPGGSLRLSCTASGVTISALNAMAMGWYRQAPGERRVMVAAVSERGNAMYRESVQ
GRFTVTRDFTNKMVSLQMDNLKPEDTAVYYCHVLEDRVDSFHDYWGQGTQVTVSSRGRYPYDVPDYA*

mPlum-A1: mPlum—Ab; Vector backbone: pcDNA3.1(+)

MVSKGEEVIKEFMRFKEHMEGSVNGHEFEIEGEGEGRPYEGTQTARLKVTKGGPLPFAWDILSPQIMYG
SKAYVKHPADIPDYLKLSFPEGFKWERVMNFEDGGVVTVTQDSSLQDGEFIYKVKVRGTNFPSDGPVMQ
KKTMGWEASSERMYPEDGALKGEMKMRLRLKDGGHYDAEVKTTYMAKKPVQLPGAYKTDIKLDITSHNE
DYTIVEQYERAEGRHSTGASGLRSGGSGGMEVQLQESGGGLVQPGGSLRLSCTASGVTISALNAMAMGW
YRQAPGERRVMVAAVSERGNAMYRESVQGRFTVTRDFTNKMVSLQMDNLKPEDTAVYYCHVLEDRVDSF
HDYWGQGTQVTVSSRGRYPYDVPDYA*

mPlum-A$_{Ctrl}$: mPlum—Ab(RNase-A); Vector backbone: pcDNA3.1(+)

MVSKGEEVIKEFMRFKEHMEGSVNGHEFEIEGEGEGRPYEGTQTARLKVTKGGPLPFAWDILSPQIMYG
SKAYVKHPADIPDYLKLSFPEGFKWERVMNFEDGGVVTVTQDSSLQDGEFIYKVKVRGTNFPSDGPVMQ
KKTMGWEASSERMYPEDGALKGEMKMRLRLKDGGHYDAEVKTTYMAKKPVQLPGAYKTDIKLDITSHNE
DYTIVEQYERAEGRHSTGASGLRSGGSGGMQVQLVESGGGLVQAGGSLRLSCAASGYAYTYIYMGWFRQ
APGKEREGVAAMDSGGGGTLYADSVKGRFTISRDKGKNTVYLQMDSLKPEDTATYYCAAGGYELRDRTY
GQWGQGTQVTVSSHAAAGAPVPYPDPLEPRRGRYPYDVPDYA*

mPlum-T1A$_{Ctrl}$: mPlum—LIR(TP53INP2)—Ab(RNase-A); Vector backbone: pcDNA3.1(+)

MVSKGEEVIKEFMRFKEHMEGSVNGHEFEIEGEGEGRPYEGTQTARLKVTKGGPLPFAWDILSPQIMYG
SKAYVKHPADIPDYLKLSFPEGFKWERVMNFEDGGVVTVTQDSSLQDGEFIYKVKVRGTNFPSDGPVMQ
KKTMGWEASSERMYPEDGALKGEMKMRLRLKDGGHYDAEVKTTYMAKKPVQLPGAYKTDIKLDITSHNE
DYTIVEQYERAEGRHSTGASGLRSGGSGGFVSEEDEVDGWLIIDLPDSYAAPPSSKEVDPSTGELQSLQ
MPESEGPSSLDPSQEGPTGLKEAALYPHSGGSSGGSSGSETPGTSESATPESSGGSSGGSMQVQLVESG
GGLVQAGGSLRLSCAASGYAYTYIYMGWFRQAPGKEREGVAAMDSGGGGTLYADSVKGRFTISRDKGKN
TVYLQMDSLKPEDTATYYCAAGGYELRDRTYGQWGQGTQVTVSSHAAAGAPVPYPDPLEPRRGRYPYDV
PDYA*

3.2. Targets

HTT-103Q: HiBiT—HTT-103Q—ALFA; Vector backbone: pHSV

MVSGWRLFKKISGSSGMATLEKLMKAFESLKSFQQQQQQQQQQQQQQQQQQQQQQQQQQQQQQQQQQQQ
QQQQQQQQQQQQQQQQQQQQQQQQQQQQQQQQQQQQQQQQQQQQQQQQQQQQQQQQQQQQQQQPPPPPPPP
PPPQLPQPPPQAQPLLPQPQPPPPPPPPPPPGPAVAEEPLHRPSGGSRLEEELRRRLTE*

TDP-43-Q331K: HiBiT—TDP-43-Q331K—ALFA; Vector backbone: pHSV

MVSGWRLFKKISGSSGMSEYIRVTEDENDEPIEIPSEDDGTVLLSTVTAQFPGACGLRYRNPVSQCMRG
VRLVEGILHAPDAGWGNLVYVVNYPKDNKRKMDETDASSAVKVKRAVQKTSDLIVLGLPWKTTEQDLKE
YFSTFGEVLMVQVKKDLKTGHSKGFGFVRFTEYETQVKVMSQRHMIDGRWCDCKLPNSKQSQDEPLRSR
KVFVGRCTEDMTEDELREFFSQYGDVMDVFIPKPFRAFAFVTFADDQIAQSLCGEDLIIKGISVHISNA
EPKHNSNRQLERSGRFGGNPGGFGNQGGFGNSRGGGAGLGNNQGSNMGGGMNFGAFSINPAMMAAAQAA

LKSSWGMMGMLASQQNQSGPSGNNQNQGNMQREPNQAFGSGNNSYSGSNSGAAIGWGSASNAGSGSGFN
GGFGSSMDSKSSGWGMSGGSRLEEELRRRLTE*

FUS-P525L: HiBiT—FUS-P525L—ALFA; Vector backbone: pHSV

MVSGWRLFKKISGSSGMASNDYTQQATQSYGAYPTQPGQGYSQQSSQPYGQQSYSGYSQSTDTSGYGQS
SYSSYGQSQNTGYGTQSTPQGYGSTGGYGSSQSSQSSYGQQSSYPGYGQQPAPSSTSGSYGSSSQSSSY
GQPQSGSYSQQPSYGGQQQSYGQQQSYNPPQGYGQQNQYNSSSGGGGGGGGGGNYGQDQSSMSSGGGSG
GGYGNQDQSGGGGSGGYGQQDRGGRGRGGSGGGGGGGGGGYNRSSGGYEPRGRGGGRGGRGGMGGSDRG
GFNKFGGPRDQGSRHDSEQDNSDNNTIFVQGLGENVTIESVADYFKQIGIIKTNKKTGQPMINLYTDRE
TGKLKGEATVSFDDPPSAKAAIDWFDGKEFSGNPIKVSFATRRADFNRGGGNGRGGRGRGGPMGRGGYG
GGGSGGGGRGGFPSGGGGGGGQQRAGDWKCPNPTCENMNFSWRNECNQCKAPKPDGPGGGPGGSHMGGN
YGDDRRGGRGGYDRGGYRGRGGDRGGFRGGRGGGDRGGFGPGKMDSRGEHRQDRRERLYSGGSRLEEEL
RRRLTE*

α-Synuclein: HiBiT—α-Synuclein—ALFA; Vector backbone: pHSV

MVSGWRLFKKISGSSGMDVFMKGLSKAKEGVVAAAEKTKQGVAEAAGKTKEGVLYVGSKTKEGVVHGVA
TVAEKTKEQVTNVGGAVVTGVTAVAQKTVEGAGSIAAATGFVKKDQLGKNEEGAPQEGILEDMPVDPDN
EAYEMPSEEGYQDYEPEASGGSRLEEELRRRLTE*

Tau-WT: HiBiT—Tau-WT—ALFA; Vector backbone: pHSV

MVSGWRLFKKISGSSGMAEPRQEFEVMEDHAGTYGLGDRKDQGGYTMHQDQEGDTDAGLKESPLQTPTE
DGSEEPGSETSDAKSTPTAEDVTAPLVDEGAPGKQAAAQPHTEIPEGTTAEEAGIGDTPSLEDEAAGHV
TQARMVSKSKDGTGSDDKKAKGADGKTKIATPRGAAPPGQKGQANATRIPAKTPPAPKTPPSSGEPPKS
GDRSGYSSPGSPGTPGSRSRTPSLPTPPTREPKKVAVVRTPPKSPSSAKSRLQTAPVPMPDLKNVKSKI
GSTENLKHQPGGGKVQIINKKLDLSNVQSKCGSKDNIKHVPGGGSVQIVYKPVDLSKVTSKCGSLGNIH
HKPGGGQVEVKSEKLDFKDRVQSKIGSLDNITHVPGGGNKKIETHKLTFRENAKAKTDHGAEIVYKSPV
VSGDTSPRHLSNVSSTGSIDMVDSPQLATLADEVSASLAKQGLSGGSRLEEELRRRLTE*

Tau-P301L: HiBiT—Tau-P301L—ALFA; Vector backbone: pHSV

MVSGWRLFKKISGSSGMAEPRQEFEVMEDHAGTYGLGDRKDQGGYTMHQDQEGDTDAGLKESPLQTPTE
DGSEEPGSETSDAKSTPTAEDVTAPLVDEGAPGKQAAAQPHTEIPEGTTAEEAGIGDTPSLEDEAAGHV
TQARMVSKSKDGTGSDDKKAKGADGKTKIATPRGAAPPGQKGQANATRIPAKTPPAPKTPPSSGEPPKS
GDRSGYSSPGSPGTPGSRSRTPSLPTPPTREPKKVAVVRTPPKSPSSAKSRLQTAPVPMPDLKNVKSKI
GSTENLKHQPGGGKVQIINKKLDLSNVQSKCGSKDNIKHVLGGGSVQIVYKPVDLSKVTSKCGSLGNIH
HKPGGGQVEVKSEKLDFKDRVQSKIGSLDNITHVPGGGNKKIETHKLTFRENAKAKTDHGAEIVYKSPV
VSGDTSPRHLSNVSSTGSIDMVDSPQLATLADEVSASLAKQGLSGGSRLEEELRRRLTE*

HTT-103Q: HTT-103Q—ALFA; Vector backbone: pcDNA3.1(+) (For co-IP)

MATLEKLMKAFESLKSFQQQQQQQQQQQQQQQQQQQQQQQQQQQQQQQQQQQQQQQQQQQQQQQQQQQ
QQQQQQQQQQQQQQQQQQQQQQQQQQQQQQQQQQQQQQQQQQQQQPPPPPPPPPPPQLPQPPPQAQPLL
PQPQPPPPPPPPPPGPAVAEEPLHRPSGGSRLEEELRRRLTE*

Survivin: HiBiT—Survivin—ALFA; Vector backbone: pHSV

MVSGWRLFKKISGSSGMGAPTLPPAWQPFLKDHRISTFKNWPFLEGCACTPERMAEAGFIHCPTENEPD
LAQCFFCFKELEGWEPDDDPIGPGTVAYACNTSTLGGRGGRITREEHKKHSSGCAFLSVKKQFEELTLG
EFLKLDRERAKNKIAKETNNKKKEFEETAEKVRRAIEQLAAMDSGGSRLEEELRRRLTE*

3.3. Membrane anchors

MitoAnchor: OTS—SNAP-tag—ALFA; Vector backbone: pcDNA3.1(+) or pHSV

MVGRNSAIAAGVCGALFIGYCIYFDRKRRSDPNFKSRMDKDCEMKRTTLDSPLGKLELSGCEQGLHEIK
LLGKGTSAADAVEVPAPAAVLGGPEPLMQATAWLNAYFHQPEAIEEFPVPALHHPVFQQESFTRQVLWK
LLKVVKFGEVISYQQLAALAGNPAATAAVKTALSGNPVPILIPCHRVVSSSGAVGGYEGGLAVKEWLLA
HEGHRLGKPGLGSGGSRLEEELRRRLTE*

Membrane-anchor (Peroxisome): OTS—SNAP-tag—ALFA; Vector backbone: pcDNA3.1(+)

MLRSVWNFLKRHKKKCIFLGTVLGGVYILGKYGQKKIREIQESGRATMDKDCEMKRTTLDSPLGKLELS
GCEQGLHEIKLLGKGTSAADAVEVPAPAAVLGGPEPLMQATAWLNAYFHQPEAIEEFPVPALHHPVFQQ
ESFTRQVLWKLLKVVKFGEVISYQQLAALAGNPAATAAVKTALSGNPVPILIPCHRVVSSSGAVGGYEG
GLAVKEWLLAHEGHRLGKPGLGSGGSRLEEELRRRLTE*

Membrane-anchor (ER): OTS—SNAP-tag—ALFA; Vector backbone: pcDNA3.1(+)

MDPVVVLGLCLSCLLLLSLWKQSYGGGKLGTELGSMDKDCEMKRTTLDSPLGKLELSGCEQGLHEIKLL
GKGTSAADAVEVPAPAAVLGGPEPLMQATAWLNAYFHQPEAIEEFPVPALHHPVFQQESFTRQVLWKLL
KVVKFGEVISYQQLAALAGNPAATAAVKTALSGNPVPILIPCHRVVSSSGAVGGYEGGLAVKEWLLAHE
GHRLGKPGLGSGGSRLEEELRRRLTE*

Membrane-anchor (Golgi apparatus): OTS—SNAP-tag—ALFA; Vector backbone: pcDNA3.1(+)

MGNLKSVAQEPGPPCGLGLGLGLGLCGKQGPATPAGGGGGGGGGGMDKDCEMKRTTLDSPLGKLELSGC
EQGLHEIKLLGKGTSAADAVEVPAPAAVLGGPEPLMQATAWLNAYFHQPEAIEEFPVPALHHPVFQQES
FTRQVLWKLLKVVKFGEVISYQQLAALAGNPAATAAVKTALSGNPVPILIPCHRVVSSSGAVGGYEGGL
AVKEWLLAHEGHRLGKPGLGSGGSRLEEELRRRLTE*

MitoAnchor-deGFP: OTS—SNAP-tag—deGFP—ALFA; Vector backbone: pcDNA3.1(+) or pHSV

MVGRNSAIAAGVCGALFIGYCIYFDRKRRSDPNFKSRMDKDCEMKRTTLDSPLGKLELSGCEQGLHEIK
LLGKGTSAADAVEVPAPAAVLGGPEPLMQATAWLNAYFHQPEAIEEFPVPALHHPVFQQESFTRQVLWK
LLKVVKFGEVISYQQLAALAGNPAATAAVKTALSGNPVPILIPCHRVVSSSGAVGGYEGGLAVKEWLLA
HEGHRLGKPGLGSGSGSGPVATMVSKGEELFTGVVPILVELDGDVNGHKFSVSGEGEGDATYGKLTLKF
ICTTGKLPVPWPTLVTTLTYGVQCFSRYPDHMKQHDFFKSAMPEGYVQERTIFFKDDGNYKTRAEVKFE
GDTLVNRIELKGIDFKEDGNILGHKLEYNYNSHNVYIMADKQKNGIKVNFKIRHNIEDGSVQLADHYQQ
NTPIGDGPVLLPDNHYLSTQSALSKDPNEKRDHMVLLEFVTAAGITLGMDELYKKLSHGFPPEVEEQDD
GTLPMSCAQESGMDRHPAACASARINVSGGSRLEEELRRRLTE*

Addgene Plasmid References

P62, Cat# 28027; Ab(RNase-A), Cat#108241; HTT-103Q, Cat# 1186;

TDP-43-Q331K, Cat# 27465; FUS-P525L, Cat# 29628; α-Synuclein, Cat# 51437;

Tau-WT, Cat# 140424; Tau-P301L, Cat# 140425; mTagBFP2, Cat# 54572;

mPlum, Cat# 54839; deGFP, Cat# 26821.

4. References

SR1. Li, X.; Zhao, X.; Fang, Y.; Jiang, X.; Duong, T.; Fan, C.; Huang, C.-C.; Kain, S. R. Generation of Destabilized Green Fluorescent Protein as a Transcription Reporter. *J. Biol. Chem.* **1998**, *273*, 34970-34975.

SR2. Korolchuk, V. I.; Mansilla, A.; Menzies, F. M.; Rubinsztein, D. C. Autophagy inhibition compromises degradation of ubiquitin-proteasome pathway substrates. *Mol. Cell* **2009**, *33*, 517-527.

SR3. Vong, Q. P.; Cao, K.; Li, H. Y.; Iglesias, P. A.; Zheng, Y. Chromosome alignment and segregation regulated by ubiquitination of survivin. *Science* **2005**, *310*, 1499-1504.

SR4. Gotzke, H.; Kilisch, M.; Martinez-Carranza, M.; Sograte-Idrissi, S.; Rajavel, A.; Schlichthaerle, T.; Engels, N.; Jungmann, R.; Stenmark, P.; Opazo, F.; Frey, S. The ALFA-tag is a highly versatile tool for nanobody-based bioscience applications. *Nat. Commun.* **2019**, *10*, 4403.

SR5. Gaudelli, N. M.; Komor, A. C.; Rees, H. A.; Packer, M. S.; Badran, A. H.; Bryson, D. I.; Liu, D. R. Programmable base editing of A.T to G.C in genomic DNA without DNA cleavage. *Nature* **2017**, *551*, 464–471.

SR6. Jiang, Z. W.; Kuo, Y. H.; Zhong, M. Q.; Zhang, J. C.; Zhou, X. X.; Xing, L. J.; Wells, J. A.; Wang, Y. Z.; Arkin, M. R. Adaptor-Specific Antibody Fragment Inhibitors for the Intracellular Modulation of p97 (VCP) Protein-Protein Interactions. *J. Am. Chem. Soc.* **2022**, *144*, 13218-13225.
