# Supplementary material for: Autophagy Receptor-Inspired Antibody-Fusion Proteins for Targeted Intracellular Degradation
Source: J Am Chem Soc. Author manuscript; Available in PMC 2024 Feb 5. (PMC10636752; doi:10.1021/jacs.3c05199)
Supplement: 2 [file NIHMS1934253-supplement-2.pdf]

ASSOCIATED CONTENT

Supporting Information

The Supporting Information is available free of charge at https://pubs.acs.org/doi/10.1021/jacs.3c05199.

Supplementary figures, methods, and sequences (PDF)

https://doi.org/10.1021/jacs.3c05199
J. Am. Chem. Soc. XXXX, XXX, XXX−XXX
